# Programmed cortical ER collapse drives selective ER degradation and inheritance in yeast meiosis

**DOI:** 10.1083/jcb.202108105

**Published:** 2021-10-18

**Authors:** George Maxwell Otto, Tia Cheunkarndee, Jessica Mae Leslie, Gloria Ann Brar

**Affiliations:** 1 Department of Molecular and Cell Biology, University of California, Berkeley, Berkeley, CA; 2 California Institute for Quantitative Biosciences, University of California, Berkeley, Berkeley, CA; 3 Center for Computational Biology, University of California, Berkeley, Berkeley, CA

## Abstract

The endoplasmic reticulum (ER) carries out essential and conserved cellular functions, which depend on the maintenance of its structure and subcellular distribution. Here, we report developmentally regulated changes in ER morphology and composition during budding yeast meiosis, a conserved differentiation program that gives rise to gametes. A subset of the cortical ER collapses away from the plasma membrane at anaphase II, thus separating into a spatially distinct compartment. This programmed collapse depends on the transcription factor Ndt80, conserved ER membrane structuring proteins Lnp1 and reticulons, and the actin cytoskeleton. A subset of ER is retained at the mother cell plasma membrane and excluded from gamete cells via the action of ER–plasma membrane tethering proteins. ER remodeling is coupled to ER degradation by selective autophagy, which relies on ER collapse and is regulated by timed expression of the autophagy receptor Atg40. Thus, developmentally programmed changes in ER morphology determine the selective degradation or inheritance of ER subdomains by gametes.

## Teaser

Otto et al. show that the cortical ER undergoes programmed collapse from the plasma membrane during meiosis. Collapse separates the ER into distinct populations and determines which regions are eligible for inheritance by gametes and which are degraded by mechanisms including Atg40-mediated autophagy.

## Graphical Abstract

**Figure:**
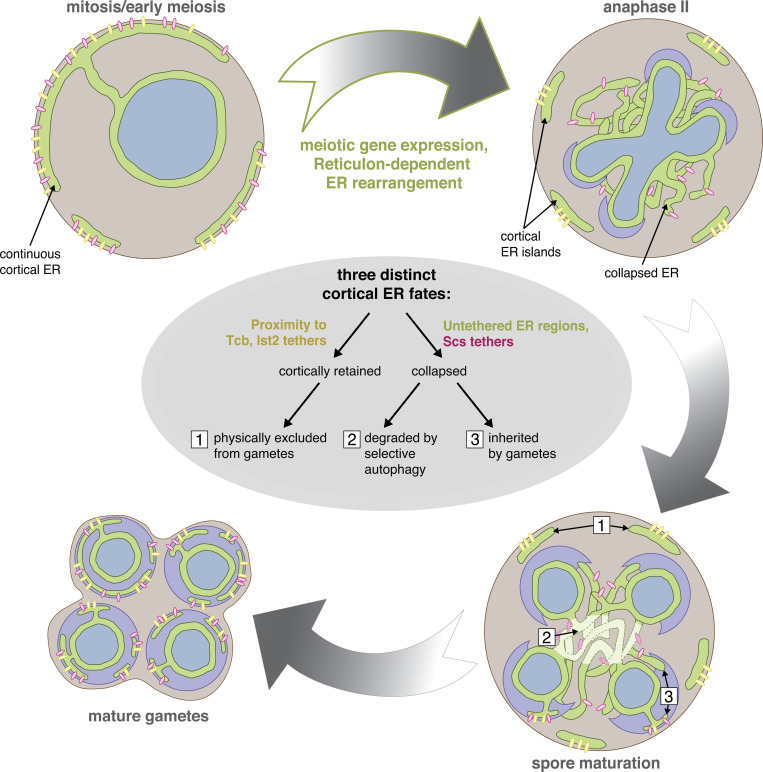

## Introduction

The ER is a membrane-bound organelle that carries out a range of essential and conserved cellular functions, including protein synthesis and trafficking, lipid metabolism, and interorganelle communication. These functions rely on the maintenance of ER structure and subcellular distribution, which are achieved through membrane-shaping proteins, fusion and fission of ER tubules, and tethering between the ER and other cellular structures, including organelles and the plasma membrane (PM; reviewed in Westrate et al., 2015; Schwarz and Blower 2016). ER structure is highly dynamic even in unperturbed cells and is dramatically remodeled in response to changes in cellular demand, such as protein folding stress or cell differentiation. Mutations that disrupt ER morphology are linked to a range of neurodegenerative diseases, including Alzheimer’s disease, amyotrophic lateral sclerosis, and hereditary spastic paraplegia (Renvoisé and Blackstone 2010; Öztürk et al., 2020), highlighting the intimate connection between ER structure and function, as well as the importance of ER quality control during cell differentiation.

The ER emanates from the nuclear envelope and localizes around the nucleus (perinuclear ER) as well as the cell periphery (cortical ER), where it forms extensive contacts with the PM. In budding yeast, ER-PM contacts are maintained by at least six tethering proteins, including Ist2; the tricalbins Tcb1, Tcb2, and Tcb3; and the vesicle-associated membrane protein–associated protein (VAP) orthologues Scs2 and Scs22 (Manford et al., 2012). All six tethers are integral ER membrane proteins that interact with phospholipids or proteins on the PM. Cells lacking these tethers have dramatically reduced cortical ER, disrupted lipid homeostasis, and acute sensitivity to ER stress, underscoring the importance of membrane tethering in maintaining ER structure and function. A second class of proteins involved in structuring the cortical ER is the reticulons and DP1/Yop1, which form wedge-like structures in the cytosolic leaflet of the ER membrane to promote membrane curvature and drive the formation of ER tubules (Voeltz et al., 2006; Hu et al., 2008). ER tubules are dynamic, constantly growing, retracting, and fusing with one another to generate three-way tubule junctions (Guo et al., 2018). Fusion is mediated by the dynamin-like GTPases Sey1 in budding yeast or Atlastin in metazoans (Hu et al., 2009; Orso et al., 2009; Anwar et al., 2012). Lunapark (Lnp) family proteins are involved in the maintenance of three-way junctions and display functional antagonism with Sey1/Atlastin, although the precise molecular role of Lnp in this process remains unclear (Chen et al., 2012; Chen et al., 2015; Wang et al., 2016). While factors that define ER structure are conserved across eukaryotes, we are only beginning to understand the diverse ways in which ER morphology and dynamics promote ER function.

Despite the fundamental relationship between ER structure and function, our knowledge of how the ER is remodeled as cells adapt to changing cellular conditions is limited. In budding yeast and cultured mammalian cells, exposure to chemical reducing agents causes ER protein folding stress and activation of the ER unfolded protein response, resulting in altered ER morphology and increased ER volume (Walter and Ron 2011; Schuck et al., 2009; Fumagalli et al., 2016). Both ER stress and nutrient starvation trigger selective degradation of the ER by autophagy (ERphagy), a response that is essential for cell adaptation and survival in these conditions (Mochida et al., 2015; Khaminets et al., 2015; Fumagalli et al., 2016; Zhang et al., 2020). While these studies provide crucial insight into ER quality control pathways that respond to cellular stress, few cases of programmed ER remodeling during natural development have been studied. Here, we leverage budding yeast meiosis to reveal rapid and naturally programmed ER remodeling in real time.

Meiosis is a conserved cell differentiation program that produces gametes through sexual reproduction. In meiosis, a diploid progenitor cell undergoes a single chromosome duplication event followed by homologue pairing, recombination, and two successive rounds of chromosome segregation, resulting in genetically distinct haploid cells. In addition to ensuring the proper distribution of chromosomes, cells undergoing meiosis must deliver a full complement of cellular components into gametes while preventing the inheritance of toxic or deleterious material (Neiman 2011; Goodman et al., 2020). While the regulation of meiotic chromosome segregation has been heavily studied, mechanisms governing the inheritance and elimination of other cellular components during meiosis are relatively poorly understood.

In this study, we define key steps and mechanisms in ER inheritance and quality control in budding yeast meiosis. We find that during meiosis most of the cortical ER collapses away from the PM, a process that depends on the meiotic transcription factor Ndt80, but not chromosome segregation. ER collapse relies on Lnp1, reticulons/Yop1, and the actin cytoskeleton. A subset of cortical ER is retained at the PM and excluded from gametes in an ER-PM tether–dependent manner. In late meiosis, the ER is subject to extensive degradation by a selective autophagy mechanism that requires cortical ER collapse. Together, our work defines a developmental quality control mechanism in which programmed changes in ER morphology determine both the inheritance and selective exclusion of ER subdomains by gamete cells.

## Results

### The ER detaches from the PM during meiosis

**Figure 1.**
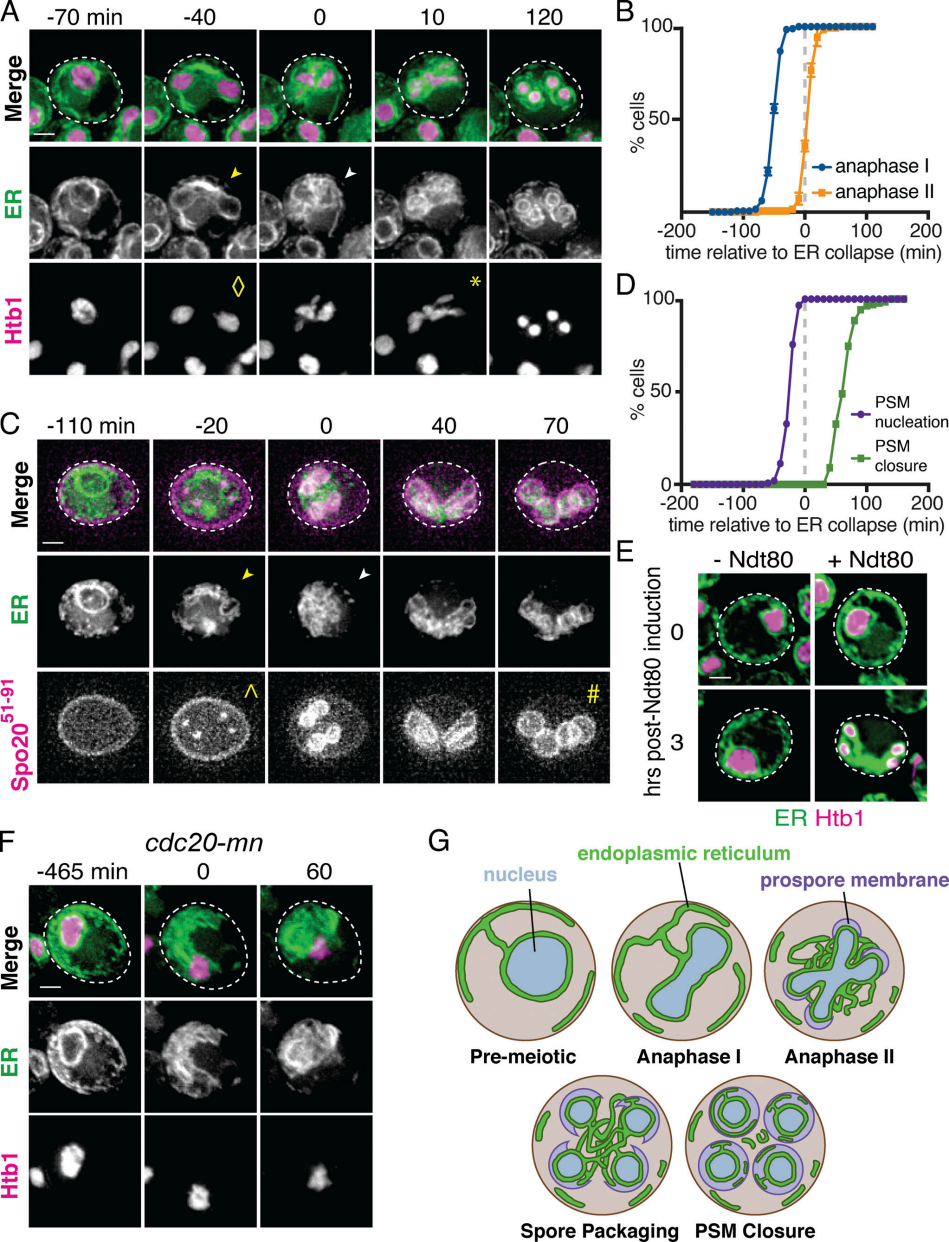
**The ER undergoes developmentally regulated structural remodeling during meiosis.** **(A)** Time-lapse microscopy of cells expressing GFP-HDEL to mark the ER and Htb1-mCherry to mark chromatin (Htb1) imaged every 10 min during meiosis. Symbols mark the onset of ER cabling (yellow arrowhead), ER collapse (white arrowhead), anaphase I (⋄), and anaphase II (*). ER collapse is defined to occur at 0 min. **(B)** Quantification of the time of anaphase I and anaphase II relative to ER collapse. **(C)** Time-lapse microscopy of cells expressing GFP-HDEL (ER) and mKate-Spo20^51–91^ to mark the prospore membrane (PSM). Symbols mark the onset of ER cabling (yellow arrowhead), ER collapse (white arrowhead), PSM nucleation (^), and PSM closure (#). **(D)** Quantification of the time of PSM nucleation and closure relative to ER collapse. **(E)** Cells expressing GFP-HDEL (ER), Htb1-mCherry (Htb1), and an estrogen-inducible allele of *NDT80* treated with 1 µM β-estradiol (+Ndt80) or vehicle (−Ndt80) after 5 h in SPO and imaged at the indicated times following induction. **(F)** As in A but in cells with the endogenous promoter of *CDC20* replaced with the mitosis-specific *CLB2* promoter (*cdc20-mn*), and cells imaged every 15 min. **(G)** Schematic of meiosis-coupled ER remodeling with relevant cellular structures and stages of meiosis and spore formation labeled. Where applicable, dashed white line denotes cell boundary. Scale bar = 2 µm for all panels.

**Figure S1.**
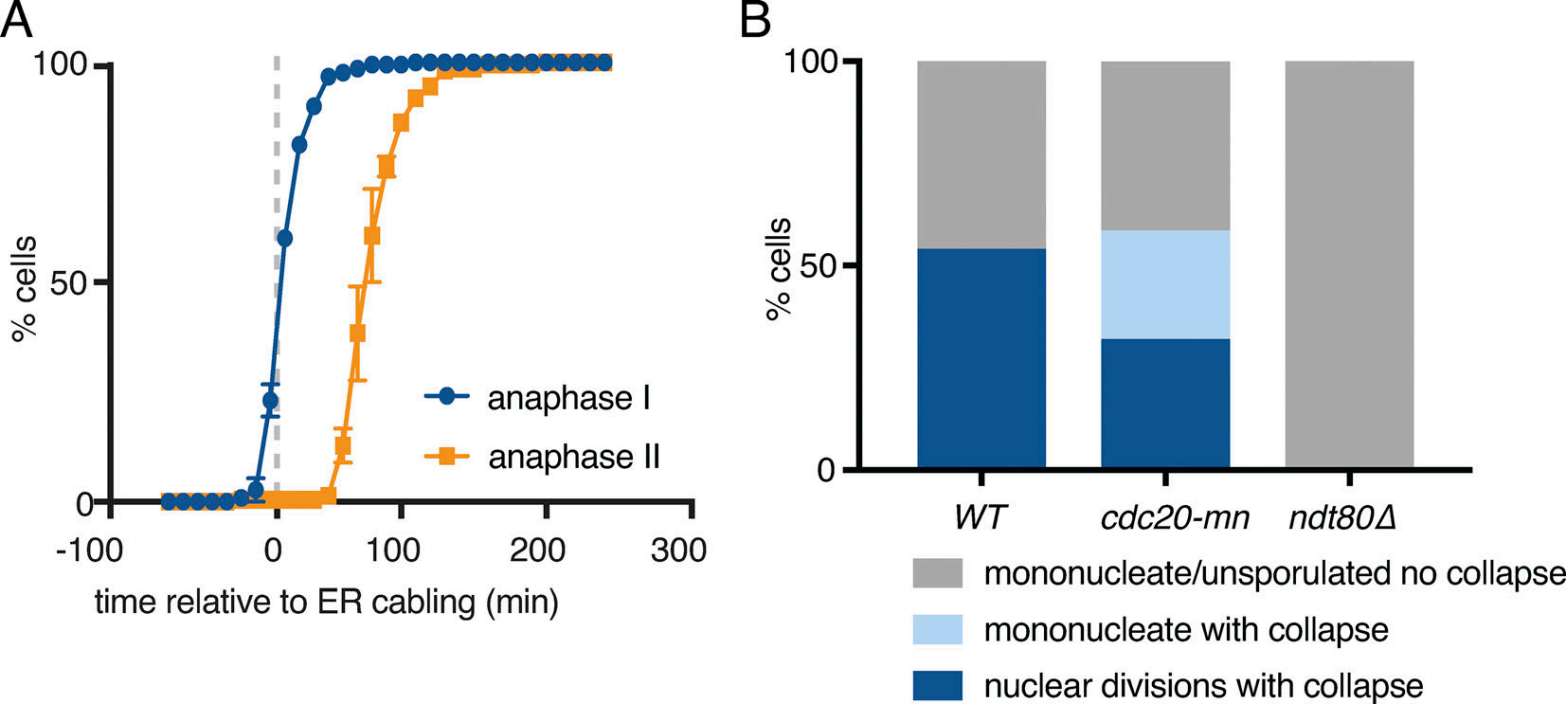
**The ER undergoes developmentally regulated structural remodeling during meiosis.** **(A)** Quantification of the time of anaphase I and anaphase II relative to ER cabling. **(B)** Quantification of cells within the indicated categories as assessed over the course of 16-h time-lapse videos. At least 100 cells were scored for each of the indicated genotypes. Differences between all groups are statistically significant (P < 0.0001 by χ^2^ test).

Meiotic differentiation involves regulated partitioning of organelles to ensure the development of healthy spores. To characterize ER dynamics during meiotic differentiation, we used time-lapse microscopy to monitor cells expressing fluorescent markers of the ER lumen (GFP-HDEL) and chromatin (Htb1-mCherry). Premeiotic cells displayed ER morphology that is characteristic of mitotic cells, with ER distributed around the cell periphery (cortical ER) and the nucleus (perinuclear ER). As cells progressed through meiosis, the cortical ER underwent a striking series of morphological changes. Early in meiosis, just before the first nuclear division, the cortical ER coalesced into bright, highly dynamic rope-like structures, a phenomenon we refer to as “ER cabling” (Figs. 1 A and S1 A and Video 1). Next, concurrent with anaphase II, ER detached from the cell periphery and abruptly relocalized to an area in the center of cells roughly bounded by the four gamete nuclei (Fig. 1, A and B; and Video 1). We refer to the abrupt detachment of cortical ER as “ER collapse,” a phenomenon that was previously predicted based on imaging of fixed cells in late meiotic stages but has not yet been studied in live cells (Suda et al., 2007). Finally, as spore packaging progressed, collapsed ER was inherited by each gamete and returned to the characteristic cortical and perinuclear structures seen in premeiotic cells (Fig. 1, A and G).

**Video 1.** **Time-lapse epifluorescence microscopy of the *WT* meiotic budding yeast cell depicted in ****Fig. 1 A****.** GFP-HDEL is shown in green and Htb1-mCherry in magenta. Cell is imaged over 14 h, with images collected every 10 min. Video shows 5 frames per second.

In budding yeast, meiosis is coupled to spore formation, in which gamete PMs (also called prospore membranes) are synthesized de novo and grow to encapsulate the full complement of cellular material to be inherited by gametes (Neiman 2011). Imaging the ER alongside a marker of prospore membrane synthesis, mKate-Spo20^51–91^ (Nakanishi et al., 2004), revealed that ER collapse takes place after prospore membrane nucleation but before closure (Fig. 1, C and D; and Video 2). Based on the timing of ER collapse and the spatial relationship between collapsed ER and nascent prospore membranes, it appears that cortical ER collapse is needed for its delivery into gamete cells.

**Video 2.** **Time-lapse epifluorescence microscopy of the *WT* meiotic budding yeast cell depicted in ****Fig. 1 C****.** GFP-HDEL is shown in green and mKate-Spo20^51–91^ in magenta. Cell is imaged over 14 h, with images collected every 10 min. Video shows 5 frames per second.

The precise timing with which ER detachment takes place relative to meiotic chromosome segregation and prospore membrane formation suggests that this process is tightly regulated as part of the broader developmental program that coordinates meiosis and spore formation. To further test this idea, we disrupted meiotic progression and assessed the impact on ER dynamics. First, we arrested cells in prophase I by withholding the meiotic transcription factor Ndt80, which is required to initiate the two meiotic nuclear divisions following homologous recombination (Chu and Herskowitz 1998; Benjamin et al., 2003). Arrested cells did not undergo ER cabling or collapse, indicating that these processes depend on Ndt80 induction and are not simply a response to the nutrient-poor conditions that stimulate meiosis in budding yeast (Figs. 1 E and S1 B). Blocking meiotic chromosome segregation using a meiotic null allele of the anaphase-promoting complex/cyclosome activator Cdc20 (*cdc20-mn*; Lee and Amon 2003), however, did not prevent cortical ER detachment from the PM. Cortical ER coalesced around the single, undivided nucleus in *cdc20-mn* cells, similar to what occurs around anaphase II in WT cells (Figs. 1 F and S1 B and Video 3). Together, these data indicate that meiotic ER remodeling is triggered by a developmental cue downstream of Ndt80 but is independent of chromosome segregation and the consequent dramatic changes to nuclear morphology.

**Video 3.** **Time-lapse epifluorescence microscopy of the *cdc20-mn* meiotic budding yeast cell depicted in ****Fig. 1 F****.** GFP-HDEL is shown in green and Htb1-mCherry in magenta. Cell is imaged over 10 h and 45 min, with images collected every 15 min. Video shows 3 frames per second.

### ER-PM tethers define a cortically retained ER compartment

**Figure 2.**
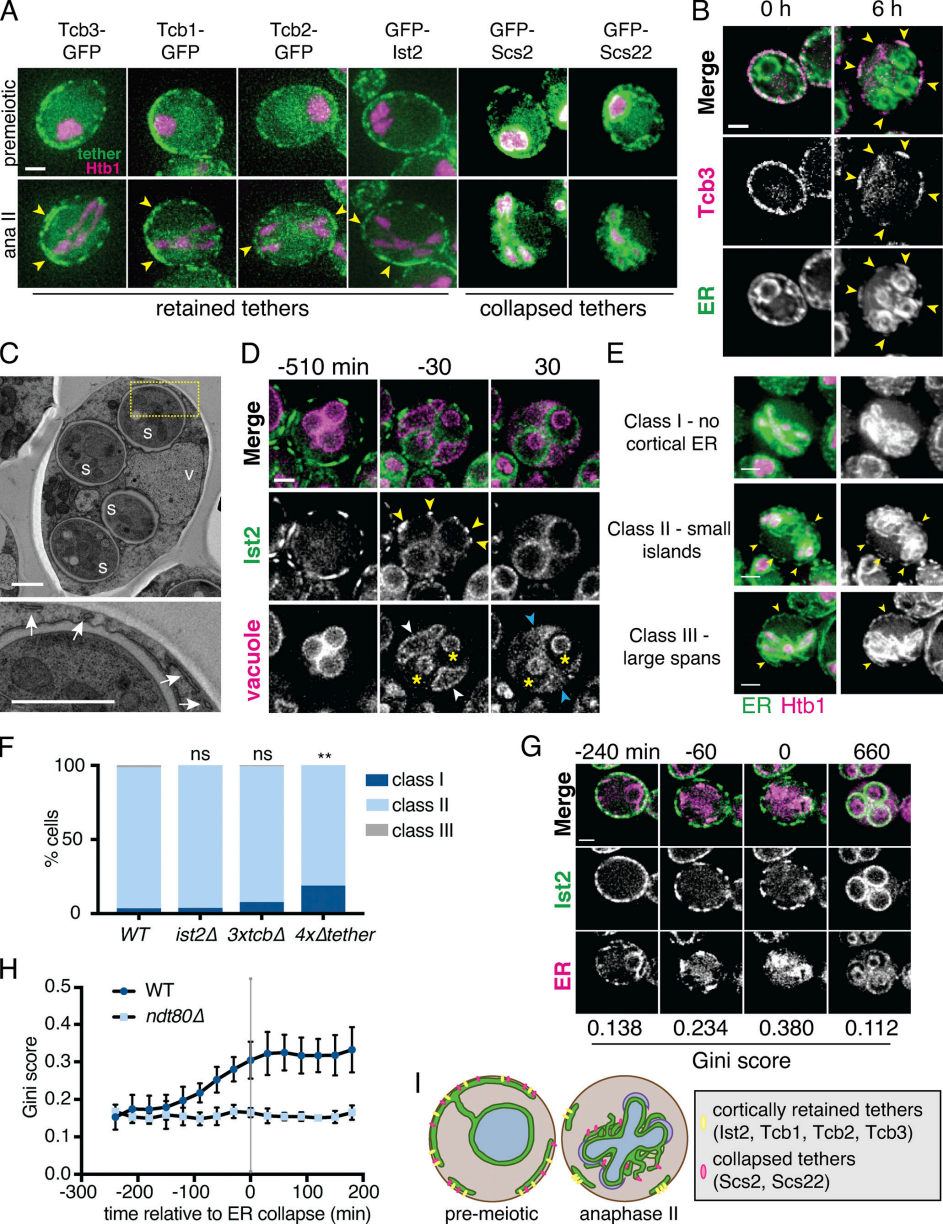
**A subset of ER-PM tethering proteins marks cortically retained ER islands.** **(A)** Time-lapse microscopy of cells expressing the indicated ER-PM tether tagged with GFP (tether) and Htb1-mCherry (Htb1) imaged during meiosis. A representative cell is shown before meiosis (top) and during anaphase II (ana II; bottom). Tethers are categorized as retained or collapsed based on anaphase II localization. Arrowheads highlight large stretches of cortical signal for each of the retained tethers. **(B)** Cells expressing GFP-HDEL (ER) and Tcb3-mKate (Tcb3) imaged at 0 h (top) and 6 h (bottom) after introduction to SPO. Arrowheads mark islands of colocalized ER lumen and Tcb3 signal. **(C)** EM of a WT cell following spore closure (top). Yellow box outlines the area shown zoomed in on the bottom. White arrows indicate cortically retained ER fragments. S marks the four spores. V marks the vacuole. **(D)** Time-lapse microscopy of cells expressing GFP-Ist2 and Vph1-mCherry (vacuole) imaged every 30 min in meiosis. White arrowheads mark Vph1 signal at the mother cell vacuole membrane, which becomes diffuse upon vacuolar lysis at time 0. Blue arrowheads mark diffuse Vph1 signal following vacuole lysis. Yellow asterisks mark spore vacuoles, which do not lyse. Yellow arrowheads mark bright clusters of Ist2 that are degraded upon vacuole lysis. **(E)** Images of cells expressing GFP-HDEL (ER) and Htb1-mCherry (Htb1) taken at anaphase II. A representative cell is shown for each cortical ER classification. Yellow arrowheads highlight cortically retained ER. **(F)** Quantification of at least *n* = 100 cells for the indicated genotypes following the classification system in E. P values determined by χ^2^ test comparing to WT. ns, P > 0.05; **, P < 0.01. **(G)** Time-lapse microscopy of cells expressing GFP-Ist2 and mCherry-HDEL (ER) imaged every 30 min in meiosis. The Gini score based on quantification of Ist2 signal is shown below each time point. Min 0 is defined as the time of ER collapse. **(H)** Gini quantification based on cortical Ist2 signal over time for the indicated genotypes. Values are the average of *n* = 10 *WT* or *n* = 3 *ndt80Δ* cells scored across each time point. Error bars represent SD. Gini score is significantly higher for WT than *ndt80Δ* for all time points starting at −90 min onward (P < 0.05 by Student’s *t* test). **(I)** Schematic showing ER morphology and tether localization in premeiotic and anaphase II cells. Scale bar = 2 µm for all panels except C, for which scale bar = 1 µm.

How is the abrupt detachment of the ER from the PM achieved? In budding yeast, at least six proteins function as ER-PM tethers. These include Ist2; the tricalbins Tcb1, Tcb2, and Tcb3; and the VAP orthologues Scs2 and Scs22. Cells lacking all six tethers have drastically reduced levels of cortical ER, disrupted lipid homeostasis, and reduced tolerance to ER stress (Manford et al., 2012). We sought to determine the role of ER-PM tethering proteins in meiotic ER collapse by imaging each tether during meiosis. To our surprise, endogenously tagged versions of Ist2 and all three tricalbins remained cortically localized throughout meiosis, even during anaphase II when cortical ER has collapsed (Fig. 2 A and Videos 4, 5, 6, and 7). In contrast, Scs2 and Scs22 demonstrated localization primarily to collapsed ER at anaphase II (Fig. 2 A).

**Video 4.** **Time-lapse epifluorescence microscopy of Tcb3-GFP in green and Htb1-mCherry in magenta in the *WT* meiotic budding yeast cell depicted in ****Fig. 2 A****.** Cell is imaged over 16 h, with images collected every 10 min. Video shows 5 frames per second.

**Video 5.** **Time-lapse epifluorescence microscopy of Tcb1-GFP in green and Htb1-mCherry in magenta in the *WT* meiotic budding yeast cell depicted in ****Fig. 2 A****.** Cell is imaged over 16 h, with images collected every 10 min. Video shows 5 frames per second.

**Video 6.** **Time-lapse epifluorescence microscopy of Tcb2-GFP in green and Htb1-mCherry in magenta in the *WT* meiotic budding yeast cell depicted in ****Fig. 2 A****.** Cell is imaged over 16 h, with images collected every 10 min. Video shows 5 frames per second.

**Video 7.** **Time-lapse epifluorescence microscopy of GFP-Ist2 in green and Htb1-mCherry in magenta in the *WT* meiotic budding yeast cell depicted in ****Fig. 2 A****.** Cell is imaged over 16 h, with images collected every 10 min. Video shows 5 frames per second.

**Figure S2.**
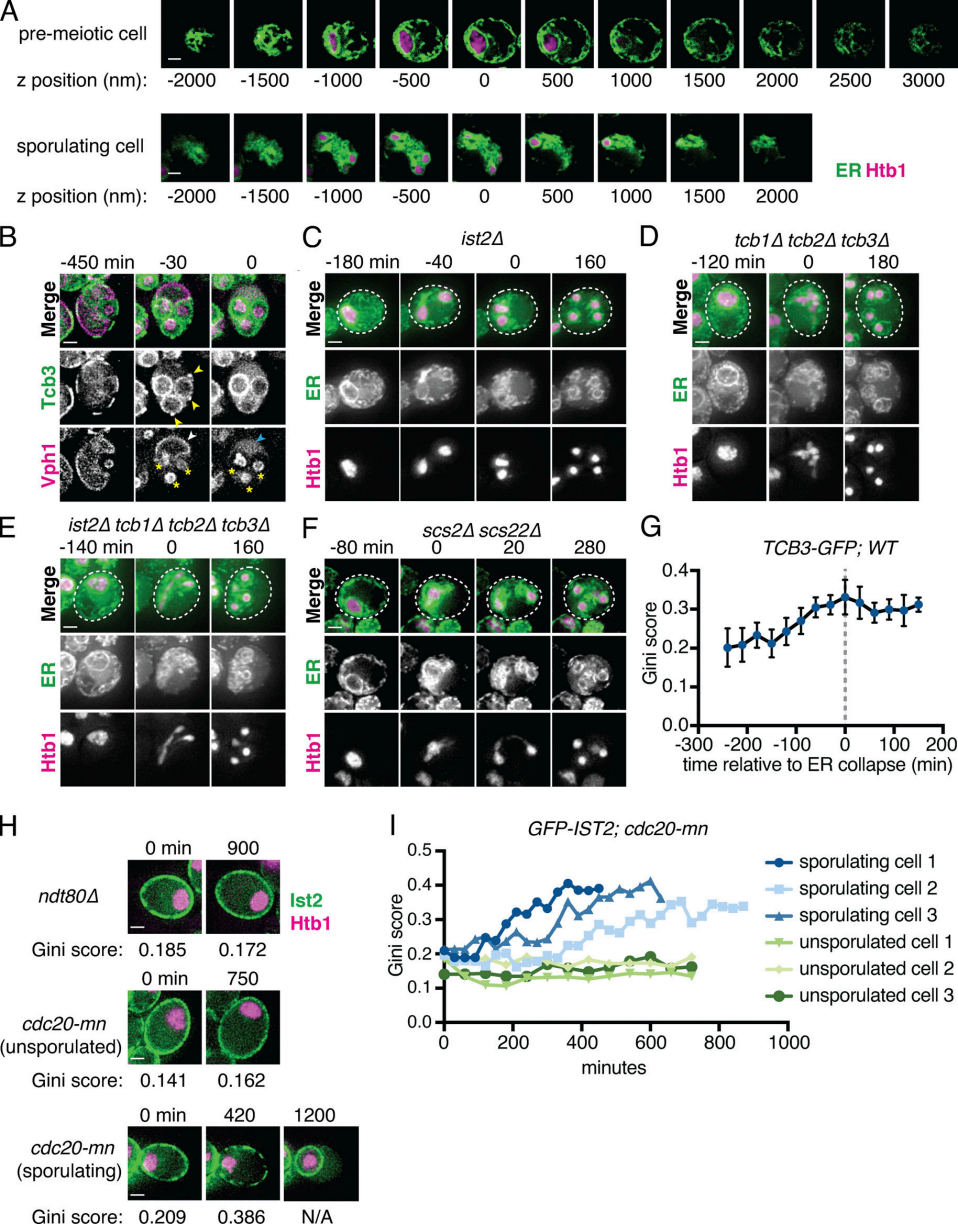
**A subset of ER-PM tethering proteins marks cortically retained ER islands.** **(A)** Z-series confocal microscopy of cells expressing GFP-HDEL (ER) and Htb1-mCherry (Htb1) taken before meiosis (top) and following meiotic ER collapse (bottom). **(B)** Time-lapse microscopy of cells expressing Tcb3-GFP and Vph1-mCherry imaged every 30 min during meiosis. Min 0 is defined as the time of vacuole lysis. White arrowheads mark Vph1 signal at the mother cell vacuole membrane, which becomes diffuse upon vacuolar lysis at time 0. Blue arrowhead indicates diffuse Vph1 signal following lysis. Yellow asterisks mark spore vacuoles, which do not lyse. Yellow arrowheads mark bright Tcb3 islands that are degraded upon vacuole lysis. **(C–F)** Time-lapse microscopy of cells of the indicated genotypes expressing GFP-HDEL (ER) and Htb1-mCherry imaged every 10 min during meiosis. Min 0 is defined as the time of ER collapse. **(G)** Gini quantification based on cortical Tcb3 signal over time. Values are the average of five cells scored across each time point. Error bars represent SD. **(H)** Time-lapse microscopy of cells of the indicated genotypes expressing GFP-Ist2 and Htb1-mCherry. Gini score is shown below each image. The 1,200-min time point for the sporulating *cdc20-mn* cell shows a single spore formed in the absence of chromosome segregation. **(I)** Gini quantification based on cortical Ist2 signal over time for six representative *cdc20-mn* cells, three of which ultimately sporulated and three of which failed to sporulate. Scale bar = 2 µm for all panels.

The cortical retention of a subset of ER-PM tethers was unexpected because all four proteins have integral membrane domains anchoring them in the ER and are therefore predicted to localize with the ER. We wondered if the cortically retained ER-PM tethers represented a previously overlooked subset of ER that failed to detach from the PM during ER collapse. Imaging Tcb3-mKate alongside GFP-HDEL revealed that Tcb3 signal at the cell cortex indeed overlapped with small islands of ER lumen, even when the vast majority of the ER was collapsed (Fig. 2 B). Analysis of previously published EM of meiotic cells revealed small “islands” of ER that remained bound to the PM even after prospore membrane closure (Fig. 2 C; King et al., 2019). 3D reconstruction of cells with collapsed ER revealed connections between retained ER islands, reflecting a sparse cortical ER network that surrounds the larger collapsed pool of cortical ER (Fig. S2 A and Videos 8 and 9). Together, our observations indicate that a subset of ER-PM tethers define a previously unappreciated cortically retained ER compartment.

**Video 8.** **3D reconstruction of z-stacks for the premeiotic *WT* budding yeast cell depicted in ****Fig. S2 A****. **GFP-HDEL is shown in green and Htb1-mCherry in magenta. Video shows 5 frames per second.

**Video 9.** 3**D reconstruction of z-stacks for the late meiotic *WT* budding yeast cell depicted in ****Fig. S2 A****.** GFP-HDEL is shown in green and Htb1-mCherry in magenta. Video shows 5 frames per second.

Because the gamete PM is formed de novo rather than inherited from the progenitor cell, any cellular component that is attached to the progenitor PM is necessarily excluded from gametes. We observed an abrupt decrease in the signal of all four excluded tethers in late meiosis, suggesting that excluded ER islands are degraded during this time (Videos 4, 5, 6, and 7). Late in meiosis, the yeast vacuole dramatically expands before ultimately lysing, releasing its contents into the ascoplasm region outside of spores and rapidly degrading the excluded material, including protein aggregates and nuclear pore complexes (Eastwood et al., 2012; King et al., 2019). To see if this is also the mechanism responsible for eliminating cortically retained ER, we performed time-lapse imaging of cells expressing Vph1-mCherry along with Tcb3-GFP or GFP-Ist2. Vacuole lysis, indicated by a switch in mCherry signal from vacuole membrane localized to diffuse (represented by the transition between the −30-min and 0-min panels in Fig. 2 D; King et al., 2019; Eastwood et al., 2012), coincided in timing with the disappearance of cortical ER signal in the former mother cell (*n* = 100 cells), mimicking the pattern seen for other targets of vacuolar lysis and supporting a model in which the release of vacuolar proteases into the ascoplasm is responsible for the degradation of cortically retained ER (Figs. 2 D and S2 B and Video 10; King et al., 2019; Eastwood et al., 2012). Thus, cortical ER retention is a means by which cells can exclude certain parts of the ER from gamete cells and subsequently degrade them.

**Video 10.** **Time-lapse epifluorescence microscopy of the *WT* meiotic budding yeast cell depicted in ****Fig. 2 D****.** GFP-Ist2 is in green and Vph1-mCherry in magenta. Cell is imaged over 30 h, with images collected every 30 min. Video shows 5 frames per second.

### ER-PM tethers promote the cortical retention of ER islands during ER collapse

Cortically retained ER islands appear to represent a small portion of the cell’s total ER pool. Nonetheless, we observed cell-to-cell heterogeneity in the amount of retained cortical ER in our live-cell microcopy experiments. To quantify this heterogeneity, and to enable us to assess the effect of genetic manipulation on cortical ER retention, we classified cells into three distinct groups: class I (no discernable ER retention), class II (small cortical ER islands), and class III (large spans of cortical ER; Fig. 2 E). The vast majority of WT cells scored at anaphase II of meiosis fell into class II, although we did observe a small number of class I and class III cells (Fig. 2 F). Deletion of the four cortically retained tethers (*4xΔtether*) resulted in a significant increase in the frequency of cells falling into class I and fewer cells in class II relative to WT cells and cells lacking only a subset of these tethers (Fig. 2 F and Fig. S2, C–E). As expected, deletion of *SCS2* and *SCS22*, the genes encoding the two tethers that follow collapsed ER, did not affect ER collapse or cortical ER retention (Fig. S2 F). We conclude that Ist2 and the tricalbins promote the exclusion and subsequent degradation of cortical ER islands in meiosis.

### Clustering of ER-PM tethers precedes ER collapse

We noted that although the cortically retained ER-PM tethers did not undergo collapse with the bulk of the ER, their localization was not static over time. Early in meiosis, tether signal was distributed largely homogeneously around the cell cortex. However, this pattern changed as meiosis progressed, with tether signal becoming more clustered, resulting in tether-rich islands separated by stretches of cell cortex with no tether signal (Fig. 2 G and Video 11). To quantitatively assess the degree to which tether signal is asymmetrically distributed within the cell cortex, we employed a metric called the Gini coefficient (*G*), which measures inequality within a dataset on a scale from 0 to 1 (Rouskin et al., 2014; Wittebolle et al., 2009). If a tether signal were distributed perfectly evenly throughout the cell cortex, it would receive a Gini coefficient of 0, whereas a highly asymmetric distribution of signal would be closer to 1. Cells in early meiosis had a relatively low Gini coefficient for GFP-Ist2 distribution (*G* = 0.153 ± 0.034; Fig. 2, G and H). This value steadily increased over time before plateauing (*G* = 0.304 ± 0.049) at the time of ER collapse. Qualitatively similar patterns were observed for all tricalbins and quantified for Tcb3 (Figs. 2 A and S2 G). Tether clustering is absent in *ndt80Δ* cells, which arrest before the nuclear divisions and also lack cortical ER collapse (Figs. 1 E, 2 H, and S2 H). As with ER collapse, blocking nuclear division in *cdc20-mn* cells did not prevent tether clustering (Fig. 1 F and Fig. S2, H and I). Together, these analyses demonstrate that the onset of tether clustering represents an early programmed step in meiotic ER remodeling, normally preceding ER collapse by several hours. Although the exact relationship between tether clustering and ER collapse is still unclear, our observations support a model in which the cortical ER is sorted into specific tether-containing (cortically retained) and tether-free (collapsed) domains to allow selective ER retention and inheritance (Fig. 2 I).

**Video 11.** **Time-lapse epifluorescence microscopy of the *WT* meiotic budding yeast cell depicted in ****Fig. 2 G****.** GFP-Ist2 is in green and mCherry-HDEL in magenta. Cell is imaged over 16 h, with images collected every 30 min. Video shows 5 frames per second.

### Reticulons promote ER detachment

How is the normally continuous cortical ER separated into collapsed and retained pools? One means of separating a continuous compartment into two separate topologies is by membrane fission, a phenomenon underlying key biological processes such as endocytosis, mitochondrial division, and cytokinesis. While the molecular mechanisms driving many membrane fission events are well characterized, the regulation of membrane fission in the ER is relatively poorly defined. Nevertheless, a growing body of evidence supports a role for membrane curvature in driving ER tubule fission in vitro and in vivo. ER membrane curvature is regulated by reticulons, a conserved class of proteins that generate ER membrane curvature via a double-hairpin reticulon homology domain (Voeltz et al., 2006; Hu et al., 2008). Overexpression of reticulon proteins results in ER fragmentation in cell culture and *Drosophila* models, while in vitro reconstituted ER networks containing reticulons spontaneously fragment in the absence of fusion-promoting factors (Wang et al., 2016; Powers et al., 2017; Espadas et al., 2019).

**Figure S3.**
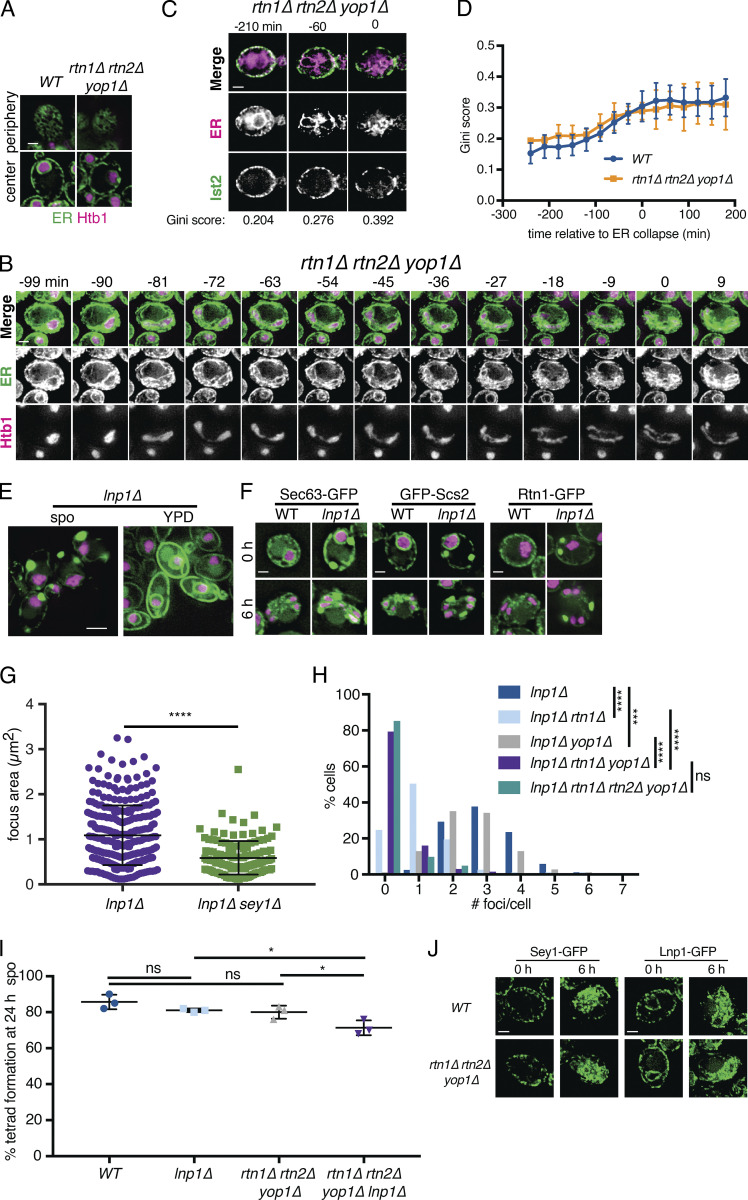
**Reticulons and Lnp1 regulate meiotic ER remodeling.** **(A)** WT and *rtn1Δ rtn2Δ yop1Δ* cells expressing GFP-HDEL (ER) and Htb1-mCherry imaged at the cell periphery and cell center immediately after transfer to SPO. **(B)** Time-lapse microscopy of *rtn1Δ rtn2Δ yop1Δ* cells expressing GFP-HDEL (ER) and Htb1-mCherry imaged every 3 min in meiosis. Min 0 is defined as the time of ER collapse. **(C)** Time-lapse microscopy of *rtn1Δ rtn2Δ yop1Δ* cells expressing GFP-Ist2 and mCherry-HDEL (ER) imaged every 30 min in meiosis. Min 0 is defined as the time of ER collapse. Gini score for Ist2 distribution is shown below each time point. **(D)** Gini quantification for at least *n* = 4 cells of the indicated genotypes. The average and SD are shown. **(E)** *lnp1Δ* cells expressing GFP-HDEL (ER) and Htb1-mCherry imaged immediately following transfer to SPO or during exponential growth in YPD. **(F)** WT and *lnp1Δ* cells expressing Htb1-mCherry and the indicated GFP-tagged protein imaged 0 or 6 h after transfer to SPO. **(G)** Average and SD quantifying ER focus size for at least *n* = 100 cells of each of the indicated genotypes. P values calculated by Student’s *t* test. ****, P < 0.0001. **(H)** Quantification of the number of foci per cell for of least *n* = 100 cells of the indicated genotypes. P values calculated by Student’s *t* test. ns, P > 0.05; ***, P < 0.001; ****, P < 0.0001. **(I)** Average and SD quantifying percentage tetrad formation scored 24 h after transfer to SPO for the indicated genotypes. *n* = 3 replicates were counted, with ≥100 cells each time. P values calculated by Student’s *t* test. *, P < 0.05. **(J)** WT or *rtn1Δ rtn2Δ yop1Δ* cells expressing Sey1-GFP or Lnp1-GFP at the indicated times following transfer to SPO. Scale bar = 2 µm for all panels.

**Figure 3.**
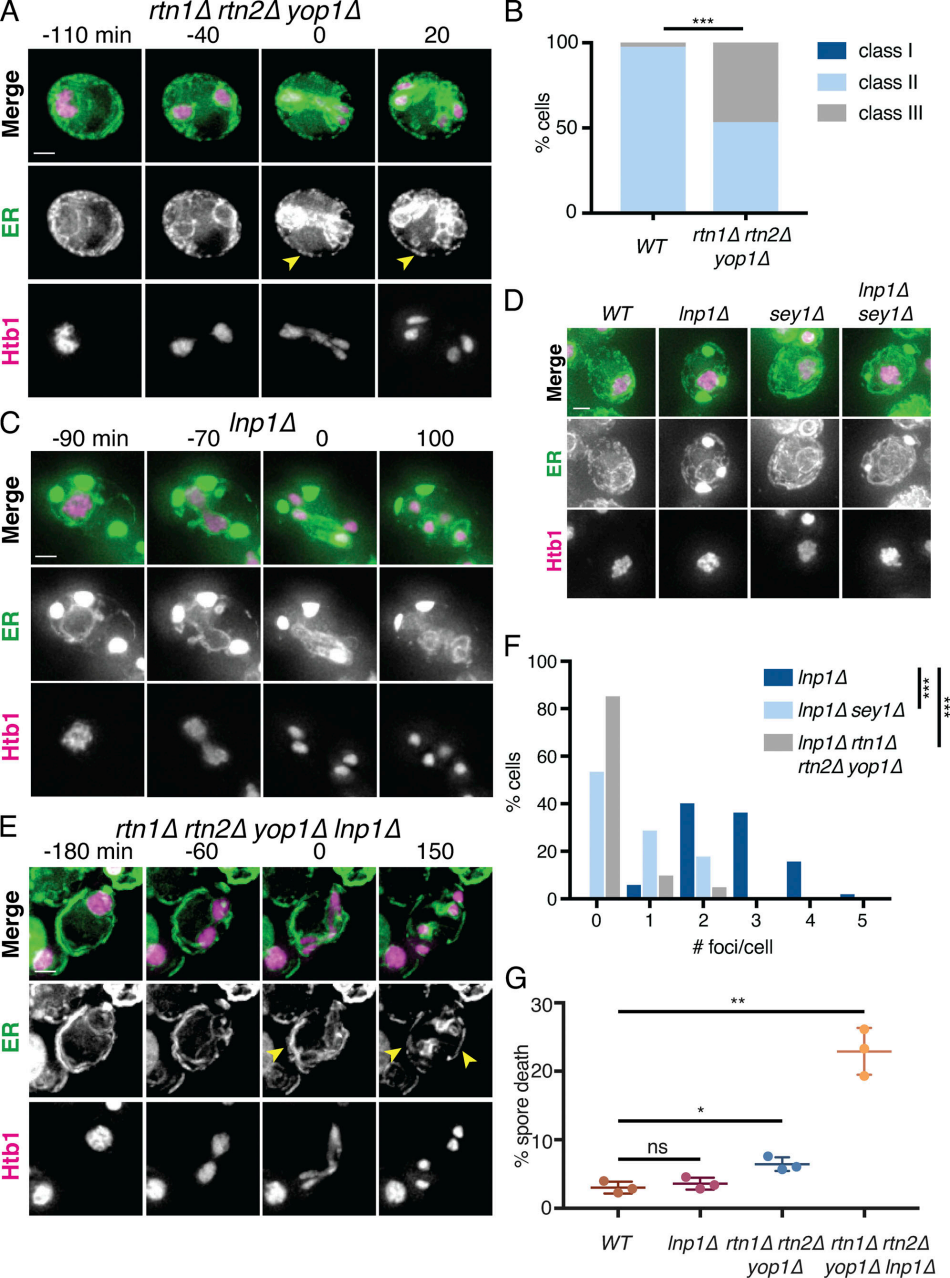
**Reticulons and Lnp1 regulate meiotic ER remodeling.** **(A)** Time-lapse microscopy of *rtn1Δ rtn2Δ yop1Δ* cells expressing GFP-HDEL (ER) and Htb1-mCherry imaged every 10 min during meiosis. Min 0 is defined as the time of ER collapse. Yellow arrowheads indicate abundant cortically retained ER persisting after anaphase II. **(B)** Classification of cortical ER retention for at least *n* = 100 cells for the indicated genotypes. ***, P < 0.001 by χ^2^ test. **(C)** As in A but with cells of genotype *lnp1Δ.* **(D)** Cells of the indicated genotypes expressing GFP-HDEL and Htb1-mCherry taken at 4 h in SPO. **(E)** As in A but with cells of genotype *rtn1Δ rtn2Δ yop1Δ lnp1Δ*. **(F)** Quantification of the number of foci per cell for of least *n* = 100 cells of the indicated genotypes. ***, P < 0.001 by Student’s *t* test. **(G)** Spore viability quantification after 24 h in SPO followed by germination for 48 h on YPD. Each of the three replicates represents results for at least *n* = 176 individual spores. P values calculated by Student’s *t* test. ns, P > 0.05; *, P < 0.05; **, P < 0.01. Scale bar = 2 µm for all panels.

Budding yeast have two reticulons, Rtn1 and Rtn2, and a reticulon-like protein, Yop1, that together are required for normal ER tubule formation (Voeltz et al., 2006). As expected, *rtn1Δ rtn2Δ yop1Δ* mutants displayed a drastic reduction in ER tubules under mitotic growth conditions (Fig. S3 A). Additionally, in meiotic cells, we observed a striking increase in the amount of ER that remained cortically localized beyond anaphase II (Fig. 3, A and B; and Video 12). Relative to WT, *rtn1Δ rtn2Δ yop1Δ* cells were much less likely to have class II cortical ER at anaphase II and much more likely to fall into class III (Fig. 3 B). We observed no difference in tether clustering in *rtn1Δ rtn2Δ yop1Δ* cells (Fig. S3, C and D), but the cabling behavior that we observed in WT cells immediately before collapse was absent in *rtn1Δ rtn2Δ yop1Δ* cells, suggesting that membrane curvature and/or fission are important for ER cabling, and that the cabling process may promote cortical ER detachment (Figs. 3 A and S3 B and Video 13). Together, these observations support a role for reticulon-mediated membrane curvature in promoting meiotic ER collapse. Reticulons may mediate the topological separation of retained and collapsed ER by promoting membrane fission, but we cannot rule out the possibility that ER collapse is an indirect result of changes in ER membrane curvature in cells lacking Rtn1, Rtn2, and Yop1.

**Video 12.** **Time-lapse epifluorescence microscopy of the *rtn1Δ rtn2Δ yop1Δ* meiotic budding yeast cell depicted in ****Fig. 3 A****.** GFP-HDEL is shown in green and Htb1-mCherry in magenta. Cell is imaged over 14 h, with images collected every 10 min. Video shows 5 frames per second.

**Video 13.** **Time-lapse epifluorescence microscopy of the *rtn1Δ rtn2Δ yop1Δ* meiotic budding yeast cell depicted in ****Fig. S3 B****. **GFP-HDEL is shown in green and Htb1-mCherry in magenta. Cell is imaged over 4 h, with images collected every 3 min. Video shows 5 frames per second.

### Lnp1 is required for ER detachment

Both normal ER tubule fission in unperturbed cells and ER fragmentation upon reticulon overexpression can be countered by homotypic membrane fusion, which is performed by the dynamin-like GTPase Sey1 (Atlastin in plants and metazoans; Wang et al., 2016; Espadas et al., 2019; Hu et al., 2009; Orso et al., 2009; Anwar et al., 2012). Because Lnp1 antagonizes Sey1 activity in mitotic yeast cells (Chen et al., 2012), we reasoned that Lnp1 may promote ER collapse by negatively regulating Sey1-mediated ER membrane fusion. If this were the case, cells lacking Lnp1 would be expected to show increased ER retention.

To our surprise, and in contrast to mitotic cells, *lnp1Δ* mutants displayed massive cortical ER foci when placed in sporulation media (SPO; Figs. 3 C and S3 E). We examined multiple ER markers, including lumenal and transmembrane proteins, and found that all of them localized to large ER foci in *lnp1Δ* cells, indicating that these aberrant structures are generally representative of ER in this condition (Fig. S3 F). Consistent with a role for Lnp1 in promoting ER detachment, the large ER foci in *lnp1Δ* cells retained their cortical localization throughout meiosis and spore packaging, resulting in their exclusion from gamete cells (Fig. 3 C and Video 14). Foci in *sey1Δ lnp1Δ* double mutants were smaller and less abundant than those found in *lnp1Δ* mutants, suggesting that these phenotypes result from excessive Sey1-mediated membrane fusion (Fig. 3, D and F; and Fig. S3 G).

**Video 14.** **Time-lapse epifluorescence microscopy of the *lnp1Δ* meiotic budding yeast cell depicted in ****Fig. 3 C****.** GFP-HDEL is shown in green and Htb1-mCherry in magenta. Cell is imaged over 15 h and 10 min, with images collected every 10 min. Video shows 5 frames per second.

To determine the relationship between reticulons and Lnp1 in promoting ER collapse, we examined ER dynamics in the quadruple *lnp1Δ rtn1Δ rtn2Δ yop1Δ* mutant. These cells rarely formed ER foci, suggesting that foci normally comprise reticulated ER (Fig. 3, E and F). Analysis of intermediate mutants suggests that the loss of foci in *lnp1Δ* cells upon removal of curvature-inducing proteins is additive (Fig. S3 H). Strikingly, *lnp1Δ rtn1Δ rtn2Δ yop1Δ* cells showed a dramatic increase in cortical ER retention during anaphase II, with all observed cells falling into class III (Figs. 2 E and 3 E and Video 15). We also found that these mutant cells had reduced sporulation efficiency and a severe spore viability defect, whereas mutants lacking only Rtn1, Rtn2, and Yop1 had modestly reduced spore viability and *lnp1Δ* cells were unaffected (Figs. 3 G and S3 I). Together, these data reveal a role for the regulation of membrane shape and fusion in ensuring normal ER detachment during meiosis and, ultimately, the health of the gametes produced during this process.

**Video 15.** **Time-lapse epifluorescence microscopy of the *rtn1Δ rtn2Δ yop1Δ lnp1Δ* meiotic budding yeast cell depicted in ****Fig. 3 E****.** GFP-HDEL is shown in green and Htb1-mCherry in magenta. Cell is imaged over 15 h and 10 min, with images collected every 10 min. Video shows 5 frames per second.

### Artificial ER-PM tethering does not prevent ER collapse

**Figure 4.**
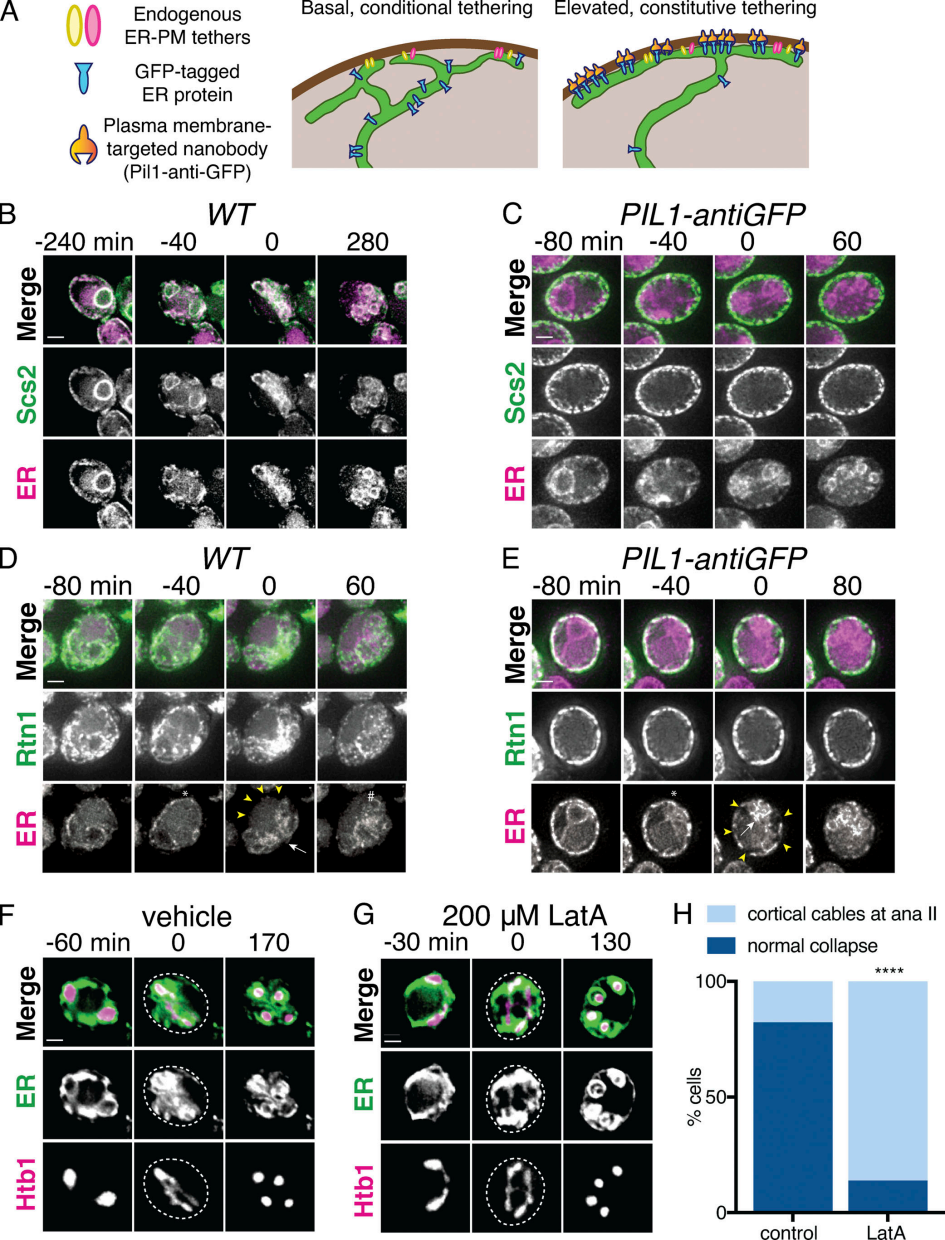
**Artificial cortical ER tethering does not prevent ER collapse.** **(A)** Schematic of artificial cortical ER tethering using Pil1-antiGFP. **(B)** Time-lapse microscopy of cells expressing GFP-Scs2 and mCherry-HDEL (ER) imaged every 10 min during meiosis. 0 min is defined as the time of ER collapse. **(C)** As in B but with cells expressing Pil1-antiGFP nanobody. **(D)** As in B but with cells expressing Rtn1-GFP instead of GFP-Scs2. Yellow arrowheads indicate cell cortex devoid of ER. White arrow indicates collapsed ER. **(E)** As in D but with cells expressing Pil1-antiGFP nanobody. Yellow arrowheads indicate small patches of cell cortex devoid of ER. White arrow indicates collapsed ER. **(F)** Cells expressing GFP-HDEL (ER) and Htb1-mCherry treated with DMSO (vehicle) at 4.5 h in meiosis and imaged every 10 min. 0 min is defined as the onset of anaphase II. Dashed white line denotes cell boundary. **(G)** As in F but cells were treated with 200 µM LatA instead of vehicle. Note the reduced ER mass in the cell center in G compared with F. **(H)** Quantification of cortical ER appearance for LatA-treated (*n* = 34) and untreated (*n* = 43) cells from the experiment shown in F and G. P values determined by χ^2^ test. ****, P < 0.0001. Scale bar = 2 µm for all panels. ana, anaphase.

Impaired ER collapse in cells lacking reticulons could result directly from reduced reticulon-dependent tubule severing or indirectly from altered ER morphology. We thus asked whether we could artificially tether cortical ER to the PM throughout meiosis without altering reticulon levels (Fig. 4 A). We constitutively tethered GFP-Scs2 to the cell cortex using the PM protein Pil1 fused to a genomically encoded antibody against GFP (Pil1-antiGFP; Schmit et al., 2018). Whereas GFP-Scs2 in WT cells localized with collapsed ER in anaphase II, GFP signal remained strictly cortical in cells expressing Pil1-antiGFP, indicating that our artificial tethering strategy was successful (Fig. 4, B and C; and Videos 16 and 17). To our surprise, this manipulation did not have an effect on cortical ER retention at anaphase II, as assessed by mCherry-HDEL localization, which was largely collapsed in late meiosis (57/57 Pil1-antiGFP cells, compared with 55/55 control cells). This result shows that introducing an artificial constitutive ER-PM tether does not prevent collapse, and that cortical release of the ER-PM tether Scs2 does not drive meiotic ER collapse.

**Video 16.** **Time-lapse epifluorescence microscopy of the meiotic budding yeast cell depicted in ****Fig. 4 B****.** GFP-Scs2 is shown in green and mCherry-HDEL in magenta. No Pil1-anti-GFP tether is present. Cell is imaged over 20 h, with images collected every 20 min. Video shows 5 frames per second.

**Video 17.** **Time-lapse epifluorescence microscopy of the meiotic budding yeast cell depicted in ****Fig. 4 C****.** GFP-Scs2 is shown in green and mCherry-HDEL in magenta. Pil1-anti-GFP tether is present. Cell is imaged over 13 h and 20 min, with images collected every 20 min. Video shows 5 frames per second.

**Figure S4.**
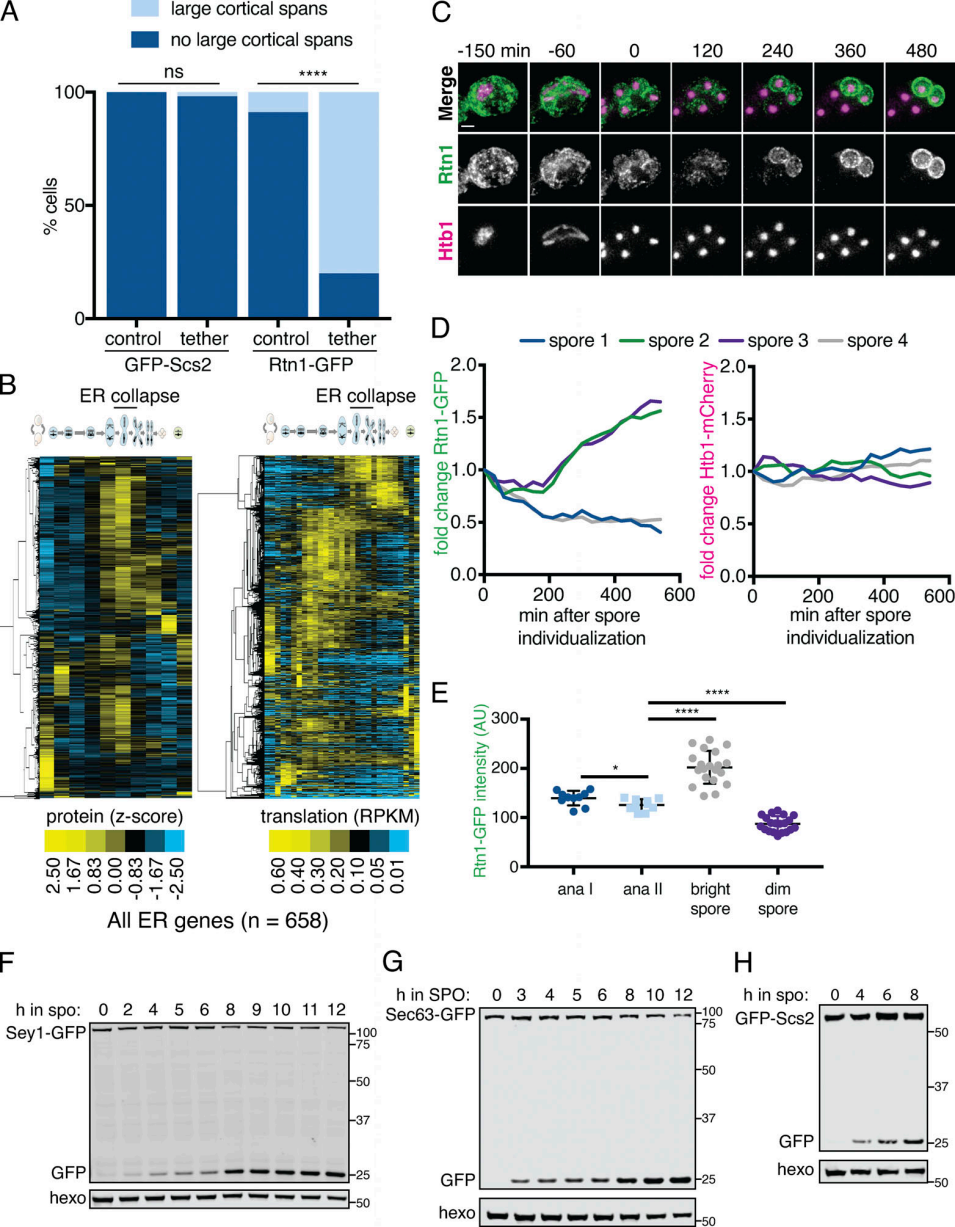
**A subset of the ER is degraded during meiosis and resynthesized in spores.** **(A)** Quantification of cortically retained ER in experiments in Fig. 4, B–E. P values determined by χ^2^ test comparing cells expressing the *PIL1-antiGFP* tethering construct to those that do not. At least 30 cells were counted per strain background. ns, P > 0.05; ****, P < 0.0001. **(B)** Hierarchical clustering of z-score quantification of published protein measurements (Cheng et al., 2018) for all quantified proteins annotated for ER localization. Each row represents one protein, and each column is a time point in meiosis. Protein levels are at left and translation levels at right. Degradation of a subset of almost every ER-localized protein can be seen during mid- to late meiosis in multiple waves, occurring concomitant with or after ER collapse. Of the 658 proteins characterized for ER localization, we also observed robust synthesis (>50 RPKM [reads per kilobase of transcript per million mapped reads]) for 81.3% of them, with this synthesis occurring late in meiosis (after ER collapse) for 85.2% of this set. **(C–E)** To independently assess the turnover of an abundant ER protein during meiosis, we used an assay that takes advantage of the diploid status of meiotic cells (Eisenberg et al., 2018) by imaging cells with heterozygous tags marking the ER (*RTN1-GFP/RTN1-WT*) and histones (*HTB1-mCherry/HTB1-WT*). In this system, preexisting ER is marked by Rtn1-GFP, whereas newly synthesized ER following spore closure will be either marked or unmarked, depending on if the spore inherited *RTN1-GFP* or *RTN1-WT*, respectively. Consistent with the data in B, two spores per tetrad retained high levels of GFP signal, while GFP levels in the other two spores progressively declined, as opposed to the persistence of mCherry signal (and thus histone presence) in all four spores. **(C)** Time-lapse microscopy of cells heterozygous for *RTN1-GFP* and *HTB1-mCherry* imaged every 30 min in meiosis. Time 0 is defined as the time of spore individualization. Scale bar = 2 µm. **(D)** Quantification of the average GFP (left) and mCherry (right) signal for each spore for the cell in C. The GFP signal decrease in two spores indicates Rtn1 degradation. **(E)** Quantification of the average GFP signal for *n* = 10 cells at anaphase (ana) I, ana II, and the last imaged time point separated based on signal brightness (bright spore and dim spore). P values calculated by Student’s *t* test. *, P < 0.05; ****, P < 0.0001. **(F–H)** Western blot with samples taken from cells expressing the indicated GFP-tagged ER protein, taken at the indicated times following transfer to SPO and probed for GFP and hexokinase. Source data are available for this figure: SourceData FS4.

We reasoned that forced tethering of a more abundant cortical ER protein may be necessary to prevent collapse, leading us to perform a similar approach using the reticulon protein Rtn1, which is normally prevalent throughout the cortical ER before and after its meiotic collapse (Fig. 4 D). Forced tethering of Rtn1-GFP increased the amount of cortically retained ER (Fig. S4 A), but ER collapse was still clearly detectable in the vast majority of cells (27/30, compared with 34/34 in controls), as assessed by a substantial amount of mCherry-HDEL signal in the collapsed ER pool at anaphase II and a corresponding reduction in cortical mCherry-HDEL signal (Fig. 4, D and E; and Videos 18 and 19). These results support a model in which the collapse or cortical retention of a given ER region is based on each region’s local association (or lack of association) with ER-PM tethers. Thus, introduction of an abundant artificial tether increases the amount of ER that is cortically retained but cannot prevent bulk ER dissociation from the PM.

**Video 18.** **Time-lapse epifluorescence microscopy of the meiotic budding yeast cell depicted in ****Fig. 4 D****.** GFP-Rtn1 is shown in green and mCherry-HDEL in magenta. No Pil1-anti-GFP tether is present. Cell is imaged over 18 h and 20 min, with images collected every 20 min. Video shows 5 frames per second.

**Video 19.** **Time-lapse epifluorescence microscopy of the meiotic budding yeast cell depicted in ****Fig. 4 E****.** GFP-Rtn1 is shown in green and mCherry-HDEL in magenta. Pil1-anti-GFP tether is present. Cell is imaged over 18 h and 20 min, with images collected every 20 min. Video shows 5 frames per second.

### The actin cytoskeleton promotes ER collapse

The abrupt, coordinated movement of cortical ER away from the PM suggests the involvement of a force-generating mechanism rather than passive diffusion. During mitosis in yeast, ER tubules are delivered into the daughter cell along actin cables (Estrada et al., 2003). To determine whether the actin cytoskeleton is also involved in meiotic ER dynamics, we treated cells undergoing meiosis with Latrunculin A (LatA), a drug that prevents actin polymerization. LatA-treated cells were still able to undergo ER cabling, but cabled structures failed to collapse, instead remaining cortical throughout chromosome segregation, suggesting that cabled ER is pulled away from the PM along actin filaments (Fig. 4, F–H and Videos 20 and 21).

**Video 20.** **Time-lapse epifluorescence microscopy of DMSO vehicle treated meiotic budding yeast cell depicted in ****Fig. 4 F****.** GFP-HDEL is shown in green and Htb1-mCherry in magenta. Cell is imaged over 12 h, with images collected every 10 min. Video shows 5 frames per second. Compare to Video 21.

**Video 21.** **Time-lapse epifluorescence microscopy of LatA treated meiotic budding yeast cell depicted in ****Fig. 4 G****.** Cell was treated with 200uM LatA. GFP-HDEL is shown in green and Htb1-mCherry in magenta. Cell is imaged over 12 h, with images collected every 10 min. Video shows 5 frames per second. Compare to Video 20.

### The ER is degraded by autophagy during meiosis

**Figure 5.**
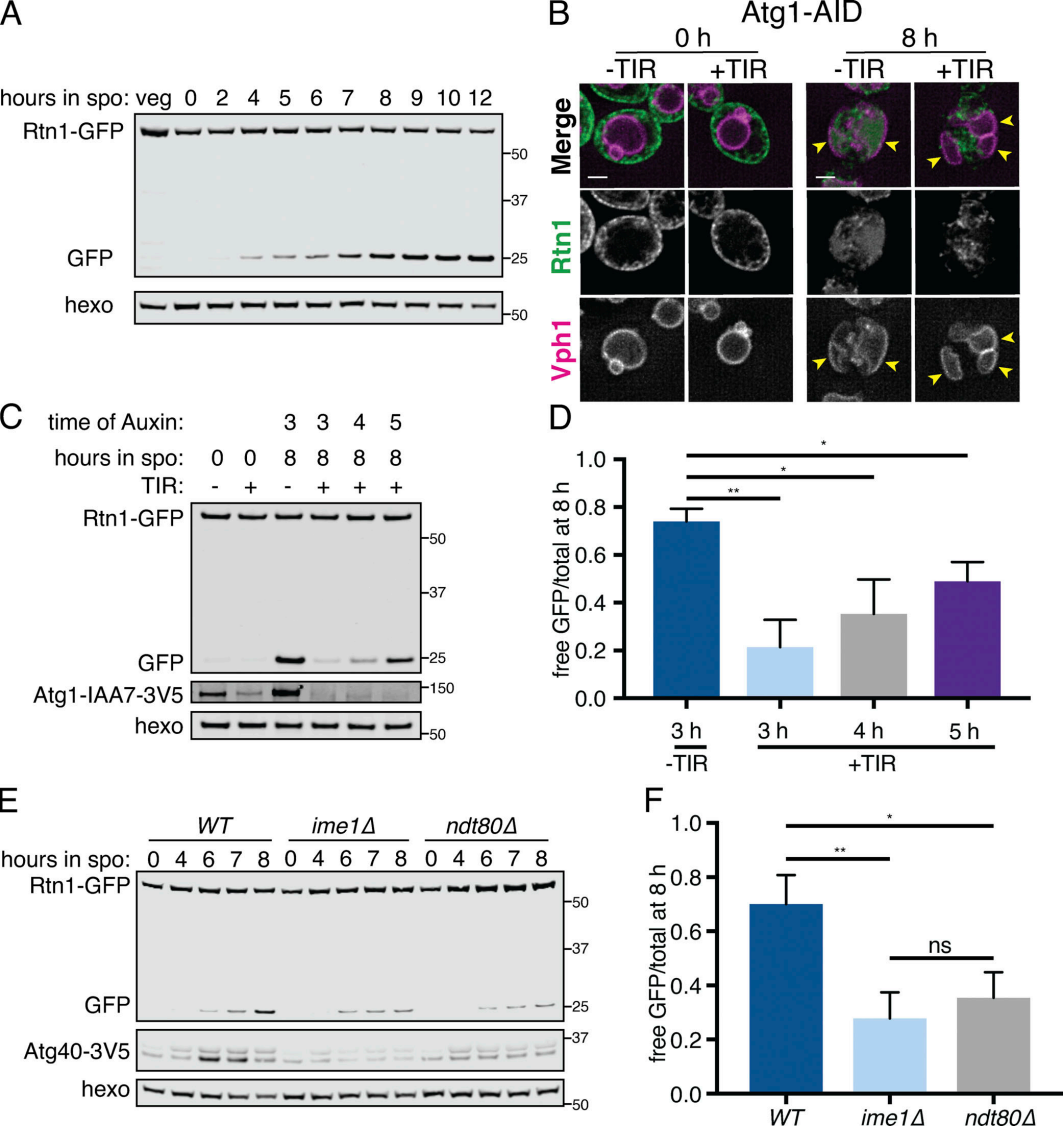
**The ER is degraded by autophagy during meiosis.** **(A)** Western blot with samples taken from cells expressing Rtn1-GFP during vegetative exponential growth (veg) or at the indicated time in meiosis probing for GFP and hexokinase (hexo) loading control. **(B)** Microscopy of cells expressing Rtn1-GFP, Vph1-mCherry, and Atg1-AID and imaged at the indicated times after transfer to SPO. Presence (+) or absence (−) of osTIR is indicated. Cells were treated with 500 µM auxin after 4 h in SPO. Yellow arrowheads indicate pockets of vacuole. **(C)** Western blot of cells of the same genotypes as in B probing for GFP, V5, and hexo. Cells were treated with 500 µM auxin at the indicated times. **(D)** Average and SD quantifying free GFP as a proportion of total GFP signal for *n* = 3 replicates of the experiment in C. P values calculated by Student’s *t* test. **(E)** Western blot with samples from cells of the indicated genotypes and expressing Rtn1-GFP and Atg40-3V5 taken at the indicated times after transfer to SPO, probed for GFP, V5, and hexo. **(F)** Average and SD quantifying free GFP as a proportion of total GFP signal for *n* = 3 replicates of the experiment in E. P values calculated by Student’s *t* test. ns, P > 0.05; *, P < 0.05; **, P < 0.01. Source data are available for this figure: SourceData F5.

**Figure S5.**
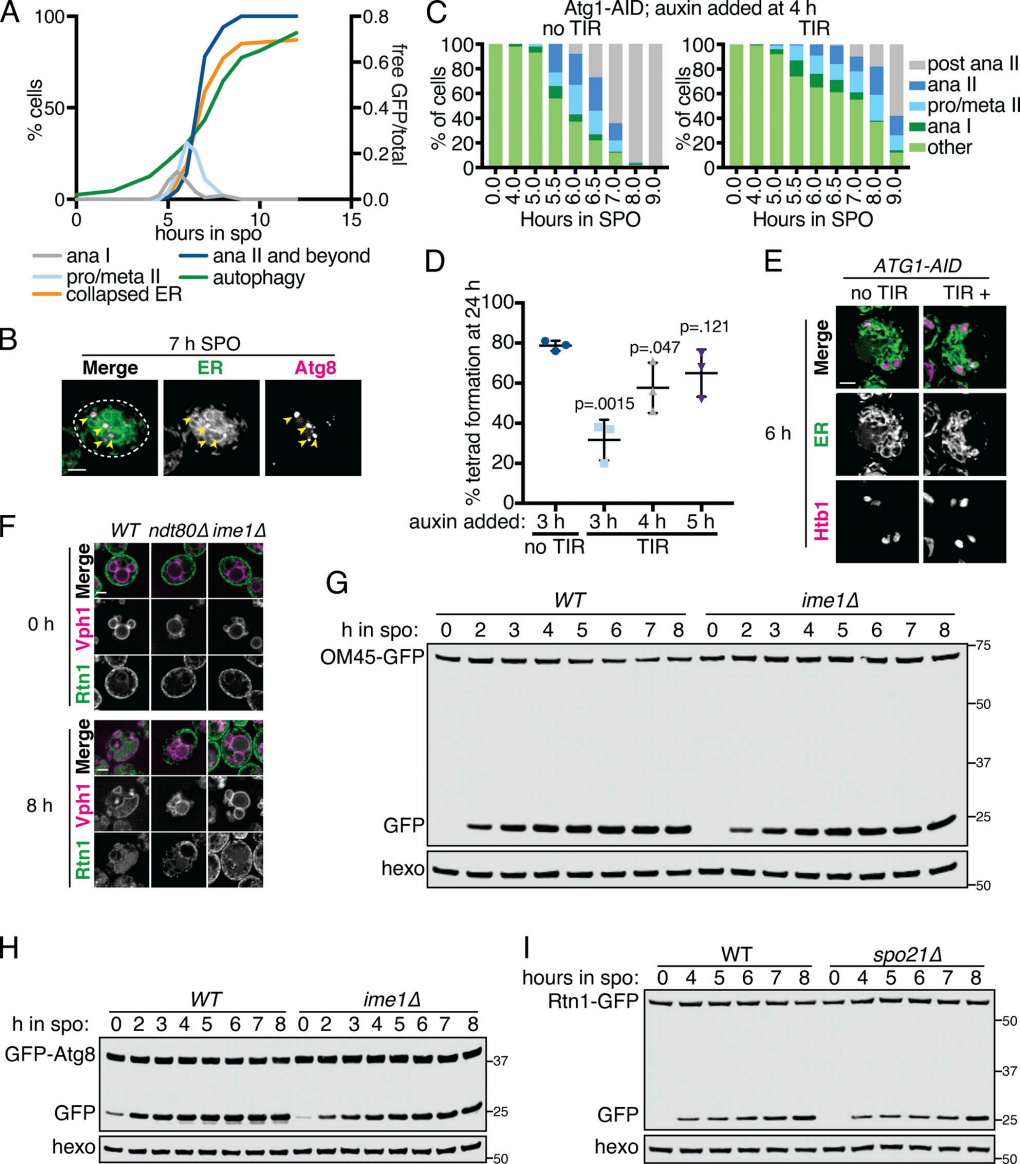
**The ER is degraded by autophagy during meiosis.** **(A)** Quantification of meiotic staging, ER collapse, and autophagy using samples taken in parallel to those in Fig. 5 A. Left axis shows the percentage of cells at the indicated stage in meiosis and right axis shows the free GFP signal as a proportion of the total (Rtn1-GFP + free GFP). ana, anaphase; meta, metaphase; pro, prophase. **(B)** Fluorescence microscopy of cells expressing mCherry-HDEL (ER; green channel) and GFP-Atg8 (Atg8; magenta channel) acquired 7 h following transfer to SPO. Arrowheads indicate overlapping Atg8 and ER signal. **(C)** Quantification of meiotic staging by spindle immunofluorescence using cells expressing Atg1-AID with or without the presence of TIR ligase. Auxin was added to cultures 4 h after introduction to SPO. Abbreviations as in A. **(D)** Average and SD quantifying percentage tetrad formation measured 24 h following introduction to SPO. *n* = 3 replicates with ≥100 cells counted each time. P values calculated using Student’s *t* test. **(E)** Fluorescence microscopy of cells expressing Atg1-AID along with GFP-HDEL (ER) and Htb1-mCherry acquired after 6 h in SPO. Auxin was added at 4 h. **(F)** Fluorescence microscopy of cells of the indicated genotypes expressing Rtn1-GFP and Vph1-mCherry acquired at the indicated times in meiosis. **(G)** Western blot using samples from WT and *ime1Δ* cells expressing OM45-GFP, taken at the indicated times during meiosis. Blots were probed for GFP and hexokinase. **(H)** As in G but with cells expressing GFP-Atg8 instead of OM45-GFP. **(I)** Western blot with samples taken from WT and *spo21Δ* cells expressing Rtn1-GFP at the indicated times in meiosis. Probed for GFP and hexokinase. Source data are available for this figure: SourceData FS5.

We previously found widespread turnover of most proteins during meiosis, based on global matched measurements for protein abundance and translation levels (Cheng et al., 2018; Eisenberg et al., 2018). This included almost every ER-localized protein, most of which were degraded with timing concomitant with or following ER collapse (Fig. S4, B–E). In investigating the mechanisms that may mediate ER turnover during meiosis, we considered macroautophagy (hereafter referred to as autophagy), in which cargo such as organelle fragments are engulfed by a double-membrane autophagosome and targeted to the vacuole (lysosome in metazoans) for degradation (Morishita and Mizushima 2019). General autophagy factors are highly up-regulated during meiosis, and the kinase Atg1 is essential for both autophagy and entry into meiosis (Brar et al., 2012; Wen et al., 2016). Because GFP is resistant to vacuolar proteases while cargo proteins are not, GFP-tagged proteins that have been degraded by autophagy leave behind a GFP epitope that can be readily detected by Western blot (Mochida et al., 2015). We tagged several ER-resident proteins with GFP and observed the accumulation of a GFP-only band in meiosis, suggesting that the ER as a whole is a target of autophagy during this process (Fig. 5 A and Fig. S4, F–H). A faint GFP fragment was visible as early as a few hours into meiosis, but the greatest accumulation occurred as cells progressed through anaphase II and beyond (Figs. 5 A and S5 A). As an orthogonal means of observing ER autophagy (ERphagy), we imaged cells expressing Rtn1-GFP and Vph1-mCherry, a marker of the vacuole membrane. Prior to meiosis, there was very little GFP within the vacuole, whereas cells in late meiosis displayed strong, diffuse GFP signal throughout the vacuole (Fig. 5 B). We also observed Atg8-marked autophagosomes colocalized with collapsed ER in late meiosis, providing further evidence that cells induce ERphagy as they progress through meiosis (Fig. S5 B).

Atg1 is required for entry into meiosis, so we could not assess the role of the canonical Atg1-dependent autophagy pathway in meiotic ERphagy using *atg1Δ* cells. Instead, we conditionally depleted cells of Atg1 after meiotic entry using the auxin-inducible degron system, in which TIR1, a plant-derived ubiquitin ligase, targets degron-bearing substrates for proteasomal degradation only in the presence of the plant hormone auxin (Nishimura et al., 2009). By withholding auxin until after meiotic entry, we were able to deplete cells of degron-tagged Atg1 (Atg1-AID) without completely blocking meiosis. Cells depleted of Atg1 had slowed meiotic progression and reduced sporulation efficiency, consistent with an important role for autophagy throughout the process of sporulation (Fig. S5, C and D). Nevertheless, we were able to observe Atg1-depleted cells in late meiosis with characteristic ER collapse and expanded vacuolar morphology but no vacuolar GFP signal (Figs. 5 B and S5 E). The accumulation of GFP as a lone fragment by Western blot in Rtn1-GFP cells was also strongly reduced by depletion of Atg1, an effect that was stronger the earlier cells were treated with auxin (Fig. 5, C and D). Thus, ERphagy in meiosis takes place through the canonical Atg1-dependent pathway.

We next sought to determine whether ERphagy is induced as part of the developmental program of meiosis or simply in response to SPO, which is nutrient poor. Cells progressing through meiosis induced ERphagy much more strongly than cells arrested in prophase I (*ndt80Δ*) or before meiotic entry (*ime1Δ*), indicating that this process is enhanced by meiotic progression (Fig. 5, E and F; and Fig. S5 F). Interestingly, ERphagy differs from other forms of autophagy in this respect. With the same experimental setup, we assessed general autophagy using GFP-Atg8 and mitochondrial autophagy (mitophagy) using OM45-GFP. Autophagy in general, and mitophagy in particular, were induced rapidly upon introduction into SPO, even when cells were arrested before meiotic entry (Fig. S5, G and H). *spo21Δ* cells, which progress late into meiosis but cannot form prospore membranes, show normal levels of ERphagy, suggesting that spore formation per se is not required for this process (Fig. S5 I). Together, these results indicate that cells perform autophagy throughout meiosis but prevent ERphagy until a later developmental stage. Because ERphagy has previously only been studied in the context of prolonged starvation or exposure to harsh chemical stress, it is intriguing to see its induction in a developmental context in which external stressors are absent.

### Meiotic ERphagy is mediated by selective autophagy receptors

**Figure 6.**
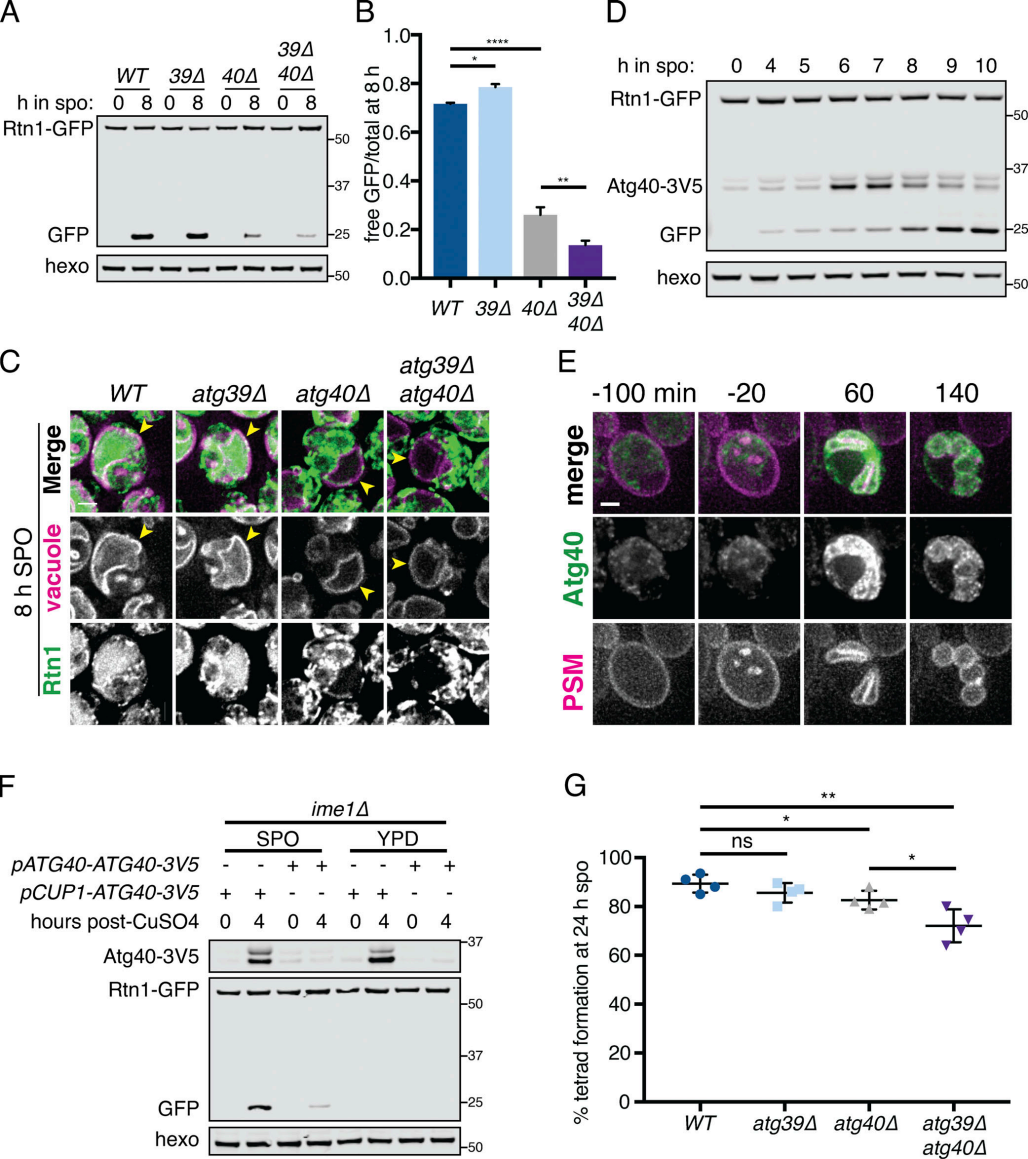
**Developmentally regulated Atg40 expression drives selective ERphagy in meiosis.** **(A)** Western blot with samples taken from *WT*,* atg39Δ*,* atg40Δ*, and *atg39Δatg40Δ* cells expressing Rtn1-GFP at the indicated times after transfer to SPO, probed for GFP and hexokinase. **(B)** Average and SD quantifying free GFP as a proportion of total GFP signal for *n* = 3 replicates of the experiment in A. P values calculated by Student’s *t* test. *, P < 0.05; **, P < 0.01; ****, P < 0.0001. **(C)** Microscopy of cells of the indicated genotypes expressing Rtn1-GFP and Vph1-mCherry and imaged at the indicated times after transfer to SPO. Scale bar = 2 µm. **(D)** Western blot with samples taken from cells expressing Rtn1-GFP and Atg40-3V5 at the indicated times after transfer to SPO, probed for GFP, V5, and hexokinase. **(E)** Time-lapse microscopy of cells expressing Atg40-3xGFP and mKate-Spo20^51–91^ (PSM) imaged every 10 min in meiosis. Note increased Atg40-3xGFP signal intensity in collapsed ER relative to earlier and later time points. Scale bar = 2 µm. **(F)** Western blot with samples taken from *ime1Δ* cells expressing Rtn1-GFP and Atg40-3V5 under the endogenous promoter (*pATG40-ATG40-3V5*) or the *CUP1* promoter (*pCUP1-ATG40-3V5*). For YPD samples, cells were diluted to 0.05 OD units in YPD, allowed to grow to exponential phase, and treated with 50 µM CuSO_4_. For SPO samples, cells were transferred to SPO for 2 h and treated with 50 µM CuSO_4_. Protein samples were taken at the indicated times after CuSO_4_ treatment. **(G)** Average and SD quantifying percentage tetrad formation for cells of the indicated genotypes measured 24 h following transfer to SPO. *n* = 4 replicates were performed, with ≥100 cells counted per replicate. P values calculated by Student’s *t* test as in B. ns, P > 0.05. Source data are available for this figure: SourceData F6.

**Figure S6.**
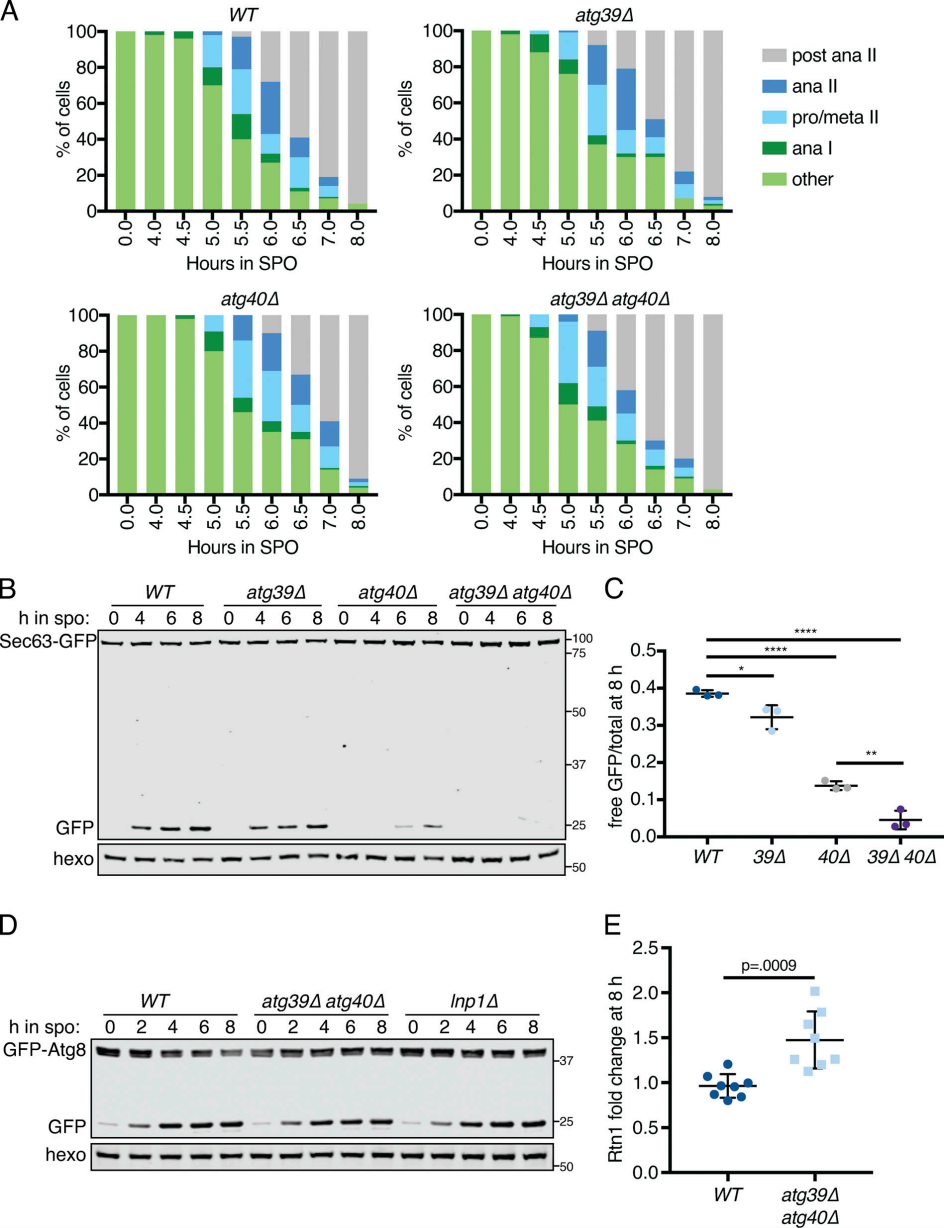
**Developmentally regulated Atg40 expression drives selective ERphagy in meiosis.** **(A)** Quantification of meiotic staging for cells of the indicated genotypes. ana, anaphase; meta, metaphase; pro, prophase. **(B)** Western blot using samples from cells of the indicated genotypes expressing Sec63-GFP taken at the indicated times in meiosis. Blots were probed for GFP and hexokinase. **(C)** Average and SD quantifying free GFP as a proportion of the total GFP signal at 8 h from *n* = 3 replicates of the experiment in B. P values calculated using Student’s *t* test. *, P < 0.05; **, P < 0.01; ****, P < 0.0001. **(D)** Western blot using samples from cells of the indicated genotypes expressing GFP-Atg8 taken at the indicated times in meiosis and probed for GFP and hexokinase. **(E)** Average and SD quantifying Rtn1-GFP abundance normalized to hexokinase at 8 h in meiosis using *n* = 8 replicates from the experiment in Fig. 6 A. P value calculated using Student’s *t* test. Source data are available for this figure: SourceData FS6.

Autophagy can occur either selectively or nonselectively. In selective autophagy, cargo-specific autophagy receptors recruit autophagosomes to their cargo (Anding and Baehrecke 2017). Two budding yeast proteins, Atg39 and Atg40, have been identified as ER-localized autophagy receptors (Mochida et al., 2015). During nitrogen starvation and rapamycin treatment, Atg39 mediates the autophagic degradation of the perinuclear ER and some nuclear material, whereas Atg40 promotes autophagy of the cortical ER. The developmental specificity of ERphagy induction suggests that cells exert some degree of selectivity in defining meiotic autophagy targets. To further determine if ERphagy in meiosis takes place selectively, we examined cells lacking either or both ERphagy receptors. Cells lacking Atg40 showed normal meiotic progression, but autophagy of the cortical ER marker Rtn1-GFP was significantly reduced (Fig. 6, A–C; and Fig. S6 A). Cells lacking both Atg39 and Atg40 demonstrated a more severe defect in Rtn1 autophagy than those lacking Atg40 alone (Fig. 6, A and B), suggesting that Atg39 is capable of mediating autophagy of cortical ER markers in the absence of Atg40. Both Atg39 and Atg40 were important for autophagy of Sec63, a member of the translocon complex that localizes throughout the cortical and perinuclear ER (Fig. S6, B and C). Consistent with previous work, we found that Atg39 and Atg40 function specifically in the ERphagy pathway in meiosis, as *atg39Δ atg40Δ* cells show normal levels of general autophagy (Fig. S6 D; Mochida et al., 2015). Thus cells undergoing meiosis selectively target the ER for degradation via Atg39- and Atg40-mediated autophagy.

### Atg40 expression is a developmental cue that triggers ERphagy

ERphagy occurs primarily in late meiosis, downstream of the transcription factor Ndt80, but the precise developmental cues promoting ERphagy are still unclear (Fig. 5, E and F). We examined Atg40 abundance during meiosis to determine if autophagy receptor expression itself might be the trigger for ER degradation. Atg40 was lowly expressed in vegetative cells and in early meiosis but was strongly induced in mid- to late meiosis, peaking in expression at ∼6 h before gradually declining (Fig. 6, D and E). This spike in Atg40 levels occurred during ER collapse and immediately preceded the autophagic degradation of Rtn1, indicating that developmentally regulated autophagy receptor expression drives ERphagy (Fig. 6, D and E; and Video 22). ERphagy precedes prospore membrane closure and meiotic ER inheritance into spores (Fig. 6 E and Video 22) and occurs normally in cells lacking prospore membranes (*spo21Δ;* Fig. S5 I), supporting a model by which ERphagy removes a portion of ER from eligibility for inheritance.

**Video 22.** **Time-lapse epifluorescence microscopy of the meiotic budding yeast cell depicted in ****Fig. 6 E****.** Atg40-3xGFP is shown in green and mKate-Spo20^51–91^ in magenta. Cell is imaged over 18 h with images collected every 20 min. Video shows 5 frames per second.

Do other cues feed into ERphagy induction, or is autophagy receptor expression the principal regulatory cue? We noted that cells arrested before meiotic entry (*ime1Δ*) or in prophase I (*ndt80Δ*) show low Atg40 expression and exhibit very little ERphagy (Fig. 5 E). To determine if providing cells with Atg40 in this context would be sufficient to induce ERphagy, we constructed a conditional allele of Atg40 using the copper-inducible *CUP1* promoter (*pCUP1-ATG40*). If Atg40 production is the limiting regulatory step for meiotic ERphagy, arrested cells should show Rtn1 degradation upon Atg40 induction. If additional developmental cues are required, Atg40 expression should be insufficient to trigger Rtn1 degradation. Consistent with the former model, copper-induced Atg40 expression resulted in robust autophagic degradation of Rtn1 during premeiotic arrest (Fig. 6 F). In contrast, Atg40 overexpression in mitotic cells grown in rich media did not result in enhanced Rtn1 degradation. These results indicate that cells in SPO are primed for autophagy, and that the developmentally regulated expression of a single autophagy receptor is necessary and sufficient to trigger cargo degradation in this context. To assess the role of ERphagy in successful gamete production, we measured spore formation in cells lacking one or both ERphagy receptors. We found that loss of Atg40 resulted in decreased sporulation efficiency, which was further exacerbated in the *atg39Δ atg40Δ* double mutants (Fig. 6 G). These results suggest that ERphagy is dispensable for meiotic chromosome segregation (Fig. S6 A) but is activated in mid- to late meiosis to ensure proper gamete production.

### ER collapse promotes ERphagy

**Figure S7.**
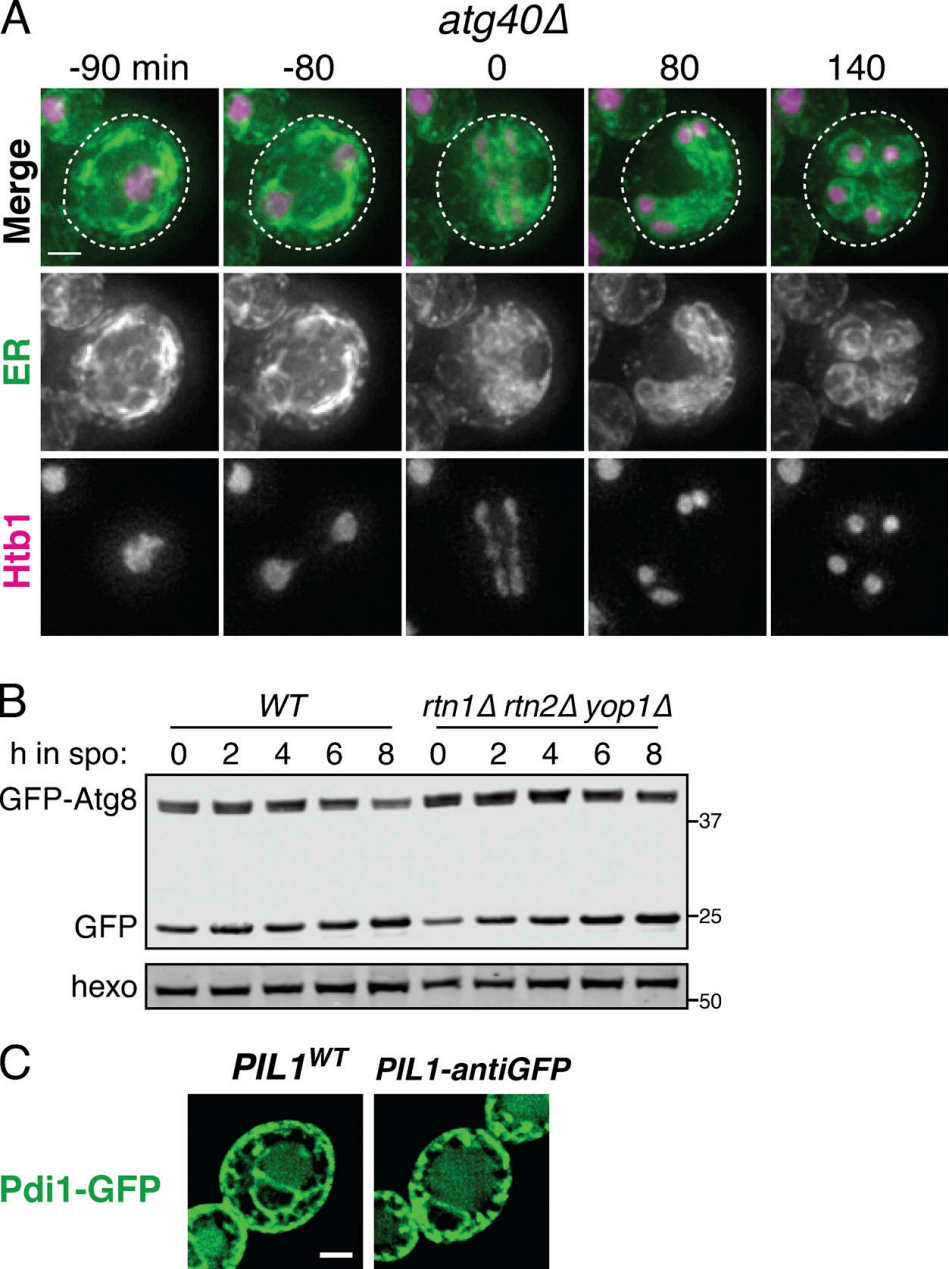
**ER collapse is not dependent on ERphagy, and reticulons do not affect global autophagy.** **(A)** Time-lapse microscopy of *atg40Δ* cells expressing GFP-HDEL (ER) and Htb1-mCherry imaged every 10 min during meiosis. 0 min is defined as the time of ER collapse. Scale bar = 2 µm. **(B)** Western blot with WT or *rtn1Δ rtn2Δ yop1Δ* cells expressing GFP-Atg8 taken at the indicated times in meiosis. Probed for GFP and hexokinase. **(C)** Fluorescence microscopy of cells expressing Pdi1-GFP and either untagged (WT) or nanobody-tagged (antiGFP) Pil1. Scale bar = 2 µm for all microscopy figures. Note that presence of Pil1-antiGFP does not affect Pdi1 cortical tethering, consistent with its lumenal localization preventing nanobody binding. Source data are available for this figure: SourceData FS7.

**Figure 7.**
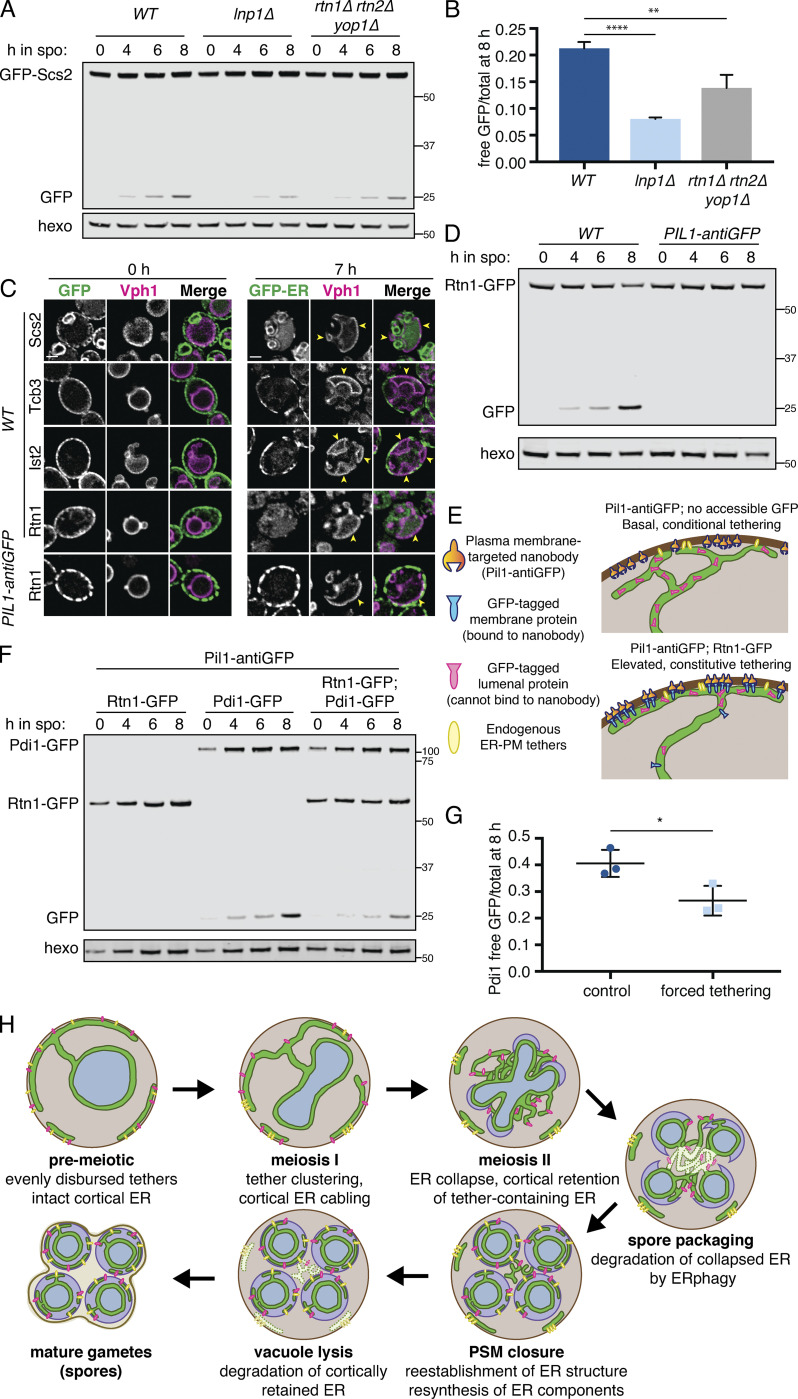
**ER collapse is required for ERphagy.** **(A)** Western blot with samples taken from cells of the indicated genotypes expressing GFP-Scs2 at the indicated times after transfer to SPO and probed for GFP and hexokinase. **(B)** Average and SD quantifying free GFP as a proportion of total GFP signal 8 h after transfer to SPO, using *n* = 3 replicates of the experiment from A. P values calculated by Student’s *t* test. **, P < 0.01; ****, P < 0.0001. **(C)** Microscopy images of cells expressing Vph1-mCherry and the indicated GFP-tagged ER protein and either an untagged (*WT*) or an anti-GFP nanobody–tagged allele of Pil1 (*PIL1-antiGFP*). Images were taken at 0 and 7 h following transfer to SPO. Scale bar = 2 µm. Arrowheads indicate pockets of vacuole. **(D)** Western blot with samples taken from cells expressing Rtn1-GFP and either WT Pil1 or Pil1-antiGFP. Samples were taken at the indicated times following transfer to SPO and probed for GFP and hexokinase. **(E)** Schematic outlining the experimental concept in F, in which a lumenal GFP-tagged protein is not accessible to Pil1-antiGFP binding and therefore does not affect tethering, but expression of an ER membrane protein with a cytosolically accessible GFP tag results in elevated tethering. **(F)** Western blot with samples taken from cells expressing Pil1-antiGFP and the indicated GFP-tagged proteins. Samples harvested at the indicated times following transfer to SPO and probed for GFP and hexokinase. **(G)** Average and SD quantifying free GFP as a proportion of total GFP signal 8 h after transfer to SPO, using *n* = 3 replicates of the experiment from F. P values calculated by Student’s *t* test. *, P < 0.05. **(H)** Model for ER remodeling during the meiotic program in budding yeast. ER is represented in green, PSM in purple, retained tethers in yellow, and Scs2/22 in pink. Arrows indicate progression through meiosis. Source data are available for this figure: SourceData F7.

We noted that mutants with increased cortical ER retention in meiosis, namely *lnp1Δ* and *rtn1Δ rtn2Δ yop1Δ* cells, are also reported to be deficient in starvation-induced ERphagy (Chen et al., 2018). The converse is not true, as autophagy-deficient (*atg40Δ* or Atg1-depleted) cells display normal ER collapse (Figs. S5 E and S7 A). We confirmed that *lnp1Δ* and *rtn1Δ rtn2Δ yop1Δ* mutant cells showed reduced ERphagy (but not global autophagy) in meiosis (Fig. 7, A and B; and Figs. S6 D and S7 B). Although ER collapse does not take place in starvation-induced autophagy, Atg40 must translocate to the cell interior for ERphagy to occur efficiently (Chen et al., 2018), suggesting that small cortical ER domains targeted for autophagy must be able to detach from the PM in this context. We therefore wondered whether ER collapse is important for ERphagy in a meiotic context.

We reasoned that if collapsed ER is the pool that is targeted for ERphagy, cortically retained tethers should not be subject to ERphagy. Consistent with this model, we saw very little autophagic degradation of the cortically retained tethers GFP-Ist2 and Tcb3-GFP but robust autophagic degradation of GFP-Scs2, an ER-PM tether that collapses with the bulk cortical ER, and Rtn1-GFP, a non-tether control present in both collapsed and retained cortical ER populations (Fig. 7 C).

We further reasoned that if meiotic ERphagy depends on ER collapse, engineered targeting of a protein that is normally abundant in collapsed ER to the retained cortical ER compartment should prevent it from being subject to ERphagy. Indeed, ectopically targeting Rtn1-GFP to the cortically retained ER compartment using a Pil1-nanobody abolished autophagic degradation of Rtn1-GFP, supporting the model that collapsed ER is robustly targeted by autophagy while cortically retained ER is not (Fig. 7, C and D). To exclude the possibility that ERphagy of Rtn1-GFP was blocked in this experiment due to its interaction with the Pil1-nanobody rather than as a result of its cortical retention, we performed a similar experiment in cells also expressing GFP-tagged Pdi1, an abundant protein that localizes to the ER lumen and therefore cannot bind to the Pil1 nanobody (Figs. 7 E and S7 C). Pdi1-GFP ERphagy was indeed reduced in these cells, consistent with enhanced cortical ER retention resulting in less collapsed ER eligible for ERphagy (Fig. 7, F and G). Thus, the dramatic morphological changes in the ER during meiosis are integrally linked to its regulated degradation.

## Discussion

Here, we investigated the extensive ER remodeling that is programmed into the natural developmental process of meiosis and gamete formation in budding yeast (Fig. 7 H). This remodeling occurs in a stepwise manner, beginning early in meiosis with cortical ER cabling and ER-PM tether clustering. Next, coincident with anaphase II, the cortical ER undergoes reticulon- and Lnp1-dependent collapse from the PM, while a subset of ER islands containing ER-PM tethers remain at the cell cortex. Collapsed ER is then subject to Atg40-dependent selective autophagy, whereas cortically retained ER is degraded by vacuolar lysis following spore closure. Together, these findings reveal that developmentally regulated ER compartmentalization, membrane tethering, and two parallel pathways for ER degradation combine to mediate ER inheritance by gametes while selectively eliminating a subset of ER.

An especially unexpected finding presented here is the meiotically regulated transition of cortical ER from the well-defined PM-proximal network to physically separated ER subcompartments, with the fate of each ER region determined by whether or not it contains ER-PM tethers. Cortical ER that is free of the Tcb and Ist2 tethers is subject to ER collapse, whereas tether-containing regions remain at the PM and are excluded from gametes. Consistent with this model, artificially tethering the abundant cortical ER protein Rtn1 to the PM increases cortical ER retention but does not prevent ER collapse. Although programmed cortical ER separation has not, to our knowledge, been described during differentiation, it elegantly achieves two distinct outcomes at once, allowing bulk cortical ER inheritance while partitioning specific regions of ER away from gametes.

A major unanswered question is how reticulons and Yop1 mediate cortical ER separation. We favor the model that these proteins drive membrane fragmentation, generating untethered fragments that dissociate from the PM and tethered fragments that remain cortically associated, although we cannot rule out an indirect role for membrane curvature in this process. A large body of evidence suggests that increased reticulon concentration drives membrane fission in various contexts. In some cases, fragmentation occurs globally upon reticulon overexpression (Espadas et al., 2019; Wang et al., 2016), whereas in others, fragmentation relies on a high local reticulon concentration achieved through multivalent interactions or homo-oligomerization (Grumati et al., 2017; Bhaskara et al., 2019; Jiang et al., 2020; Mochida et al., 2020). In at least one case, phosphorylation of a reticulon-like protein has been shown to promote oligomerization-dependent fragmentation (Jiang et al., 2020). Several lines of reasoning lead us to favor a regulated oligomerization model over increased reticulon expression as the mechanism driving meiotic ER fragmentation. First, new protein synthesis on the scale required to drive fragmentation is slow and energetically costly relative to the regulatory mechanisms that might control oligomerization, such as phosphorylation. Second, global measurements of protein synthesis and abundance suggest that reticulon levels do not appreciably increase during the time leading up to ER collapse (Brar et al., 2012; Cheng et al., 2018). Third, fragmentation purely as a result of reticulon abundance has only been observed in vitro or using overexpression systems in which reticulons exceed physiological levels. In contrast, regulated reticulon oligomerization is deployed by cells in response to specific challenges (Jiang et al., 2020). Our finding that reticulons and Yop1 are involved in meiotic ER collapse and inheritance further highlights that these proteins are more than inert structural proteins and instead can serve critical roles in dynamic ER remodeling. It will be important to determine the developmental cues regulating reticulon-dependent membrane scission in meiosis.

Morphological homeostasis of the tubular ER network requires a balance between membrane fusion and fission. We propose that this balance shifts toward fission in meiotic cells, resulting in fragmented ER. Consistent with a need for reduced tubule fusion in this process, we found that Lnp1, which has been suggested as a negative regulator of Sey1-mediated tubule fusion (Chen et al., 2012), is important for ER collapse in meiosis. Cells lacking Lnp1 formed massive ER foci that remained attached to the cell cortex throughout meiosis. How these structures form and their precise composition are still unclear, although the decrease in focus size and abundance in *lnp1Δ sey1Δ* double mutant cells suggests that they are the result of excessive membrane fusion in the absence of Lnp1. These structures are not present in *lnp1Δ* cells undergoing exponential mitotic growth, although we did observe them in saturated *lnp1Δ* cultures (data not shown), suggesting an unexpected role for Lnp1-dependent ER remodeling during nutrient adaptation. Further elucidation of the function of Lnp1 in these conditions would provide crucial insight into the role of this conserved yet poorly understood family of proteins.

We found that four of the six defined ER-PM tethers, Ist2 and the three tricalbins, mark cortically retained ER and are important for the physical exclusion of these ER islands from gametes. Loss of these four retained tethers reduces the amount of cortically retained ER at anaphase II but does not completely prevent ER retention, suggesting that additional tethers and/or alternative mechanisms ensure cortical ER tethering in this context. In addition to the six defined tethers, other proteins have been observed to localize to ER-PM contact sites, and some have been proposed as active ER-PM tethering proteins (Petkovic et al., 2014; Topolska et al., 2020). Study of these additional factors will be important to further interrogate the molecular basis of cortical ER retention during meiosis and its role in ensuring gamete health.

What accounts for the difference in behavior between the four cortically retained tethers and Scs2/22? An intriguing distinction between the two sets of proteins is their mode of PM association. Ist2, Tcb1, Tcb2, and Tcb3 associate with the PM through interactions with lipids, whereas the VAP orthologues, Scs2 and Scs22, associate with integral PM proteins containing FFAT (two phenylalanines in an acidic tract) or FFAT-like motifs (Manford et al., 2012; Murphy and Levine, 2016). This suggests a different mode of PM release for the two classes of ER-PM tethers, with release of Scs2 and Scs22 potentially occurring through modification or destruction of their protein partners. ER-PM tethers were also recently revealed to localize to distinct membrane subdomains based on differential affinity for membrane curvature, with tricalbins enriched on curved membranes and Scs2/Scs22 preferring flat membranes (Collado et al., 2019; Hoffmann et al., 2019). The basis for ER-PM detachment or retention and the potential role for accessory proteins and/or membrane curvature is an interesting area of future study.

What is the purpose of excluding certain parts of the ER from inheritance by gametes? One possibility is that this process serves as a form of ER quality control, selectively preventing the inheritance of damaged or toxic ER or ER-associated material. This may be akin to a recently described mechanism by which various types of age-induced damage (including protein aggregates, extrachromosomal rDNA circles, and expanded nucleolar material) are targeted to a compartment that is formed from the nuclear envelope as a means of their exclusion from gametes (King et al., 2019). Targeting deleterious ER contents to the cortically retained compartment would be an efficient means of ensuring their physical exclusion from gametes. An important challenge with such a system would be achieving specificity in what is targeted to cortically retained ER. Thus far, the only proteins that we identified to preferentially localize to the cortically retained compartments are ER-PM tethers themselves, whereas GFP-HDEL, reticulons, Sec63, Lnp1, and Sey1 are seen in both cortical and collapsed ER populations (Figs. 1 A and 4 D and Fig. S3, F and J). It will be important to determine in future studies whether other ER proteins or cellular components selectively localize to this compartment and the role that ER-PM tethers play in this process.

In addition to the exclusion of cortically retained ER from gametes, we identified programmed selective autophagy as a parallel and mutually exclusive means of eliminating ER subdomains during meiosis. Although ERphagy has primarily been studied in the context of nutrient limitation or ER stress in the presence of harsh chemical treatment, our identification of its natural role in meiosis provides an opportunity to study its endogenous regulation in a developmental context. We identified the timed expression of the ER-specific autophagy receptor Atg40 as a key developmental cue regulating ERphagy in meiosis. Atg40 expression depends on the meiotic transcription factor Ndt80, but whether Ndt80 promotes Atg40 expression directly remains to be determined. Previous work has shown that components of the histone deacetylase complex Rpd3L repress *ATG40* transcription in nutrient-replete conditions (Cui et al., 2019), suggesting that inhibition of this complex may allow meiotic Atg40 expression. Our findings warrant a more detailed study of how its expression and activity are regulated and the role of ERphagy in the broader developmental context of meiosis.

As with cortical ER retention, it is appealing to hypothesize that ERphagy serves as a meiotic quality control mechanism. The autophagy receptors Atg39 and Atg40 are selective for perinuclear and cortical ER, respectively, during starvation-induced and meiotic ERphagy. However, whether there is additional specificity to what ER content is degraded in any context is yet to be determined. ERphagy receptors in mammalian cells have been shown to preferentially degrade subsets of the ER proteome, including misfolded proinsulin and procollagen aggregates, but the molecular basis for this specificity is largely unknown (Forrester et al., 2019; An et al., 2019; Cunningham et al., 2019). In the future, understanding how cells precisely target cargo for degradation by ERphagy to remodel the ER and mitigate ER stress will be crucial for defining the role of ERphagy in meiosis. Moreover, owing to the conservation of ERphagy factors in mammals, it is possible that the natural role of ERphagy in meiotic development suggests a more widespread role for this process in mammalian cellular development programs.

The most widely studied substrates for ER quality control are misfolded proteins, which are induced by genetic or chemical disruption of ER protein folding capacity or through exogenous expression of model aggregate-prone proteins. During mitosis in budding yeast, misfolded ER proteins are retained in mother cells, promoting daughter cell rejuvenation at the expense of reduced mother cell lifespan (Clay et al., 2014; Piña and Niwa 2015). Intriguingly, while the vast majority of ER components enter into the daughter cell during mitotic ER inheritance in unstressed conditions, all four cortically retained tethers are tightly restricted to the mother cell (Takizawa et al., 2000; Okada et al., 2017; Sugiyama and Tanaka 2019). Moreover, asymmetric inheritance of both misfolded proteins and ER-PM tethers relies on an ER membrane diffusion barrier established by septin ring components at the bud neck (Clay et al., 2014; Sugiyama and Tanaka, 2019), suggesting a shared mechanism to control the selective inheritance of ER protein aggregates and ER-PM tethers and raising the possibility that ER-PM tethers themselves may mark or actively participate in the age-dependent accumulation of ER stress. A conserved feature of gametogenesis is the elimination of age-induced damage to produce healthy, youthful gametes (Goodman et al., 2020; Ünal et al., 2011; King et al., 2019). While naturally occurring markers of age-induced ER damage have not been described in yeast, our findings warrant further study of relationship between aging, ER stress, and ER inheritance during meiosis and mitosis.

## Materials and methods

### Yeast strains, plasmids, and primers

All experimental strains are diploid *Saccharomyces cerevisiae* derivatives of the SK1 strain as detailed in Table S1. The following alleles were derived from previous studies: *pGAL-NDT80* and *GAL4.ER* (Benjamin et al., 2003; Carlile and Amon 2008), *pCLB2-CDC20* (Lee and Amon, 2003), *pCUP1-osTIR* (Sawyer et al., 2019), *mKate-SPO20^51–91^* and *VPH1-mCherry* (King et al., 2019), *HTB1-mCherry* (Matos et al., 2008), *ndt80Δ* (Xu et al., 1995), and *GFP-ATG8* (Graef et al., 2013). The *atg8::LEU2* allele was a gift from Hilla Weidberg and Angelika Amon (Massachusetts Institute of Technology, Cambridge, MA).

Deletion and C-terminal tagging of genes at their endogenous loci were performed using the standard PCR-based technique (Longtine et al., 1998; Janke et al., 2004) with the PCR product of the primers indicated in Table S2 and plasmids indicated in Table S3. The primers used to GFP tag Sey1 were 5′-CAA​AAA​AGT​AAA​CAC​GGT​AAA​TTG​AAA​TAA​ATT​ATT​CGA​TTC​GAT​GAA​TTC​GAG​CTC​G-3′ and 5′-TTT​ATA​TTT​TAC​ACA​GTG​CAT​CTT​GTC​CCT​CTC​TTT​CTC​GTC​GAT​GAA​TTC​GAG​CTC​G-3′. The primers used to GFP tag Tcb1 were 5′-TAA​GAA​GAA​TCA​TGA​GAT​GGG​CGA​AGA​AGA​AAC​TAA​GTT​TGG​TGA​CGG​TGC​TGG​TTT​A-3′ and 5′-TTT​ATA​TTT​TAC​ACA​GTG​CAT​CTT​GTC​CCT​CTC​TTT​CTC​GTC​GAT​GAA​TTC​GAG​CTC​G-3′. The primers used to tag Tcb2 were 5′-GTC​TAC​AAC​CAC​TGG​GGA​CAA​AAA​ATC​CGA​AGA​GAA​GCA​AGG​TGA​CGG​TGC​TGG​TTT​A-3′ and 5′-AAG​ATG​ATT​TCG​TGA​CAC​ATA​CTC​TTT​ACC​ATC​GAT​AGA​ATC​GAT​GAA​TTC​GAG​CTC​G-3′. The primers used to tag Tcb3 were 5′-GGT​ACC​TCC​CGT​GCC​AGA​AGT​TCC​TCA​AGA​ATA​CAC​GCA​GGG​TGA​CGG​TGC​TGG​TTT​A-3′ and 5′-AAC​AAA​CAC​AGA​AAA​GAC​ACC​TGT​TAA​CAC​ACC​AAA​TGT​GTC​GAT​GAA​TTC​GAG​CTC​G-3′. The primers used to tag Atg1 were 5′-CAG​GTT​GAA​AAT​ATT​GAG​GCA​GAA​GAT​GAA​CCA​CCA​AAA​TCG​GAT​CCC​CGG​GTT​AAT​TAA-3′ and 5′-GGT​CAT​TTG​TAC​TTA​ATA​AGA​AAA​CCA​TAT​TAT​GCA​TCA​CGA​ATT​CGA​GCT​CGT​TTA​AAC-3′. The primers used to tag Atg40 were 5′-TTT​TAT​GGA​GGA​TAT​TCT​AGA​TGA​GAC​AAC​TGA​ATT​GGA​TCG​GAT​CCC​CGG​GTT​AAT​TAA-3′ and 5′-CCT​TCA​TAG​ACT​ACC​ATT​ATG​GTA​AAA​TGG​AAA​AAC​TAT​TGA​ATT​CGA​GCT​CGT​TTA​AAC-3′. The primers used to tag Pdi1 were 5′-TGA​CGC​TGA​CGC​TGA​ATT​GGC​TGA​CGA​AGA​AGA​TGC​CAT​TGG​TGA​CGG​TGC​TGG​TTT​A-3′ and 5′-TTA​TAT​ATC​TCT​ATT​TAA​TGA​AAA​ACC​AAA​GTG​ATC​AGA​ATC​GAT​GAA​TTC​GAG​CTC​G-3′. The primers used to tag Lnp1 were 5′-ACC​GGC​ACA​GCC​TTC​GCA​GTG​GGA​AAA​GGA​AAA​AAC​AAA​AGG​TGA​CGG​TGC​TGG​TTT​A-3′ and 5′-TAA​AAA​TAT​ATT​ATA​TAG​GGG​TAC​GTA​GTT​ATT​CTA​ACG​CTC​GAT​GAA​TTC​GAG​CTC​G-3′. *GFP-SCS2* and *GFP-IST2* were created using a Cas9-based method similar to that in Sawyer et al. (2019). The gRNA sequences detailed in Table S2 (*SCS2*: 5′-GAC​TAG​CAG​ACA​TAC​TTA​GGT​TCG-3′ and 5′-AAA​CCG​AAC​CTA​AGT​ATG​TCT​GCT-3′; *IST2*: 5′-GAC​TAA​GAT​TAA​AAC​AAG​CAC​AAG-3′ and 5′-AAA​CCT​TGT​GCT​TGT​TTT​AAT​CTT-3′) were inserted into a centromeric plasmid carrying a *URA3* marker and *pPGK1-dCas9* to generate pÜB1834 and pÜB1978, respectively. These plasmids and repair templates containing GFP and a linker sequence were amplified from pÜB1548 using the oligonucleotides in Table S2 (*SCS2* template: 5′-TAA​TAG​TGT​AGC​AGA​AGT​GTA​TTC​TAC​AAT​CTG​CGC​GAA​CCT​AAG​TAT​GGG​TGA​CGG​TGC-3′ and 5′-TAT​ACA​CCA​ACA​CGT​CAG​GCG​AAA​TTT​CAA​CAG​CAG​ACA​TTG​GAT​CCA​CTA​GTT​CTA​GAG-3′; *IST2* template: 5′-TGG​ATC​CAC​TAG​TTC​TAG​AGC​GGC​CGC​TTG​TTT​GTA​CAA​TTC​ATC​CAT​ACC-3′ and 5′-TAA​CAC​AAT​TCG​GAT​CTA​GAG​ATG​TAA​TTG​TCT​GCG​ACA​TTG​GAT​CCA​CTA​GTT​CTA​GAG-3′), and these were cotransformed into yeast. The plasmid was lost by streaking cells without selection. Successful tagging was confirmed by PCR and sequencing. The repair template containing GFP and a linker sequence was amplified from pÜB1548. GFP-Scs22 expressed from its endogenous locus was not detectable by microscopy in our strain background (not depicted), so we generated an allele that is highly expressed in meiosis (*pATG8-GFP-SCS22*). *SCS22* was amplified from SK1 genomic DNA and cloned into pÜB1548 by Gibson assembly (Gibson et al., 2009), replacing the *ATG8* ORF. The *SCS22* intron was removed following the Q5 Site-Directed Mutagenesis protocol (New England Biolabs) to generate pÜB1889. This plasmid was digested with PstI and transformed into WT SK1. To generate *pCUP1-ATG40-3V5*, *ATG40-3V5* was amplified from yeast harboring that allele and cloned together with the *CUP1* promoter into the pÜB217 plasmid by Gibson assembly. The resulting plasmid was amplified with the primers indicated in Table S2 (5′-TTT​TAT​GGA​GGA​TAT​TCT​AGA​TGA​GAC​AAC​TGA​ATT​GGA​TCG​GAT​CCC​CGG​GTT​AAT​TAA-3′ and 5′-CTT​CAT​AGA​CTA​CCA​TTA​TGG​TAA​AAT​GGA​AAA​ACT​ATT​CTC​GAT​GAA​TTC​GAG​CTC​G-3′) and transformed into a strain harboring *atg40::KanMX*, replacing the KanMX cassette to give *atg40::pCUP1-ATG40-3V5-HygB*. To construct a *GFP-HDEL* construct that is stably expressed throughout meiosis, the *GFP-HDEL* sequence (published in Rossanese et al. [2001]) was cloned into a *TRP1* integrating vector harboring the *ARO10* promoter, obtained from Leon Chan (University of California, Berkeley, Berkeley, CA). The resulting *pARO10-GFP-HDEL-TRP1* construct was used to generate *pARO10-mCherry-HDEL* by Gibson assembly. Both constructs were transformed into yeast following digestion with PmeI.

### Media and growth conditions

Prior to the induction of meiosis, cells were grown at RT for 20–24 h to a density of OD_600_ ≥10 in YPD (1% yeast extract, 2% peptone, 2% glucose, 22.4 mg/liter uracil, and 80 mg/liter tryptophan). Cultures were then diluted to OD_600_ = 0.25 in BYTA (1% yeast extract, 2% bacto tryptone, 1% potassium acetate, and 50 mM potassium phthalate) and grown for 16–18 h (OD_600_ ≥4.5) at 30°C. Cells were then pelleted, washed with sterile MilliQ water, and resuspended to OD_600_ = 1.9 in SPO (2% potassium acetate, 40 mg/liter adenine, 40 mg/liter uracil, 10 mg/liter histidine, 10 mg/liter leucine, and 10 mg/liter tryptophan, adjusted to pH 7.0 and supplemented with 0.02% raffinose). Cultures were allowed to shake at 30°C for the duration of the experiment. For each stage, culture volume was one tenth of the flask volume to ensure proper aeration.

For experiments conducted during vegetative growth, cells were grown in YPD for 16–18 h at 30°C (OD_600_ ≥10). Cultures were then back-diluted to OD_600_ = 0.02–0.05. For imaging experiments, cells were examined at a density of OD_600_ = 0.6–0.8.

For experiments using the *pCUP1-ATG40-3V5 allele*, CuSO_4_ was added to a final concentration of 50 µM at the indicated times. For *pGAL-NDT80* experiments, β-estradiol or an equivalent volume of 100% EtOH was added to a final concentration of 1 µM β-estradiol. For Atg1-AID experiments, 50 µM CuSO_4_ was added to induce expression of *pCUP1-osTIR* followed immediately by 500 µM auxin (Sigma-Aldrich).

### Live-cell imaging

Images were acquired using a DeltaVision Elite wide-field fluorescence microscope (GE Healthcare) and a PCO Edge scientific complementary metal–oxide–semiconductor camera, with softWoRx software and a 60× NA1.42 oil-immersion Plan Apochromat objective. GFP and RFP filter sets were used, with the setting information for acquiring each figure panel provided in Table S4. Live-cell imaging was performed exactly as described in King et al. (2019), except fresh SPO was used in place of conditioned SPO. In short, cells were imaged in an environmental chamber heated to 30°C, using either the CellASIC ONIX Microfluidic Platform or concanavalin A–coated glass-bottom 96-well plates. Cultures of meiotic cells in SPO were transferred to the microfluidic plates and loaded at 8 psi for 5 s. SPO was applied with a constant flow rate pressure of 2 psi for 15–20 h. With plates, cells were adhered to wells, and 100 µl of SPO was added to each well. Specific imaging conditions are noted in Table S4. All time-lapse experiments were performed using the CellASIC system (EMD Millipore) in Y04D or Y04E microfluidics plates, with the exception of the LatA experiments, for which we used glass-bottom 96-well plates (Corning). Images were deconvolved using softWoRx software (GE Healthcare) using 3D iterative constrained deconvolution with 15 iterations and enhanced ratio.

### Image representation

Microscopy images shown in Fig. 1, A, C, E, and F; Fig. 2, A, B, E, and G; Fig. 3, A, C, and E; Fig. 4, B–G; Fig. 6 E; Fig. S2, C–F and H; Fig. S3, B, C, E, F, and J; Fig. S5, B and E; and Fig. S7, A and C, are a maximum projection of the three central z slices collected. Images in Fig. 2 D; Fig. 5 B; Fig. 7 C; Fig. S2, A and B; Fig. S3 A; and Fig. S5 F are single z slices. Images in Figs. 3 D and S4 C are maximum projections of all z slices.

### Image quantification

For time-lapse microscopy, anaphase I was defined as the first frame in which an elongated nucleus was observed (if applicable) or the first frame at which two distinct nuclear masses were visible. Anaphase II was defined as the first frame at which two elongated nuclei were observed following anaphase I. ER cabling was defined as the first frame at which ER cables were visible. ER cables are bright, cortically localized ER structures that are thicker and more dynamic than premeiotic cortical ER. ER collapse was defined as an abrupt movement of cortical ER toward the center of cells. Prospore membrane nucleation was defined as the first frame at which mKate-Spo20^51–91^ signal was visible as distinct puncta in the center of cells rather than PM-localized. Prospore membrane closure was defined as the frame at which membrane structure transitioned from elongated to circular. Vacuole lysis was defined as the time at which signal became diffuse rather than membrane localized. Degradation of GFP-Ist2 and Tcb3-GFP was defined as the frame at which their signal disappeared from the cell cortex.

Qualitative cortical ER classification was performed at anaphase II according to the guidelines outlined in Fig. 2 D.

For Gini index calculation, the cell periphery was traced for the centermost z-slice using the program Fiji. Pixel intensity was calculated along the length of the trace, resulting in a finite number of measurements, *n*. These measurements were then ordered from smallest to largest and given an integer ranking *i* based on this order (i.e., for each value 1 ≤ *i* ≤ *n*, where the smallest number in the dataset has *i* = 1 and the highest number has *i* = *n*). Background was subtracted using average pixel intensity from a cell-free region of the image. The Gini index (*G*) was determined using the formula

$$G=\frac{2}{\bar{x}n^{2}}\overset{n}{\underset{i=1}{\sum }}i\left(x_{i}-\bar{x}\right),$$

where $\bar{x}$ is the average of all measurements and *x_i_* is the intensity value of ranking *i* in the dataset. For each cell (*n* = 10), Gini values were calculated for at least seven time points before ER collapse and six time points following ER collapse.

For analysis of foci in *lnp1Δ* cells, foci were counted manually for ≥100 cells per genotype. Focus size was measured using Fiji. Briefly, images were z-projected using maximum-intensity projection and converted to 8-bit, and the threshold was adjusted so that foci were clearly visible. Foci were detected automatically using the “analyze particles" function, resulting in measurements for ≥134 foci across >100 cells per genotype.

For measurements of Rtn1 and Htb1 levels in heterozygously tagged cells, images were maximally projected over the full imaging volume in Fiji. Tracing was performed for the whole cell (anaphase I and anaphase II time points) or for individual spores, and average pixel intensity for the traced area was calculated for both channels. Measurements for the cell shown in Fig. S4, C and D, were obtained from the first frame at which individual spores were easily distinguishable until spores became tightly packed and therefore had significantly overlapping signal (≥480 min). Bright spore and dim spore images in Fig. S4 E were taken from the last time point at which spores did not significantly overlap.

### Statistics

Statistical tests used for data analysis are defined in the figure legends. These include the χ^2^ test in Figs. 2 F and 3 B and Student’s *t* test in Fig. 2 H; Fig. 3, F and G; Fig. 5, D and F; Fig. 6, B and G; Fig. 7, B and G; Fig. S3, D and G–I; Fig. S4, A and E; Fig. S5 D; and Fig. S6, C and E. Data distribution for Student’s *t* test analyses were assumed to be normal, but this was not formally tested.

### Meiotic staging

Meiotic staging was performed scoring DAPI and tubulin morphology by fluorescent microscopy. Samples were fixed in 3.7% formaldehyde for 12–24 h at 4°C. Cells were then washed with 100 mM potassium phosphate, pH 6.4, once with sorbitol citrate (100 mM potassium phosphate, pH 7.5, and 1.2 M sorbitol), and digested in 200 µl sorbitol citrate, 20 µl glusulase (Perkin-Elmer), and 6 µl zymolase (10 mg/ml; MP Biomedicals) for 3 h at 30°C while rotating. Samples were pelleted at 900 rcf for 2 min, washed with 100 µl sorbitol citrate, pelleted again, and resuspended in 50 µl sorbitol citrate. Samples were then mounted on slides prepared with poly-L-lysine, submerged in 100% methanol at −20°C for 3 min, transferred to 100% acetone at −20°C for 10 s, and allowed to air dry. Samples were then incubated at RT for 1 h in primary anti-tubulin antibody (RRID:AB_325005, MCA78G, 1:200; Bio-Rad) in PBS-BSA (5 mM potassium phosphate, 15 mM NaCl, 1% BSA, and 0.1% sodium azide). Samples were then washed three times in PBS-BSA and incubated with preabsorbed FITC-conjugated secondary antibody (RRID:AB_2340652, 712–095-153, 6:200; Jackson ImmunoResearch Labs) for 1 h at RT. Samples were washed three times with PBS-BSA and mounted with VectaShield Antifade Mounting Medium with DAPI (Vector Labs).

### Western blotting

Protein samples were prepared by TCA treatment of cells. For meiotic samples, 1.8 ml culture was mixed with 200 µl of 50% TCA (5% final concentration) and incubated at 4°C for 12–24 h. For vegetative mitotic samples, 3.42 OD units of culture was spun down for 2 min at 3,000 rcf, washed in sterile MilliQ water, resuspended in 5% TCA, and incubated at 4°C for 12–24 h. All samples were precipitated for 5 min at 20,000 rcf and washed in 1 ml acetone. The acetone was aspirated, and samples were allowed to dry for ≥20 min. Pellets were resuspended by bead beating for 5 min in 100 µl Tris-EDTA buffer (10 mM Tris-HCL, 1 mM EDTA, pH 8) supplemented with 3 mM DTT and 1× protease inhibitors (Roche) with 100 µl acid-washed glass beads. 50 µl of 3× SDS loading buffer was added, and samples were incubated at 95°C for 5 min and spun down for 5 min at 20,000 rcf. 4 µl sample was loaded onto a Bis-Tris acrylamide gel, separated at 150 V for 50 min, and transferred to a nitrocellulose membrane using the TransBlot Turbo system (Bio-Rad). Blots were blocked and probed overnight at 4°C with one or more of the following antibodies: anti-hexokinase (RRID:AB_219918, 100-4159, 1:15,000; Rockland), anti-GFP JL8 (1:2,000; Clontech), and anti-V5 (RRID:AB_2556564, R960-25, 1:2,000; Thermo Fisher Scientific). Blots were washed in PBST and incubated for 2 h in IRDye secondary antibodies (RRID:AB_10956166, 926-68071, 1:20,000; LI-COR and RRID:AB_621847, 926-32212, 1:20,000; LI-COR). Blots were imaged and quantified using the Odyssey system (LI-COR).

### Online supplemental material

Fig. S1 shows the timing of ER cabling relative to anaphase I and II and the impact of meiotic progression on ER collapse. Fig. S2 shows the behavior of ER-PM tethers with respect to ER collapse and meiotic progression. Fig. S3 shows the effects of reticulons and Lnp1 on meiotic ER remodeling. Fig. S4 shows quantification of the experiments in Fig. 4 and also degradation of a subset of ER proteins followed by resynthesis in spores. Fig. S5 shows features of ERphagy during meiosis. Fig. S6 shows the impact of Atg40 on selective ERphagy in meiosis. Fig. S7 shows controls for experiments in Fig. 7, focused on the link between ERphagy and collapse. Table S1 describes the strains used in this study. Table S2 describes the primers used in this study. Table S3 describes the plasmids used in this study. Table S4 describes the settings used for microscopy experiments. Video 1 shows the cell depicted in Fig. 1 A. Video 2 shows the cell depicted in Fig. 1 C. Video 3 shows the cell depicted in Fig. 1 F. Video 4 shows the Tcb3-GFP cell depicted in Fig. 2 A. Video 5 shows the Tcb1-GFP cell depicted in Fig. 2 A. Video 6 shows the Tcb2-GFP cell depicted in Fig. 2 A. Video 7 shows the GFP-Ist2 cell depicted in Fig. 2 A. Video 8 shows the premeiotic cell depicted in Fig. S2A. Video 9 shows the sporulating cell depicted in Fig. S2A. Video 10 shows the cell depicted in Fig. 2 D. Video 11 shows the cell depicted in Fig. 2 G. Video 12 shows the cell depicted in Fig. 3 A. Video 13 shows the cell depicted in Fig. S3B. Video 14 shows the cell depicted in Fig. 3 C. Video 15 shows the cell depicted in Fig. 3 E. Video 16 shows the cell depicted in Fig. 4 B. Video 17 shows the cell depicted in Fig. 4 C. Video 18 shows the cell depicted in Fig. 4 D. Video 19 shows the cell depicted in Fig. 4 E. Video 20 shows the cell depicted in Fig. 4 F. Video 21 shows the cell depicted in Fig. 4 G. Video 22 shows the cell depicted in Fig. 6 E.

## Supplementary Material

Table S1 lists strains used in this study.

Table S2 lists primers used in this study.

Table S3 lists plasmids used in this study.

Table S4 shows the settings used for microscopy experiments.

SourceData F5 contains original blots for Fig. 5.

SourceData F6 contains original blots for Fig. 6.

SourceData F7 contains original blots for Fig. 7.

SourceData FS4 contains original blots for Fig. S4.

SourceData FS5 contains original blots for Fig. S5.

SourceData FS6 contains original blots for Fig. S6.

SourceData FS7 contains original blots for Fig. S7.
