# Supplementary figures and images for: Programmed cortical ER collapse drives selective ER degradation and inheritance in yeast meiosis
Source: J Cell Biol. 2021 Oct 18;220(12):e202108105. doi: 10.1083/jcb.202108105 (PMC8562846; doi:10.1083/jcb.202108105)

GFP

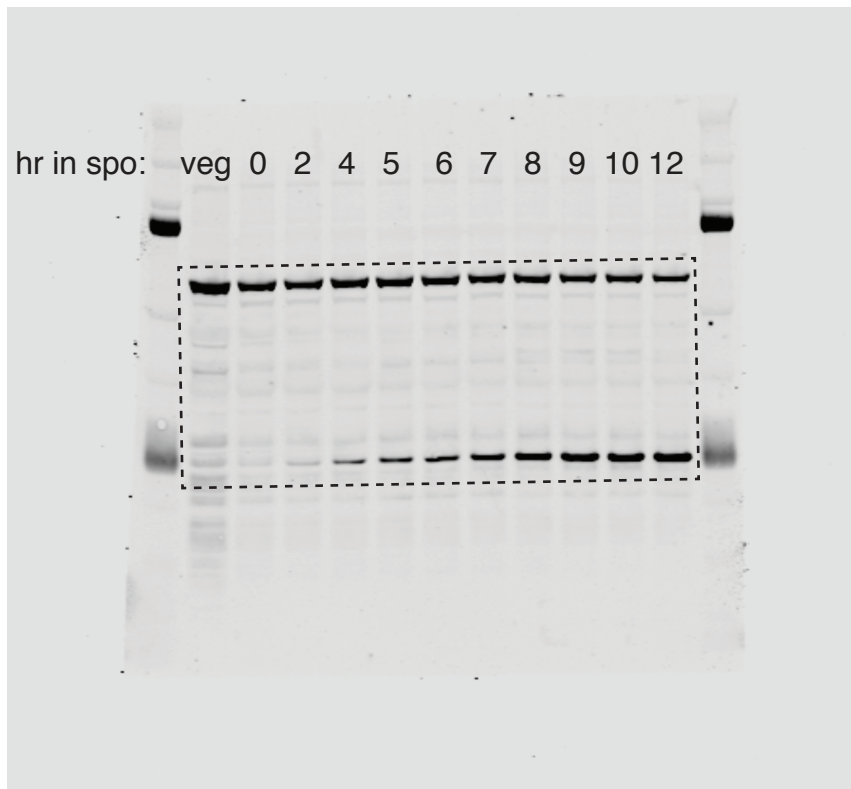

hexo

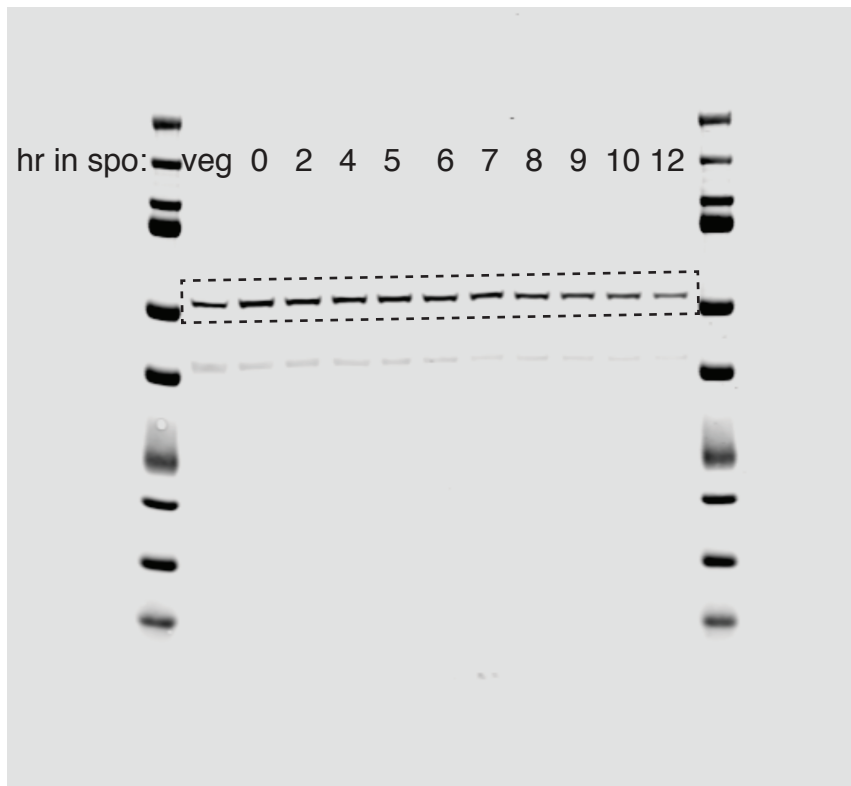

GFP

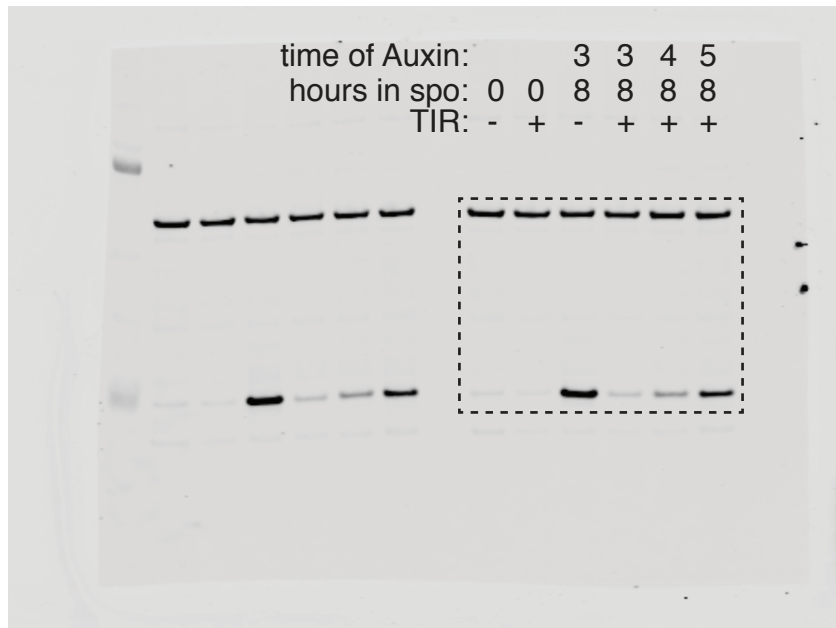

V5

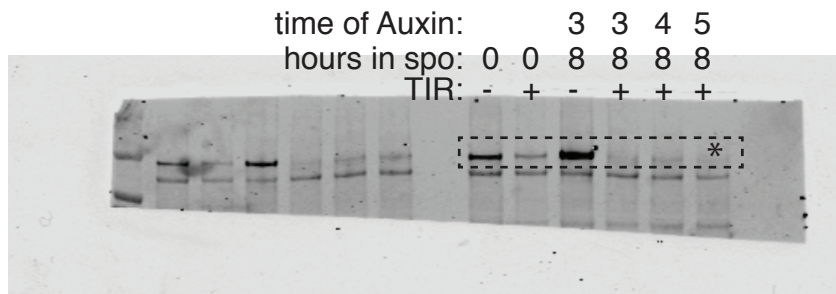

hexo

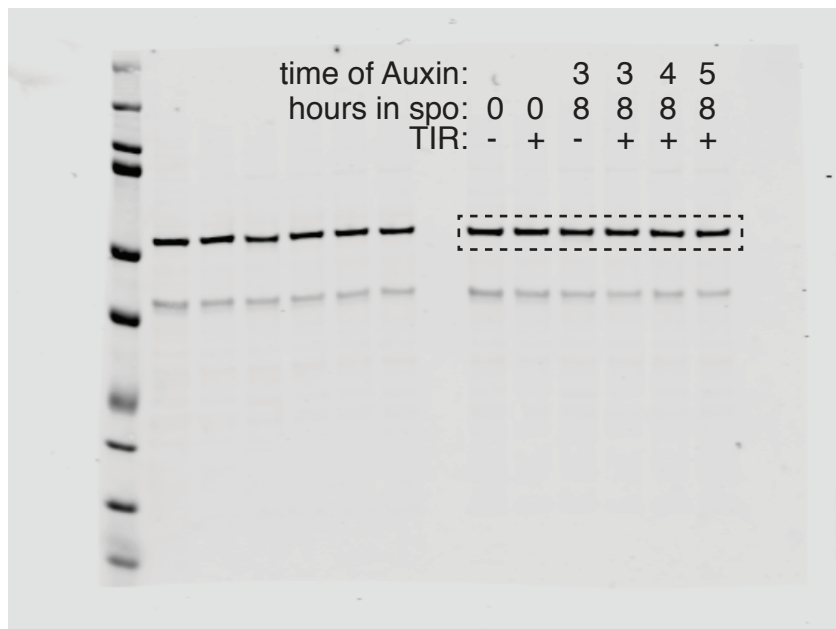

*GFP*

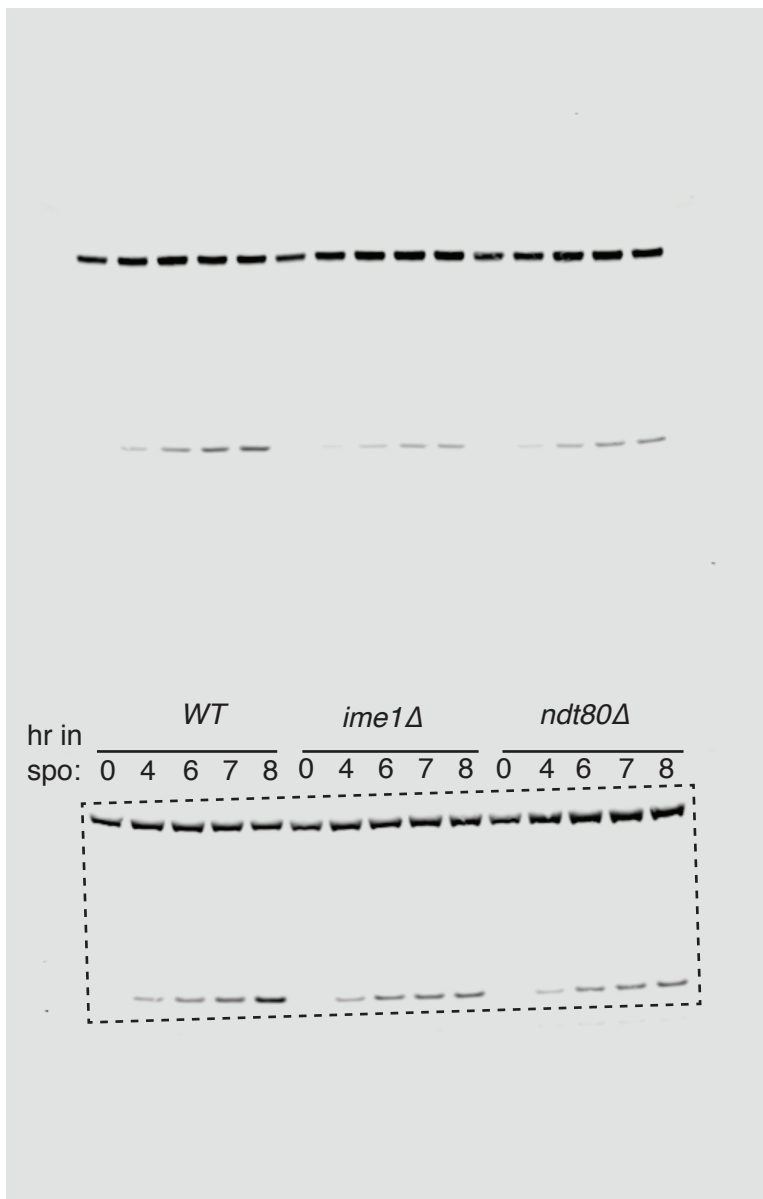

*hexo*

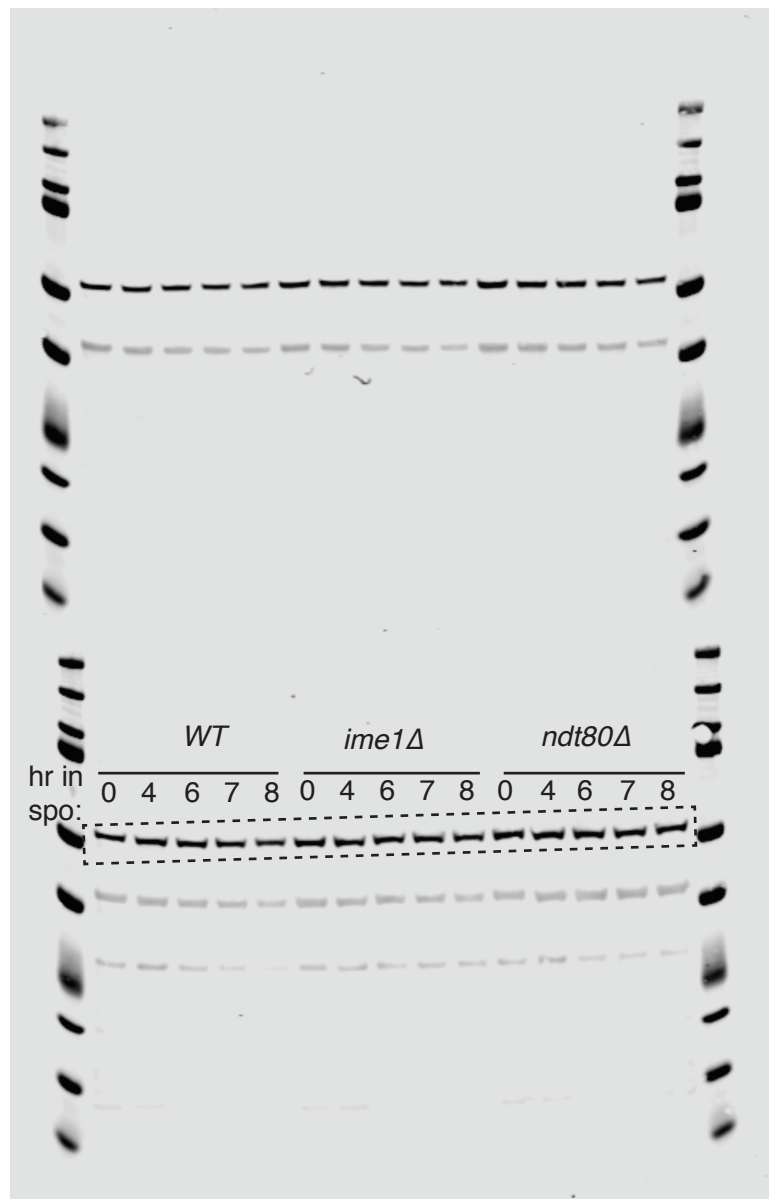

*V5*

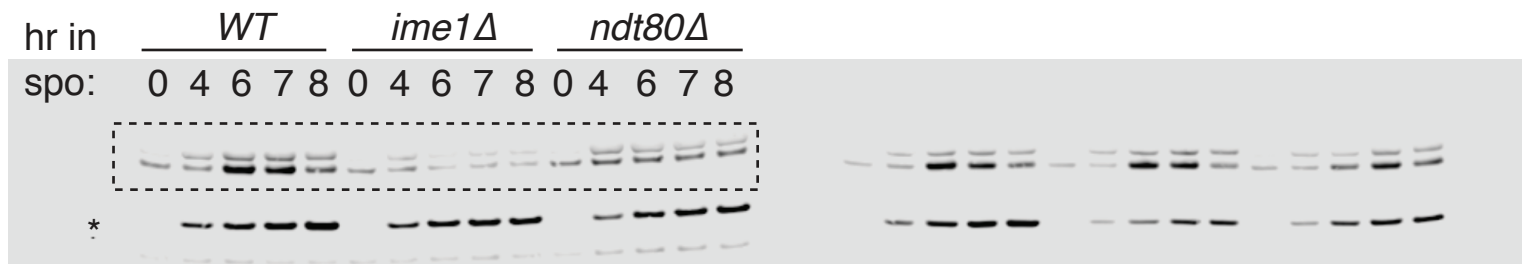

\* - free GFP from previous probing

Supplement: SourceData F5 — contains original blots for Fig. 5. [file JCB_202108105_SourceDataF5.pdf]

GFP

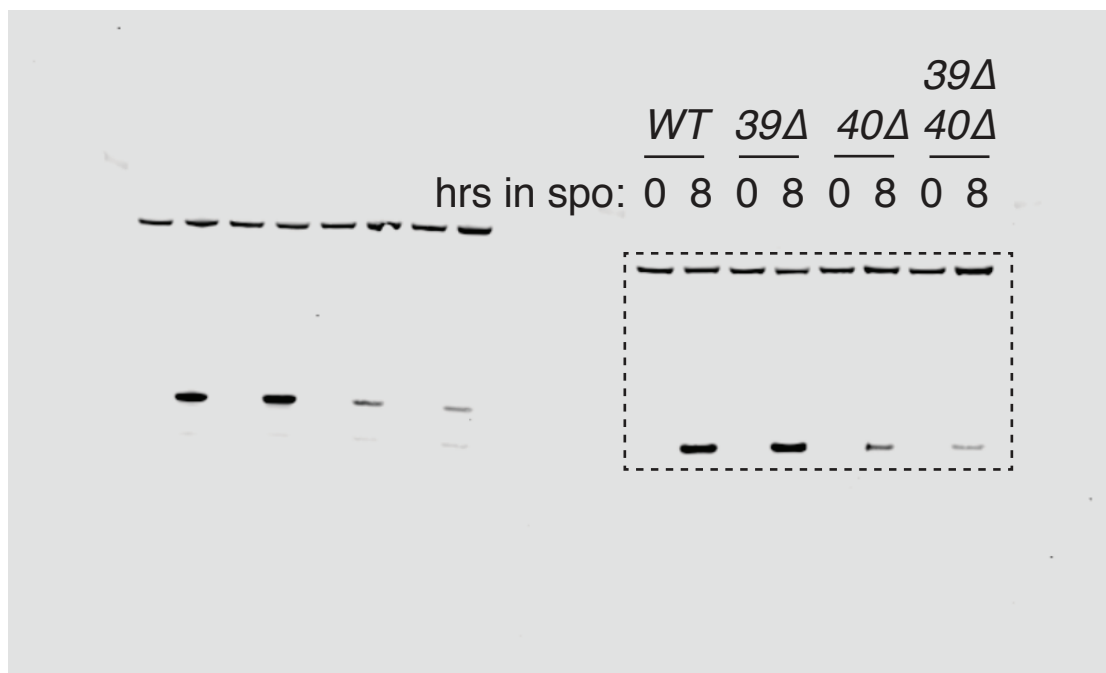

hexo

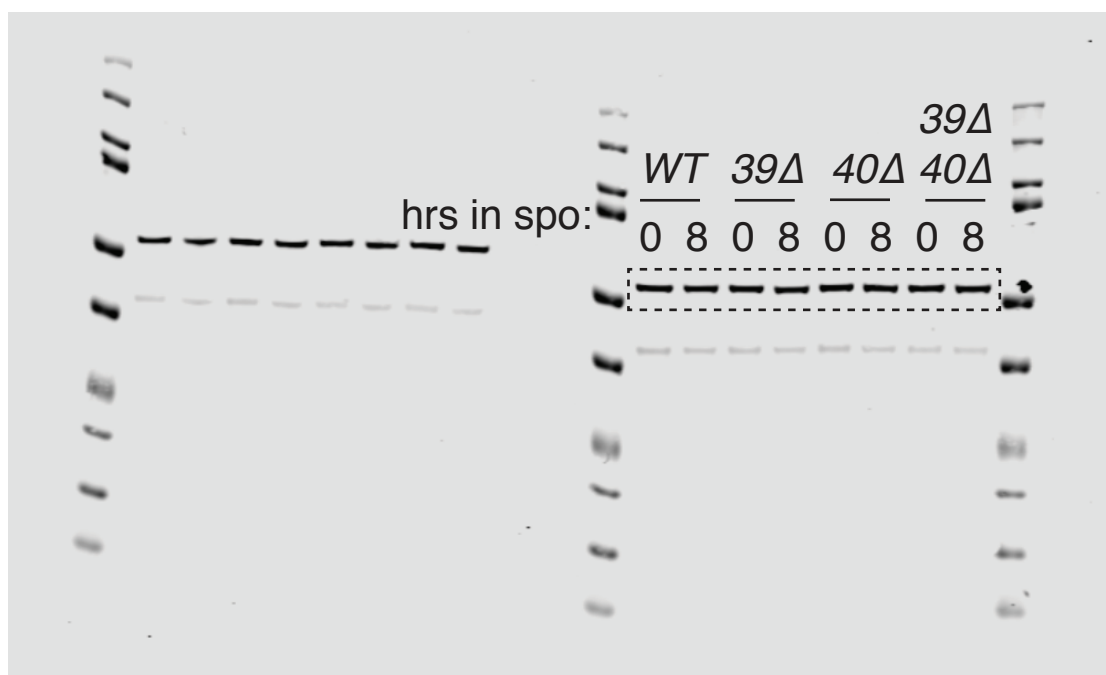

hrs in spo: 0 4 5 6 7 8 9 10

GFP and V5

hrs in spo: 0 4 5 6 7 8 9 10

hexo

GFP

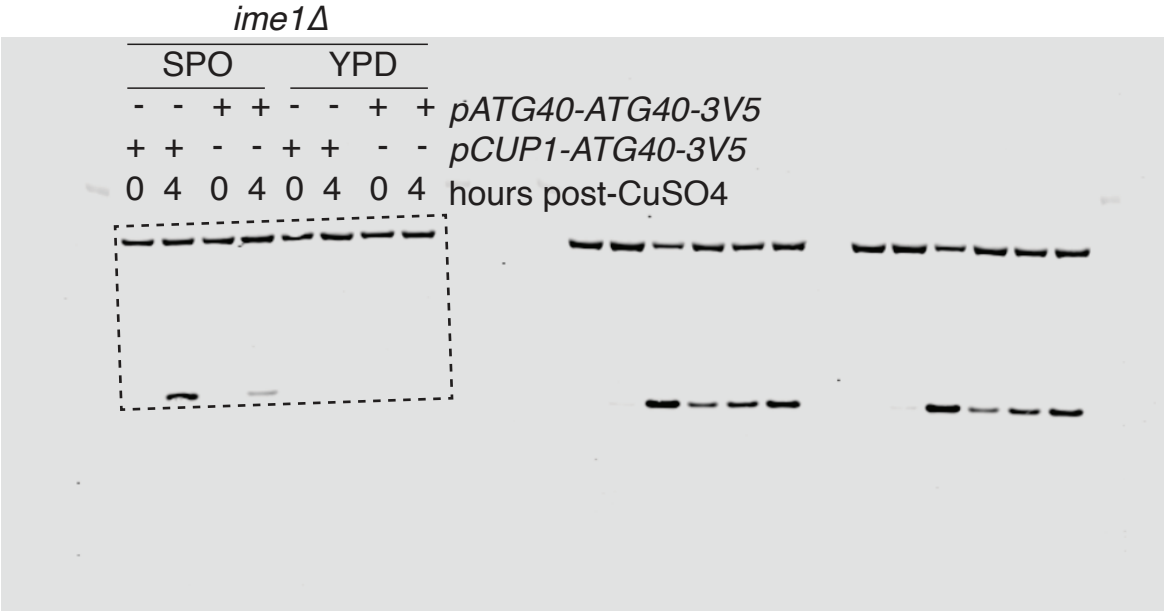

V5

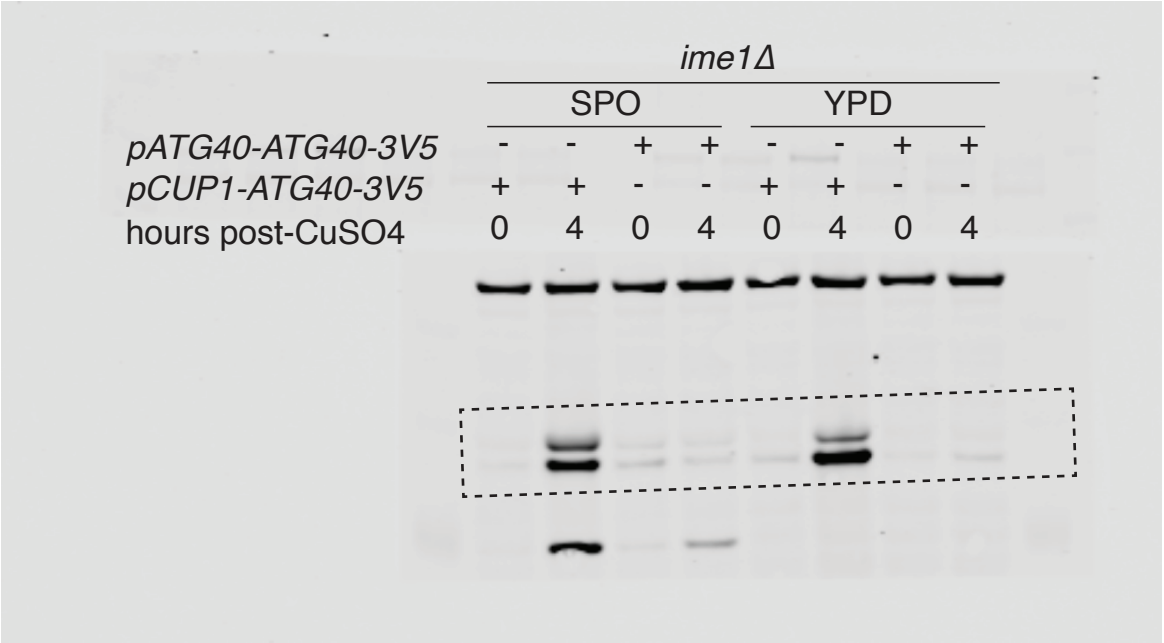

hexo

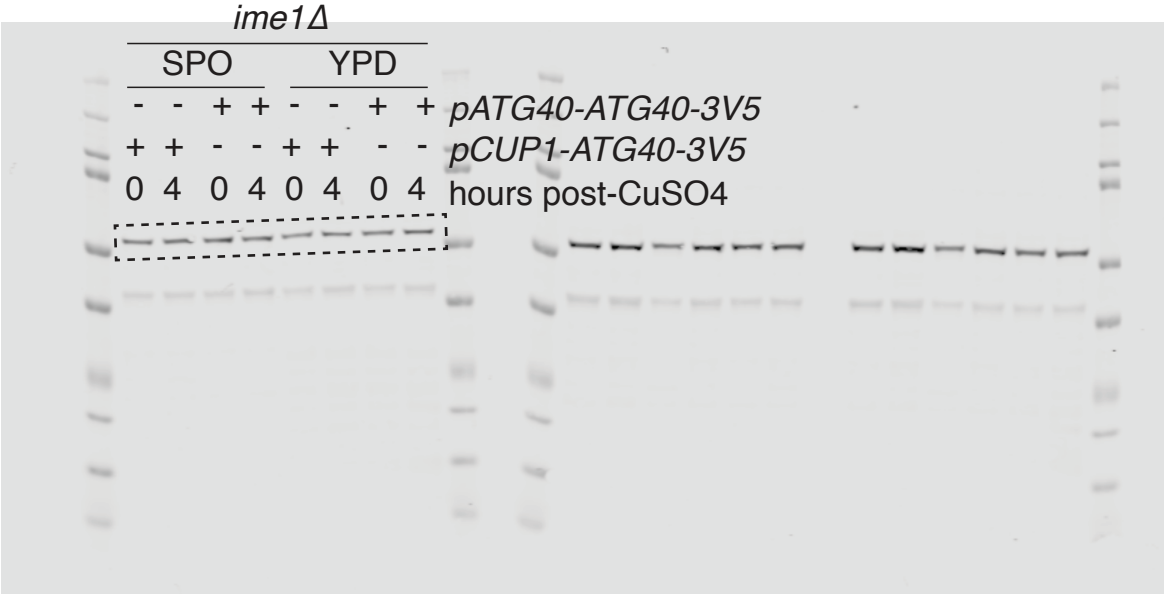

Supplement: SourceData F6 — contains original blots for Fig. 6. [file JCB_202108105_SourceDataF6.pdf]

GFP

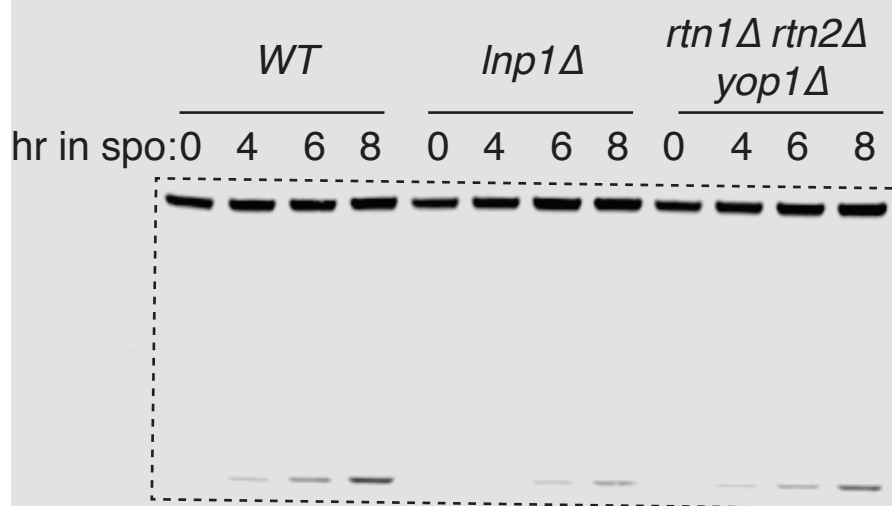

hexo

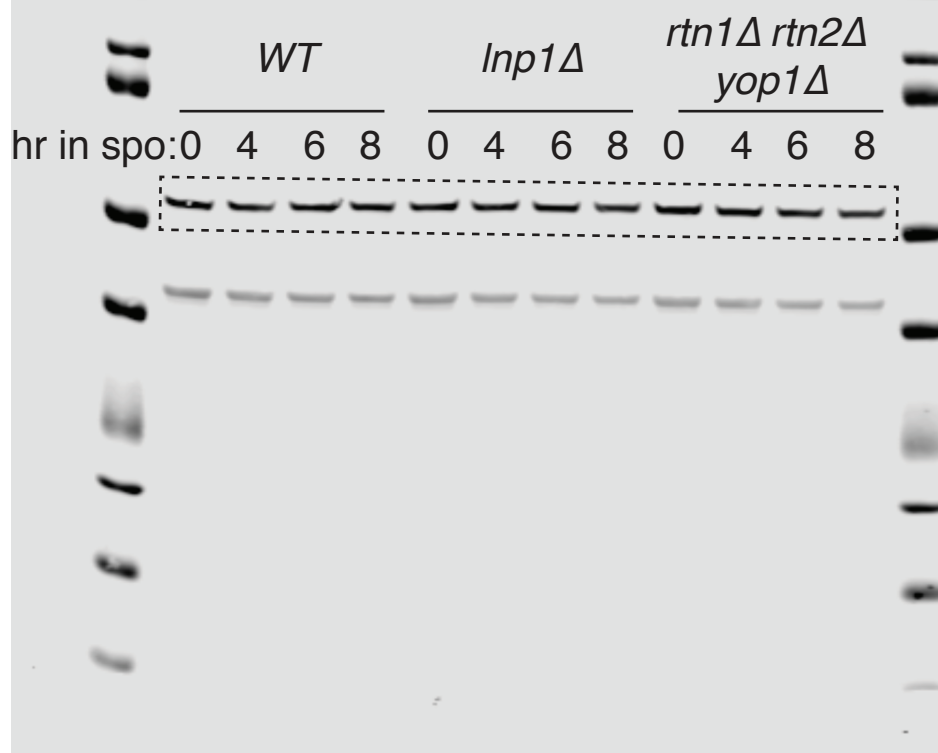

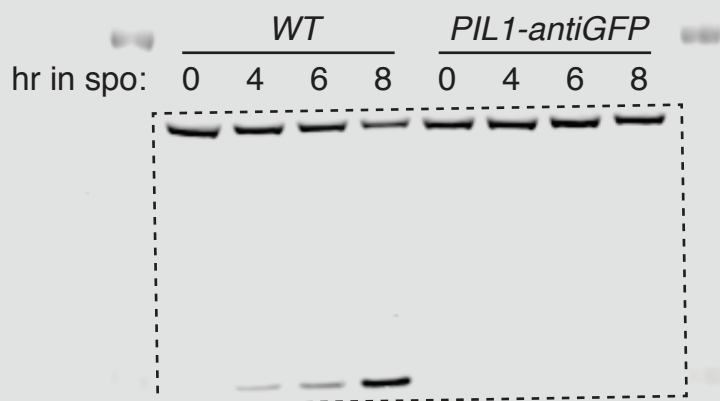

GFP

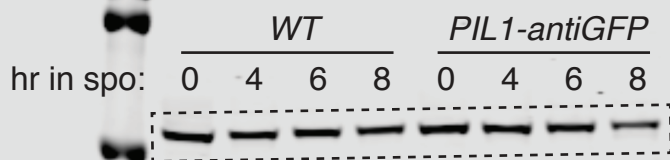

hexo

# Pil1-antiGFP

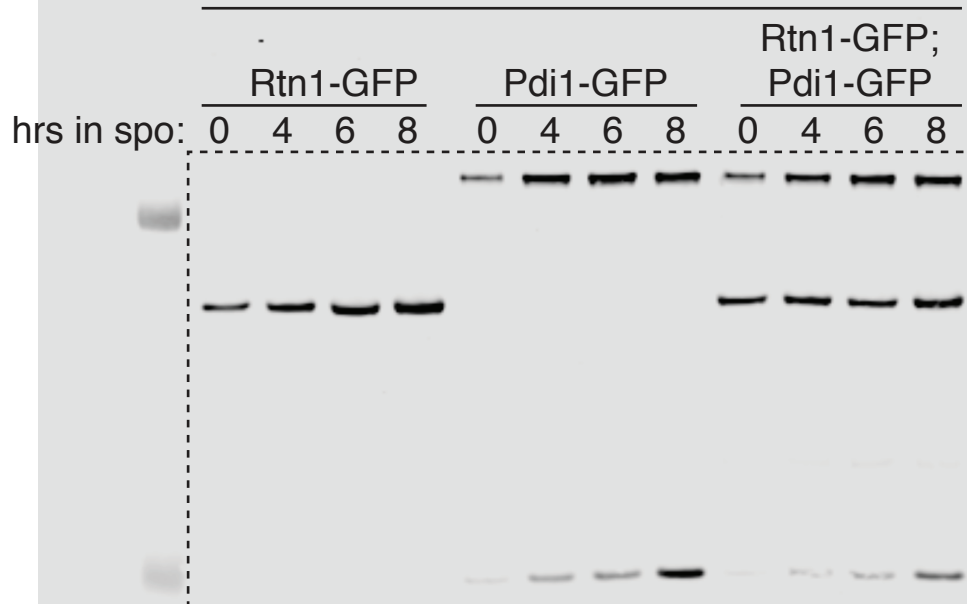

# Pil1-antiGFP

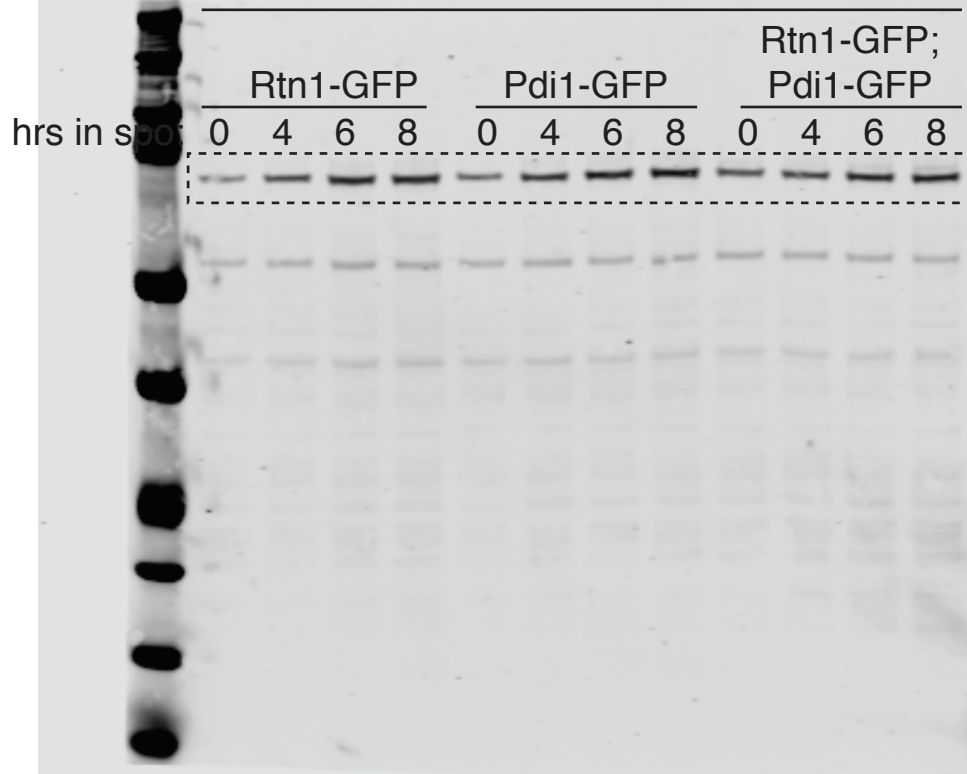

Supplement: SourceData F7 — contains original blots for Fig. 7. [file JCB_202108105_SourceDataF7.pdf]

GFP

hr in spo: 0 2 4 5 6 8 9 10 11 12

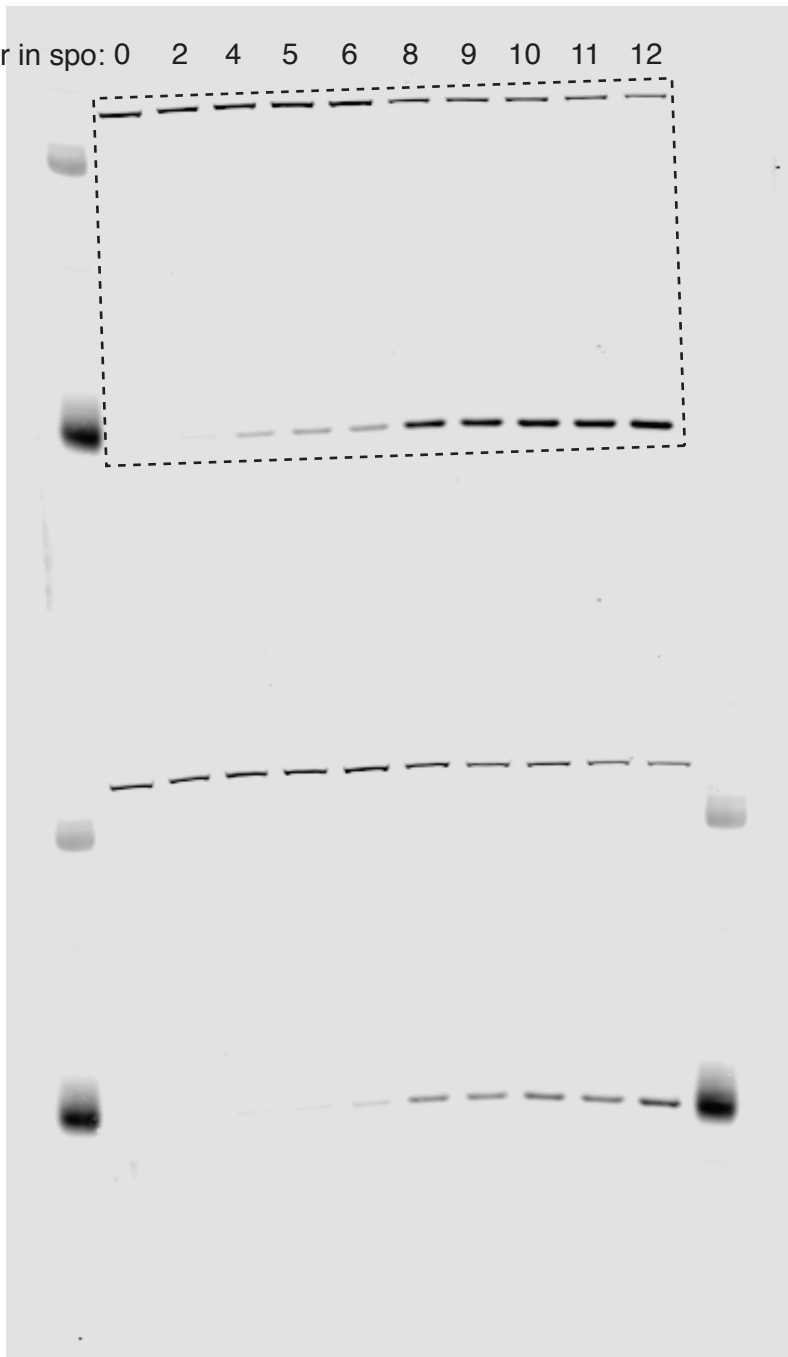

hexo

hr in spo: 0 2 4 5 6 8 9 10 11 12

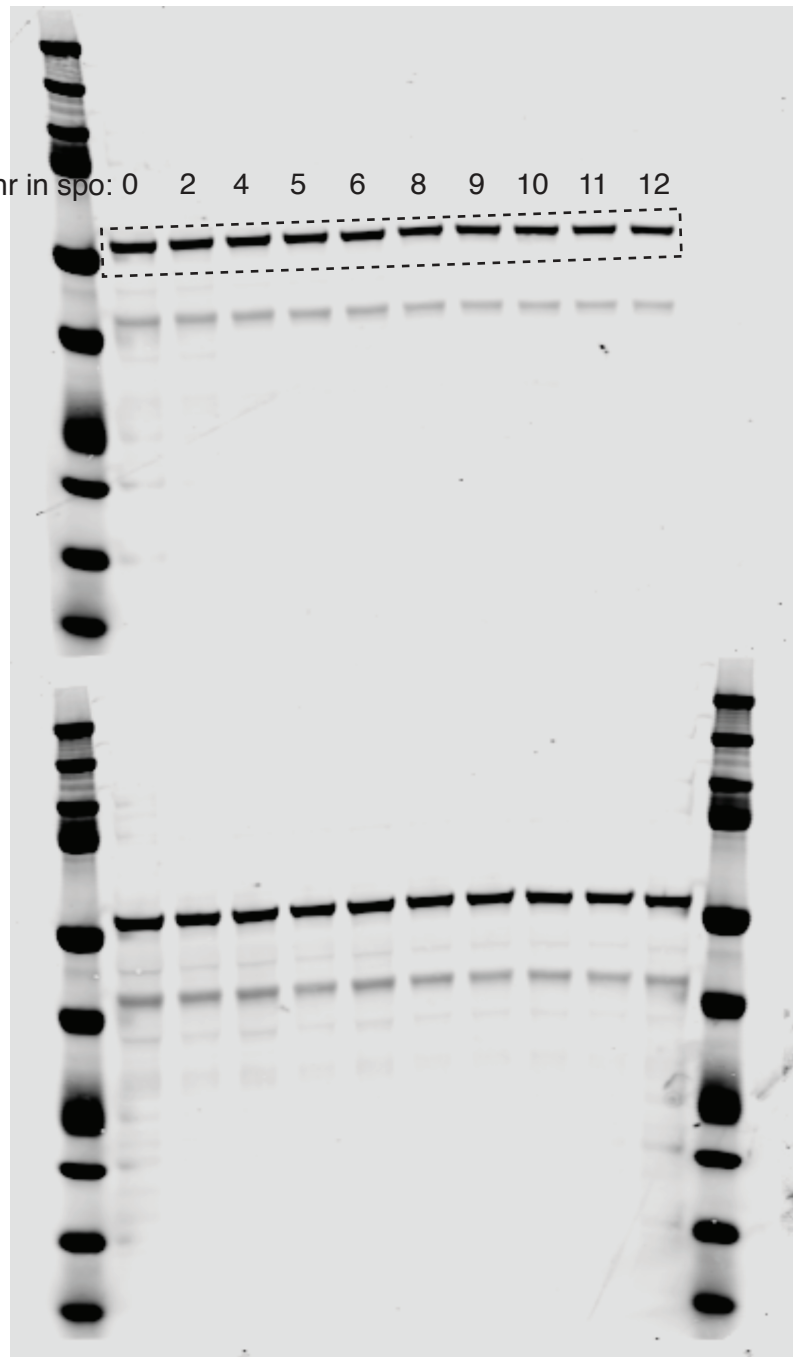

GFP

hexo

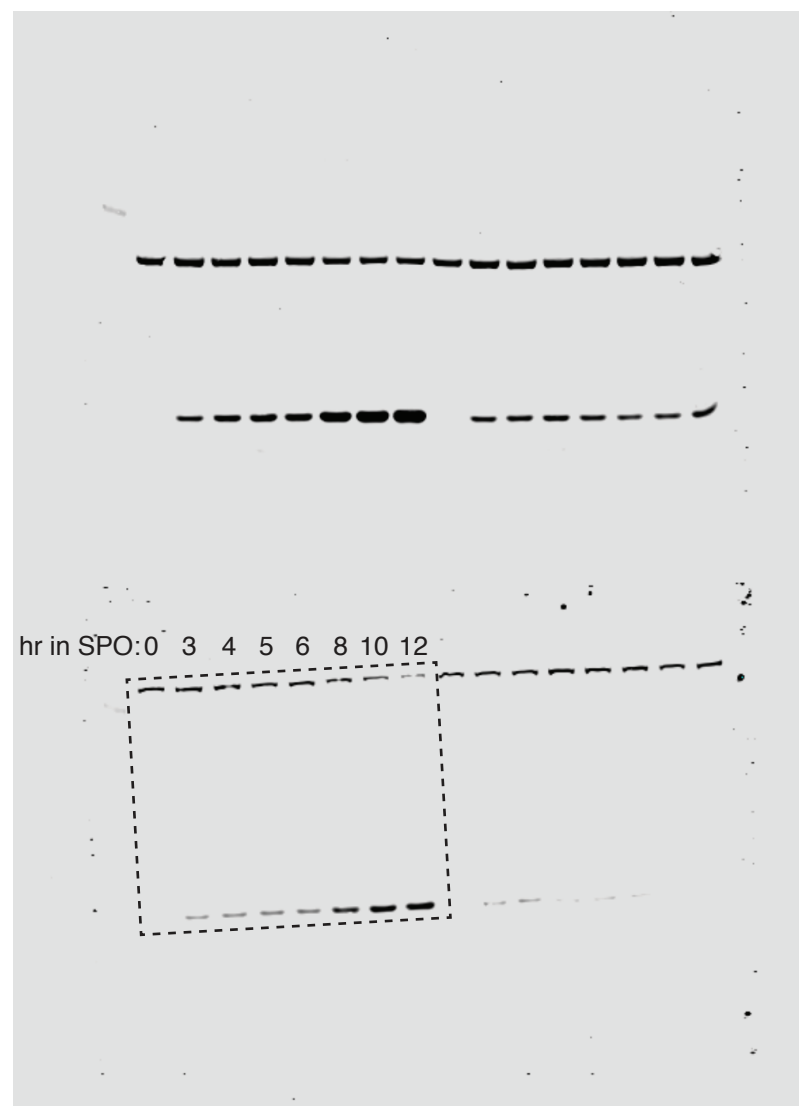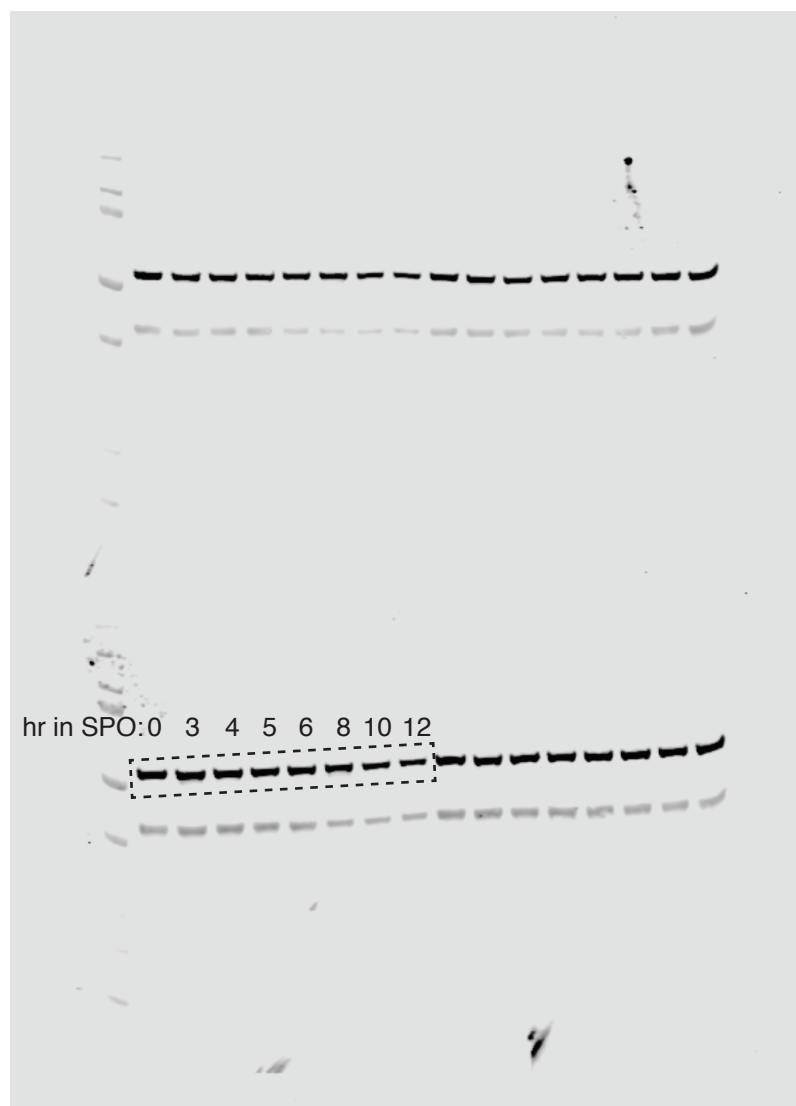

GFP

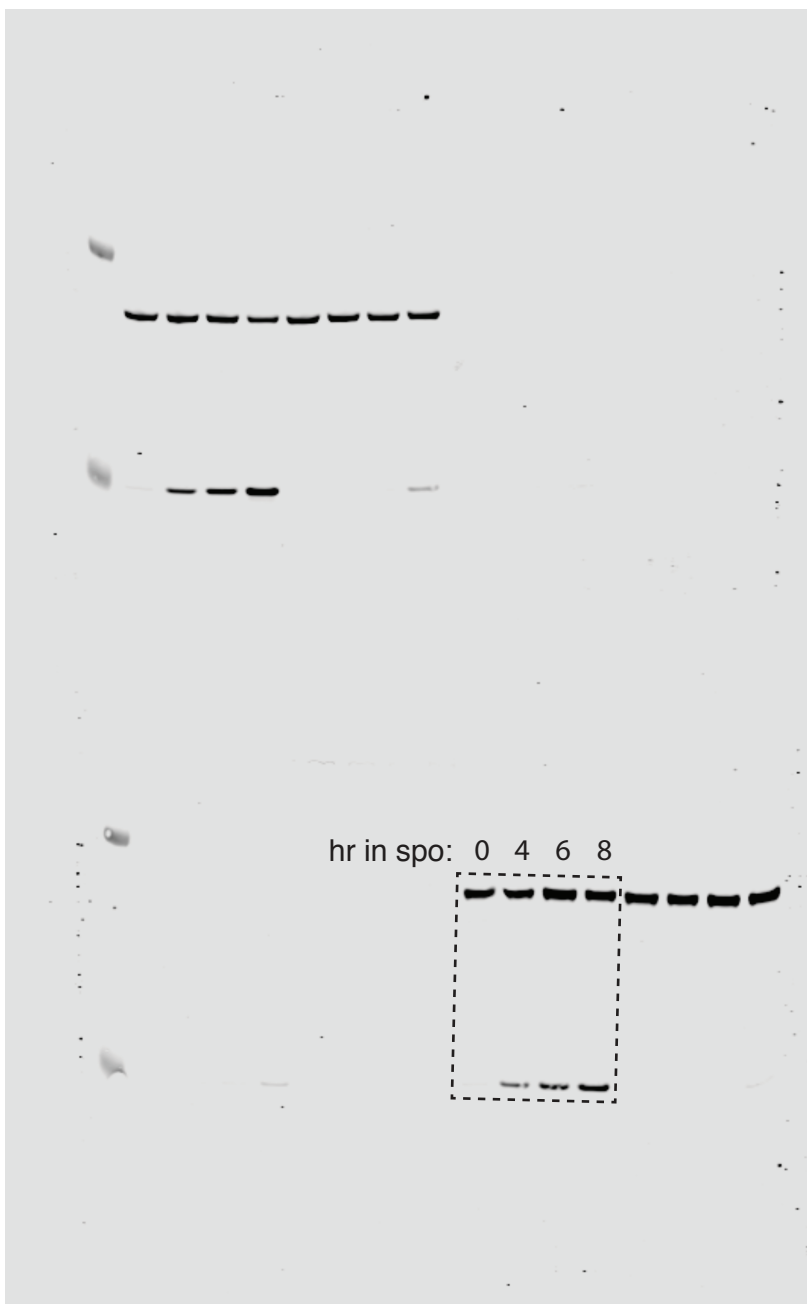

hexo

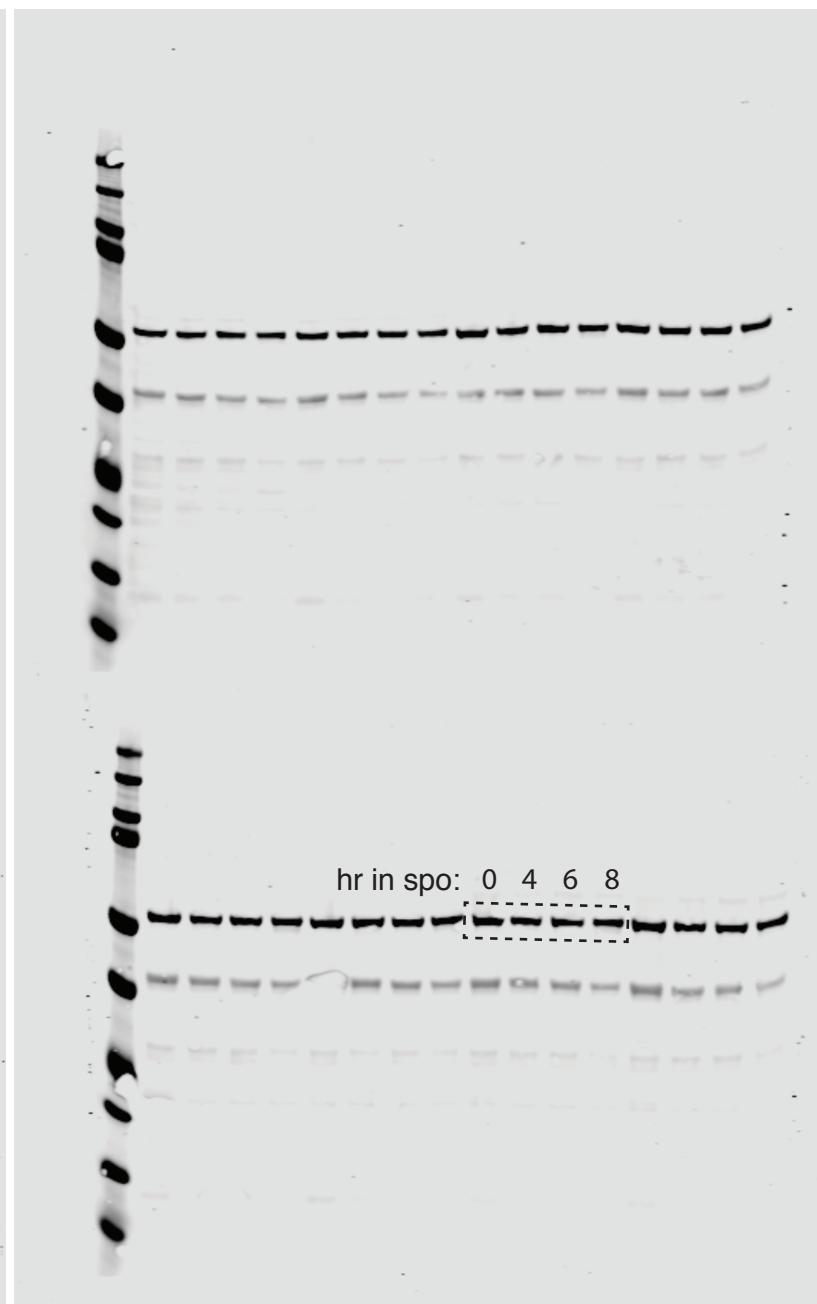

Supplement: SourceData FS4 — contains original blots for Fig. S4. [file JCB_202108105_SourceDataFS4.pdf]

*GFP*

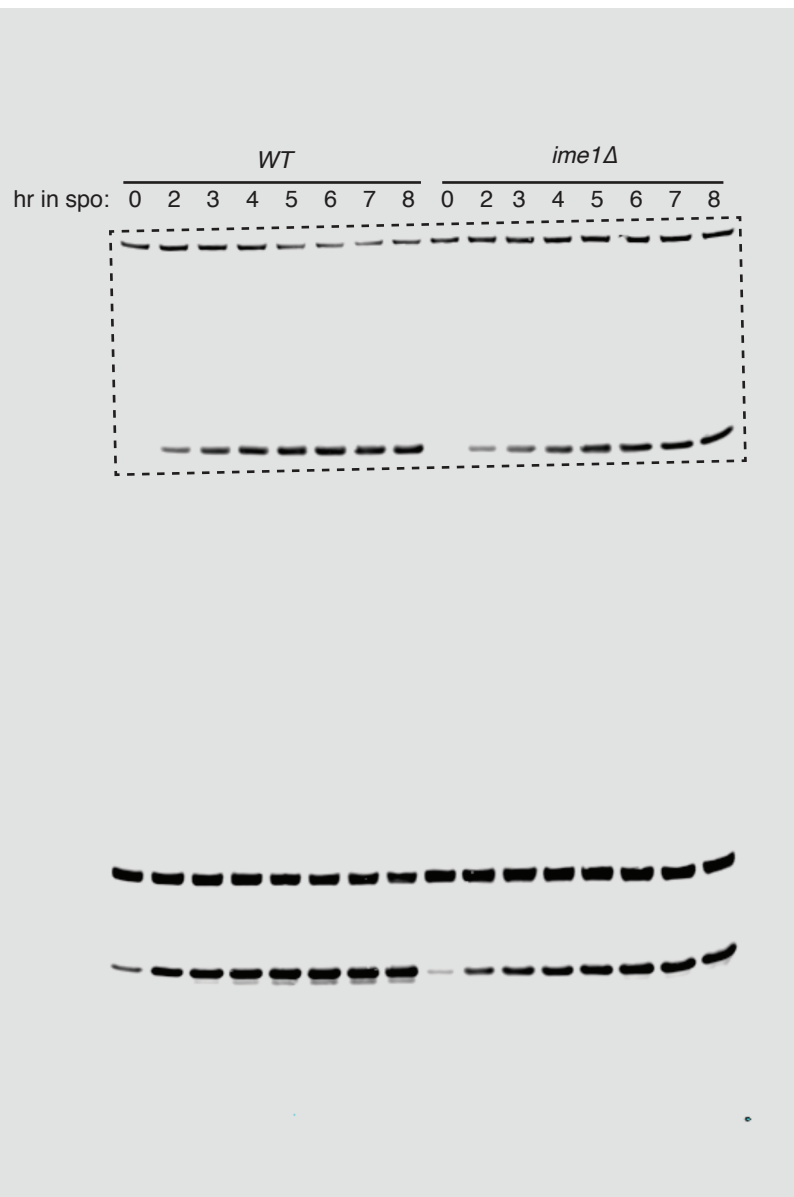

*hexo*

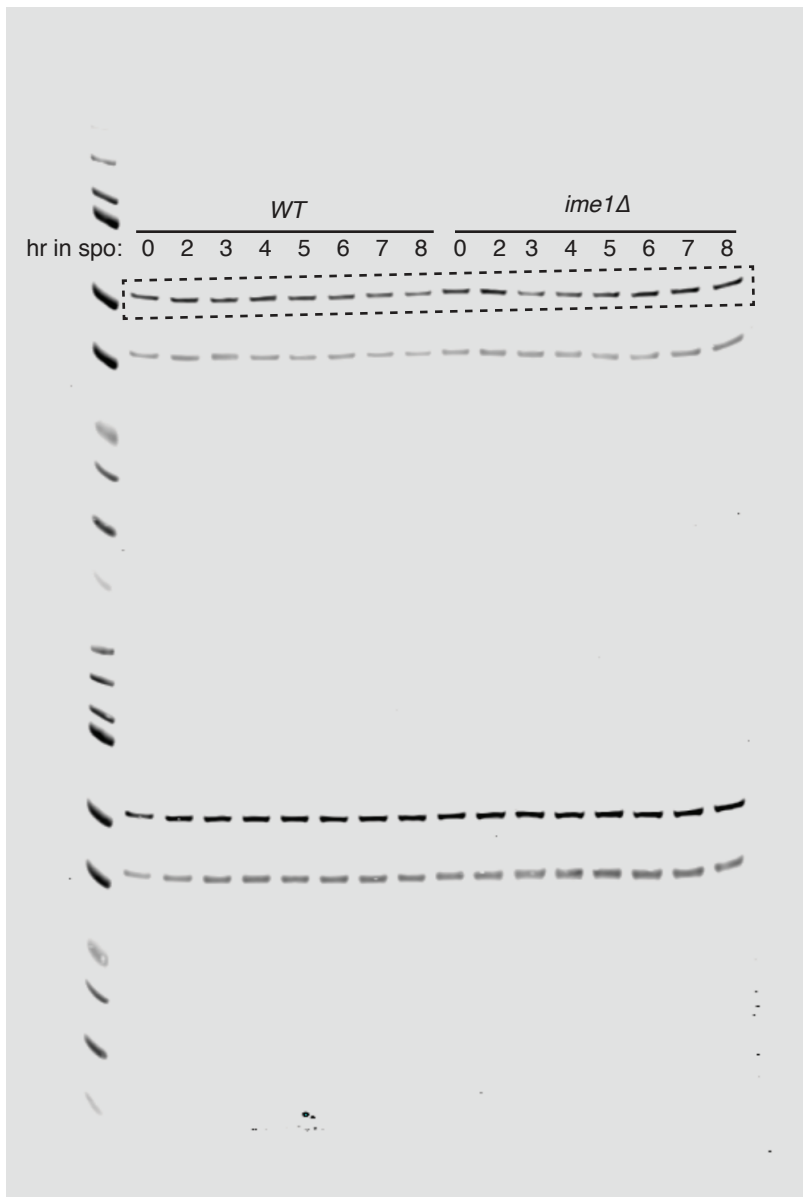



GFP

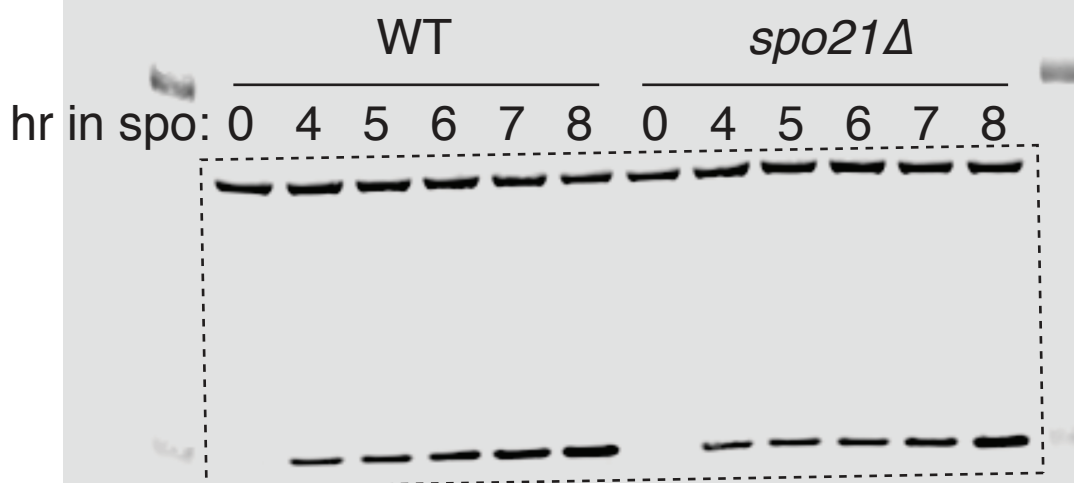

hexo

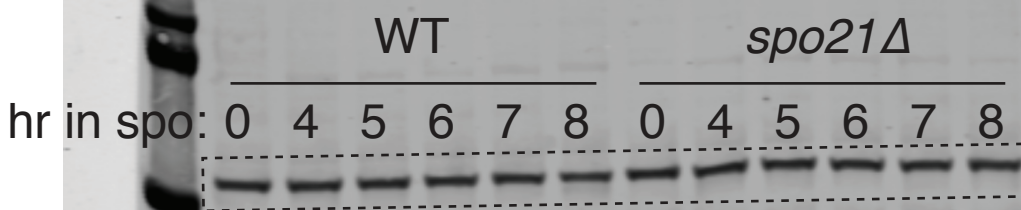

Supplement: SourceData FS5 — contains original blots for Fig. S5. [file JCB_202108105_SourceDataFS5.pdf]

# GFP

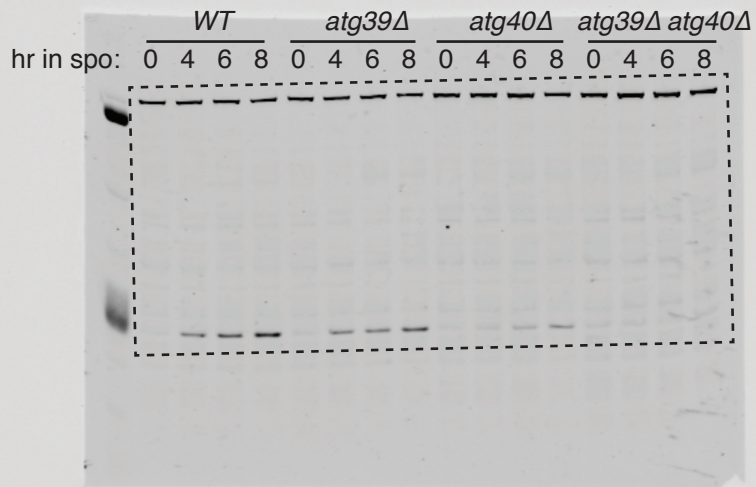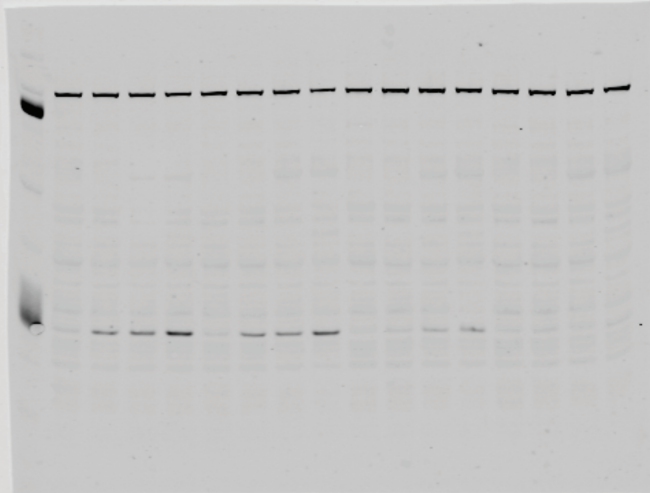

# hexo

hr in spo:      WT      atg39Δ      atg40Δ      atg39Δ atg40Δ

                 0 4 6 8    0 4 6 8    0 4 6 8    0 4 6 8

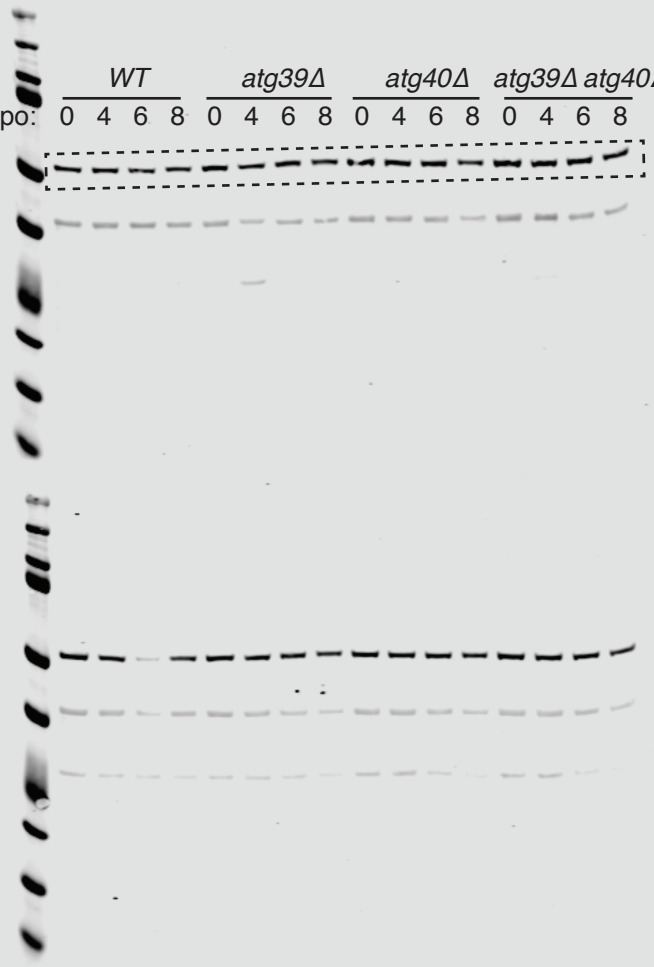

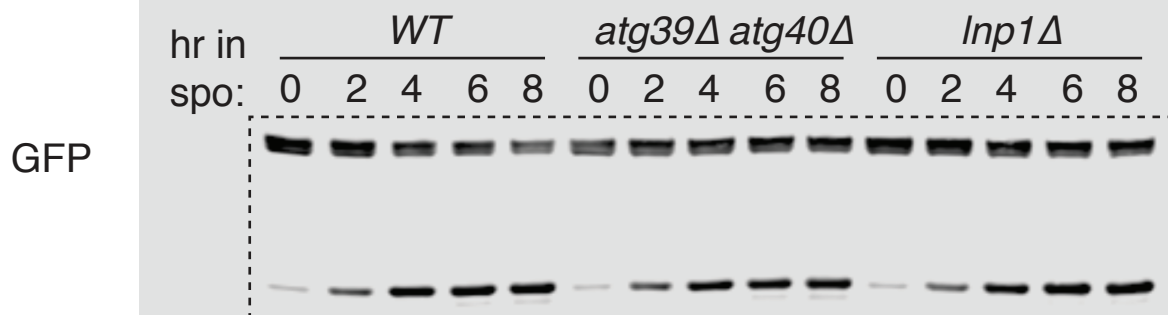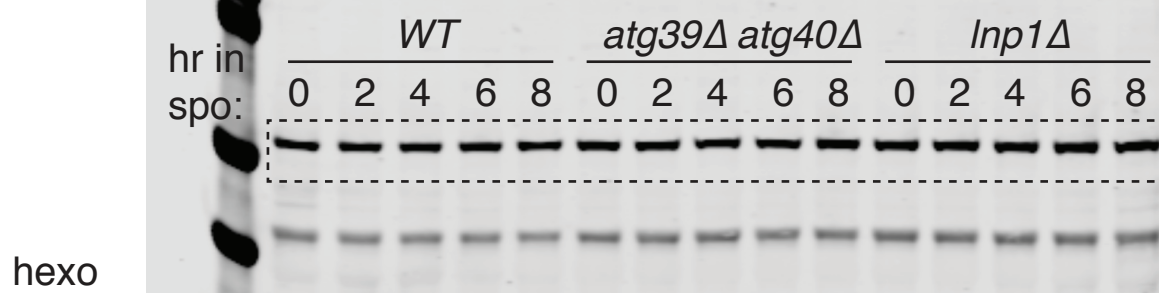

Supplement: SourceData FS6 — contains original blots for Fig. S6. [file JCB_202108105_SourceDataFS6.pdf]

*GFP*

*hexo*

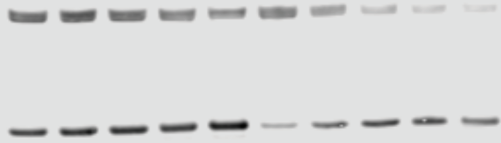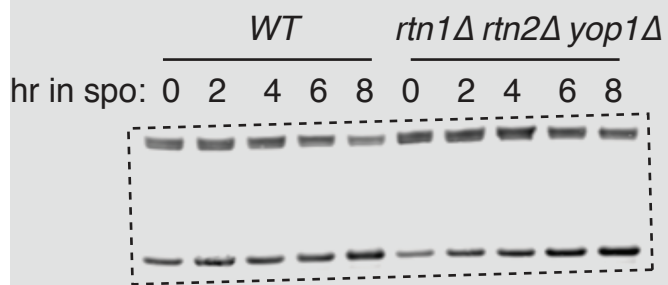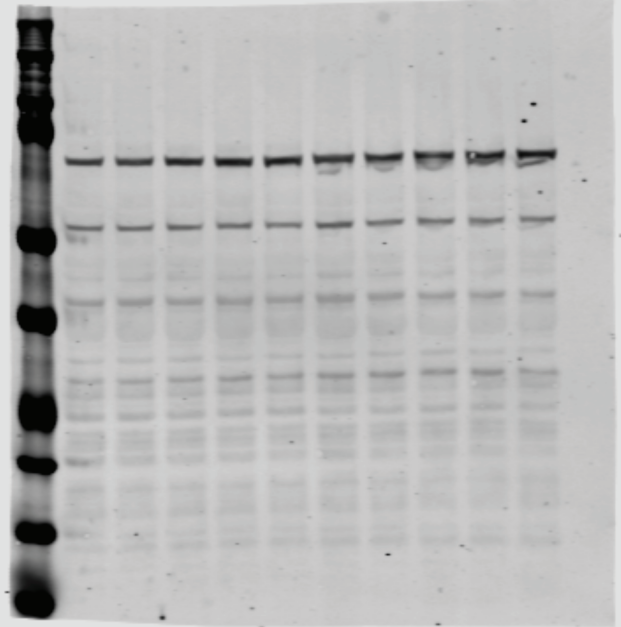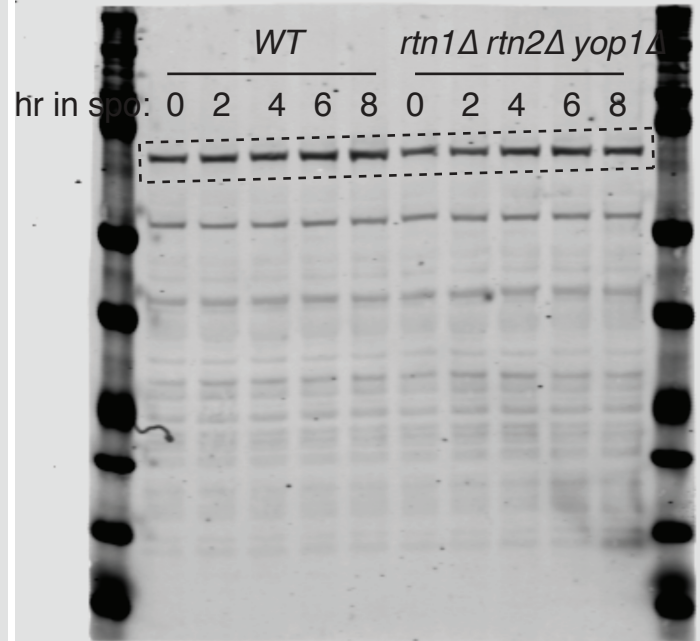

Supplement: SourceData FS7 — contains original blots for Fig. S7. [file JCB_202108105_SourceDataFS7.pdf]
